# DNA barcoding of oomycetes with cytochrome *c* oxidase subunit I and internal transcribed spacer

**DOI:** 10.1111/j.1755-0998.2011.03041.x

**Published:** 2011-11

**Authors:** Gregg P Robideau, Arthur W A M de Cock, Michael D Coffey, Hermann Voglmayr, Henk Brouwer, Kanak Bala, David W Chitty, Nicole Désaulniers, Quinn A Eggertson, Claire M M Gachon, Chia-Hui Hu, Frithjof C Küpper, Tara L Rintoul, Ehab Sarhan, Els C P Verstappen, Yonghong Zhang, Peter J M Bonants, Jean B Ristaino, C André Lévesque

**Affiliations:** * Biology Department, Carleton University1125 Colonel By Dr., Ottawa, Ontario, Canada K1S 5B6; † Agriculture and Agri-Food Canada960 Carling Ave., Ottawa, Ontario, Canada K1A 0C6; ‡ CBS KNAW Fungal Biodiversity CentreUppsalalaan 8, 3584 CT Utrecht, The Netherlands; § Department of Plant Pathology and Microbiology, University of CaliforniaRiverside, CA 92521, USA; ¶ Systematic and Evolutionary Botany, Faculty Center Biodiversity, University of ViennaRennweg 14, 1030 Wien, Austria; ** Culture Collection for Algae and Protozoa, Scottish Association for Marine Science, Scottish Marine InstituteOban PA37 1QA, UK; †† Department of Plant Pathology, North Carolina State UniversityRaleigh, NC 27695, USA; ‡‡ Plant Research InternationalDroevendaalsesteeg 1, 6708 PB Wageningen, The Netherlands

**Keywords:** cytochrome *c* oxidase subunit I, DNA barcoding, internal transcribed spacer, oomycete, species identification

## Abstract

Oomycete species occupy many different environments and many ecological niches. The genera *Phytophthora* and *Pythium* for example, contain many plant pathogens which cause enormous damage to a wide range of plant species. Proper identification to the species level is a critical first step in any investigation of oomycetes, whether it is research driven or compelled by the need for rapid and accurate diagnostics during a pathogen outbreak. The use of DNA for oomycete species identification is well established, but DNA barcoding with cytochrome *c* oxidase subunit I (COI) is a relatively new approach that has yet to be assessed over a significant sample of oomycete genera. In this study we have sequenced COI, from 1205 isolates representing 23 genera. A comparison to internal transcribed spacer (ITS) sequences from the same isolates showed that COI identification is a practical option; complementary because it uses the mitochondrial genome instead of nuclear DNA. In some cases COI was more discriminative than ITS at the species level. This is in contrast to the large ribosomal subunit, which showed poor species resolution when sequenced from a subset of the isolates used in this study. The results described in this paper indicate that COI sequencing and the dataset generated are a valuable addition to the currently available oomycete taxonomy resources, and that both COI, the default DNA barcode supported by GenBank, and ITS, the de facto barcode accepted by the oomycete and mycology community, are acceptable and complementary DNA barcodes to be used for identification of oomycetes.

## Introduction

Oomycetes are fungal-like organisms that are found in a wide range of environments and ecological niches. They are classified among the stramenopiles (=Straminipila), a lineage including brown algae and diatoms that has lost plastids and is very distant phylogenetically from the kingdom Eumycota, the true Fungi. Many oomycete species are pathogens of plants and animals. The devastating speed with which they are able to spread makes rapid detection and identification crucial to implementation of control strategies. Biocontrol of oomycetes is an active area of study, and there are examples of oomycete species that are used as biological control against other oomycetes (Jones & Deacon 1995; Picard *et al.* 2000), exemplifying the range of ecological functions between species.

Due to their wide variety of ecological roles, broad distribution and economic impact, proper identification is of great importance in oomycete studies. Identification of species can be a laborious and difficult task requiring time and expertise to cultivate the distinguishing morphological characters and compare them by microscopy. Also the decreasing number of experts able to identify oomycetes by morphological features is an important factor. Although matrix-based Lucid keys are being developed that will improve the speed of identification by morphology (Abad & Coffey 2008; Ristaino *et al.* 2008), DNA-based identification can be done quickly and easily by a nonspecialist, achieving accurate results in a fraction of the time if there is an adequate database of reference strains.

Currently the most common region of DNA being used for identification of oomycetes to the species level is the internal transcribed spacer (ITS) region of rDNA. The ITS region in oomycetes is easy to amplify for DNA sequencing in most species with the use of universal eukaryotic PCR (polymerase chain reaction) primers (White *et al.* 1990; Ristaino *et al.* 1998). Cooke *et al.* (2000) were the first to publish a database of ITS sequences that covered all the known and available species of an oomycete genus. ITS then became the de facto DNA barcode for identification of *Phytophthora* species and similar comprehensive databases for *Pythium* (Lévesque & de Cock 2004) and downy mildews (Voglmayr 2003) followed. However, due to the apparent lack of functional constraint on this untranslated region of rDNA, alignment of ITS sequences is hampered by large amounts of insertions and deletions, which can be an issue for accurate comparisons. Indels in the ITS can even be observed within a single strain due to differences in alleles or differences among the multiple copies of the ITS, making direct sequencing of PCR products impossible (Kageyama *et al.* 2007). In some species of downy mildews, excessive length due to long insertions can raise difficulties when sequencing the complete ITS region. There are also certain cases where the ITS sequences of formally described species are extremely similar, particularly when they are evolutionarily closely related such as *Phytophthora infestans*, *Phytophthora phaseoli*, *Phytophthora ipomoeae*, *Phytophthora* sp. ‘*andina*’ and *Phytophthora mirabilis* (Gomez-Alpizar *et al.* 2008) which are 99.9% similar in ITS sequence (Kroon *et al.* 2004). Due to these limitations of the ITS region for identification, the use of another region for this purpose may lend more clarity to the molecular depictions of oomycete taxonomy.

Cytochrome *c* oxidase subunit I (COI, *COX1*) is a mitochondrially encoded gene which is recognized as an extremely useful DNA barcode capable of accurate species identification in a very broad range of eukaryotic life forms (Hebert *et al.* 2004; Ward *et al.* 2005; Hajibabaei *et al.* 2006; Seifert *et al.* 2007). COI is the default DNA barcode approved by GenBank and the Consortium for the Barcode of Life (CBOL) and it must be proven ineffective as a DNA barcode to be rejected as such. COI has proven useful in phylogenetic studies of the oomycete genus *Phytophthora* (Martin & Tooley 2003; Kroon *et al.* 2004), and the success of COI barcoding in red algae (Saunders 2005) made it a very intriguing prospect for barcoding of all oomycetes due to their algal ancestry. Because COI is a protein-coding region, alignment of COI sequences is simple and devoid of gaps if introns are absent. With the use of primers that amplify the 5′ end of COI, accurate species delimitation has been achieved with sequences of only 650 base pairs (bp) or less (Meusnier *et al.* 2008). With the advent of massively parallel sequencing from environmental samples, it is important to compare COI and ITS as the marine and animal science communities appear to have a strong interest in COI, whereas ITS is the established species-level marker in the mycology community, although not formally approved as a DNA barcode yet. Here we report the utility of COI sequence data for accurate species delimitation in oomycetes, and compare COI identification to the benchmark of ITS identification with 1205 isolates representing 23 genera including the recently described genus *Phytopythium* (formerly *Pythium* Clade K) (Bala *et al.* 2010b). Nearly all the currently described species of the two largest genera that can be maintained in culture (*Pythium* and *Phytophthora*) have been included in this study. In addition to COI and ITS, the D1–D3 region of nuclear large subunit (LSU) rDNA, a commonly used marker for phylogeny and identification of oomycetes and Fungi, was sequenced from a subset of 388 isolates from 20 genera and is analysed in comparison with COI and ITS. The complete list of isolates used for this study is shown in Table S1 (Supporting information).

## Materials and methods

The majority of isolates used for this study were processed by the primary methods summarized below. Additional methods that were used for a small proportion of isolates are described in Text S2 (Supporting information).

### DNA extraction

Extraction methods varied depending on the source of the cultures. For cultures grown from the Centraalbureau voor Schimmelcultures (CBS), mycelia from 5 to 14 day old liquid cultures grown in pea broth (de Cock *et al.* 1992) were harvested by vacuum filtration, freeze dried, and DNA was extracted following the protocol of Möller *et al.* (1992). For cultures grown from the Canadian Collection of Fungal Cultures (CCFC), mycelia from 5 to 14 day old liquid cultures grown in potato dextrose broth (Difco) at room temperature were removed from broth and DNA was extracted following the protocol of Möller *et al.* with a modification to the tissue lysis step. Instead of grinding mycelia in liquid nitrogen, mycelia were placed in 2 mL screw cap tubes containing 300 mg of zirconium oxide spheres and one 6 mm zirconium oxide sphere (Fox Industries), along with TES buffer (100 mm Tris pH 8.0, 10 mm EDTA, 2% SDS) and proteinase K. Lysis was achieved by placing tubes in a FastPrep® machine (BIO 101) for 45 s at speed 4.0. Tubes were incubated at 65 °C for 1 h and subsequent steps were performed following the original protocol. At the final step, DNA pellet was resuspended in 0.1× TE buffer containing 20 μg/mL RNase A and tubes were incubated at 65 °C for 10 min.

### DNA amplification

Sequencing templates were amplified from DNA extract using the universal eukaryotic primers UN-up18S42 (5′-CGTAACAAGGTTTCCGTAGGTGAAC-3′) (Bakkeren *et al.* 2000) and the new UN-lo28S1220 (5′-GTTGTTACACACTCCTTAGCGGAT-3′) (Bala *et al.* 2010a) for the combined ITS and LSU regions (Lévesque & de Cock 2004). In some cases the ITS region alone was amplified using UN-up18S42 and UN-lo28S22 (5′-GTTTCTTTTCCTCCGCTTATTGATATG-3′) (Lévesque & de Cock 2004). The oomycete-specific primers OomCoxI-Levup (5′-TCAWCWMGATGGCTTTTTTCAAC-3′) and Fm85mod (5′-RRHWACKTGACTDATRATACCAAA-3′), modified from Martin & Tooley (2003), were designed to amplify 727 bp from the 5′ end of COI mitochondrial DNA. In some cases, an alternative reverse primer, OomCoxI-Levlo (5′-CYTCHGGRTGWCCRAAAAACCAAA-3′), was used with OomCoxI-Levup, amplifying a slightly smaller 680 bp fragment of COI, perfectly overlapping the standard DNA barcode used in other groups. PCR reaction volume was 10 μL containing final concentrations of 1× Titanium Taq buffer (with 3.5 mm MgCl_2_), 0.1 mm dNTPs, 0.08 μm each of forward and reverse primer, 0.5× Titanium Taq polymerase, and ∼1–10 ng/μL of DNA. Reaction volume was brought up to 10 μL with sterile HPLC water. Thermocycler program for amplification of the ITS/LSU region was: 95 °C for 3 min followed by 40 cycles of 95 °C for 30 s, 68 °C for 45 s, 72 °C for 2 min. A final extension was made at 72 °C for 8 min. Program for ITS alone was identical to that for ITS/LSU, except for a shorter extension time of 90 s at 72 °C in each cycle. Program for amplification of the COI region was: 95 °C for 2 min followed by 35 cycles of 95 °C for 1 min, 55 °C for 1 min, 72 °C for 1 min. A final extension was made at 72 °C for 10 min.

### Sequencing amplification

Amplification of PCR products for sequencing was done with ABI Big Dye Terminator version 3.1 in a reaction volume of 10 μL, with Big Dye Seq Mix diluted 1:8 with Seq buffer. Final concentrations of each reagent were 0.875× Sequencing buffer, 5% trehalose, 0.125× Big Dye Seq Mix and 0.16 μm primer. Reaction volume was brought to 10 μL with sterile HPLC water and 1 μL of PCR product was added directly from initial PCR amplification without purification. Thermocycler program for ITS/LSU was: 95 °C for 3 min followed by 40 cycles of 95 °C for 30 s, 58 °C for 40 s, 60 °C for 4 min. Program for COI was: 95 °C for 3 min followed by 40 cycles of 95 °C for 30 s, 50 °C for 20 s, 60 °C for 4 min. Sequencing primers for ITS were UN-up18S42 and UN-lo28S22. Sequencing primers for LSU were UN-up28S40 (5′-GCATATCAATAAGCGGAGGAAAAG-3′) (Schurko *et al.* 2003), UN-up28S577 (5′-CGTCTTGAAACACGGACCAAGGAG-3′) (Bala *et al.* 2010a), UN-lo28S576B (5′-CTCCTTGGTCCGTGTTTCAAGACG-3′) (Bakkeren *et al.* 2000) and UN-lo28S1220. Sequencing primers for COI were OomCoxI-Levup and Fm85mod or OomCoxI-Levlo.

### Sequencing

DNA sequences were generated from sequencing amplification reactions using the ABI Prism 3130*xl* Genetic analyzer. DNA sequences have been deposited in the Barcode of Life Data Systems (BOLD) and GenBank. Accession numbers for both databases are found in Table S1 (Supporting information).

### Sequence editing, alignment and cluster analysis

Sequence results were reviewed and edited using Seqman software (DNAStar) and alignments were made using muscle for COI and mafft for ITS and LSU (Edgar 2004; Katoh *et al.* 2005). mafft alignment of LSU was performed with the G-INS-i algorithm on the download Mac OS X version. mafft alignment of ITS was performed with the L-INS-i algorithm. The default maximum sequence allowance was raised from 1000 to 2000 by opening the mafft script in/usr/local/bin and changing line 762 from if [$nseq -gt 1000 -a $iterate -gt 1]; then to if [$nseq -gt 2000 -a $iterate -gt 1]; then. Alignments in fastA format were converted to nexus format with MacClade 4.06. Alignment of COI contained 680 characters, alignment of ITS contained 2068 characters, and alignment of LSU contained 1395 characters. No characters were excluded from analysis of any marker. Calculation of distance matrices and UPGMA hierarchical clustering was performed with paup 4.0b10. Bootstrap values were obtained from 1000 reps. Trees were formatted for Figs 2 and 3 using Dendroscope (Huson *et al.* 2007).

**Fig. 1:**
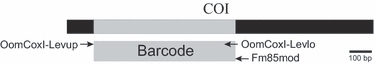
Diagram illustrating COI gene region, barcode segment of COI (grey) and COI PCR primer locations.

**Fig. 2:**
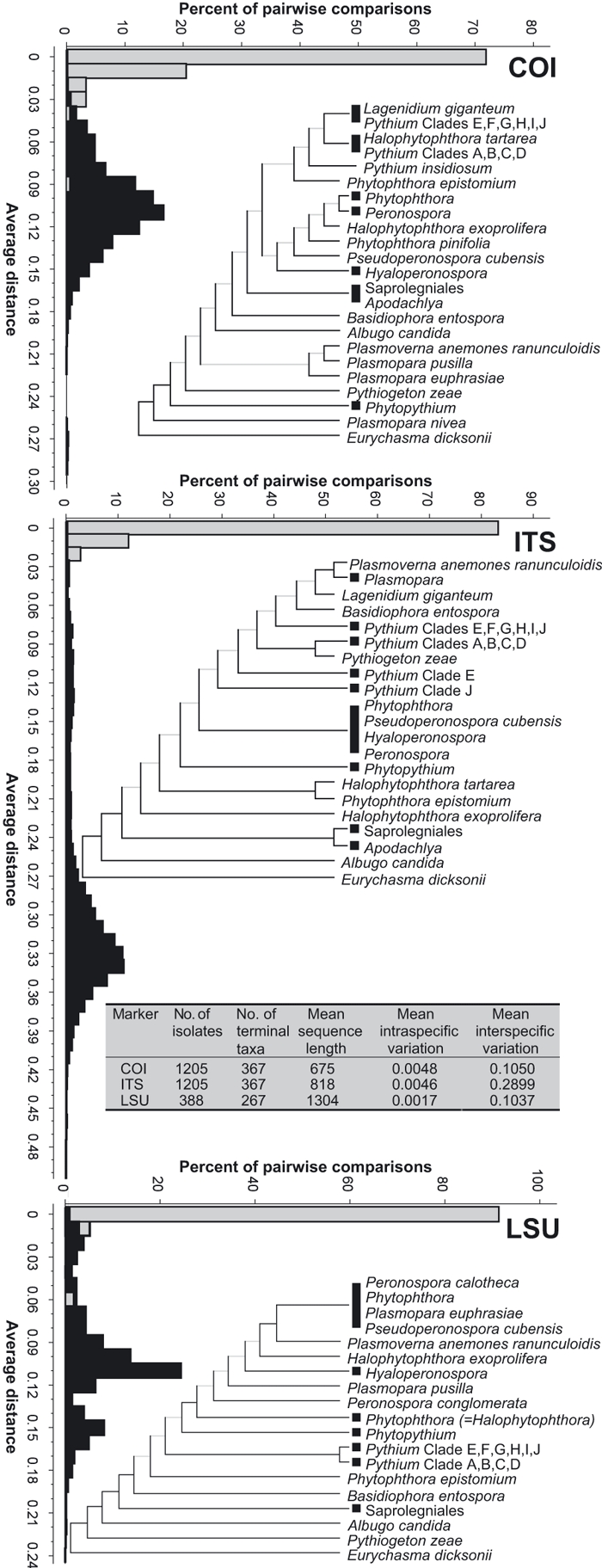
Phylograms and distance histograms for each marker. Black boxes at phylogram branch termini indicate multiple species. Branches with less than 50% bootstrap support are greyed out. Branch lengths are not to scale. Histograms display intraspecific variation in grey and interspecific variation in black. Inset table summarizes distance data.

**Fig. 3:**
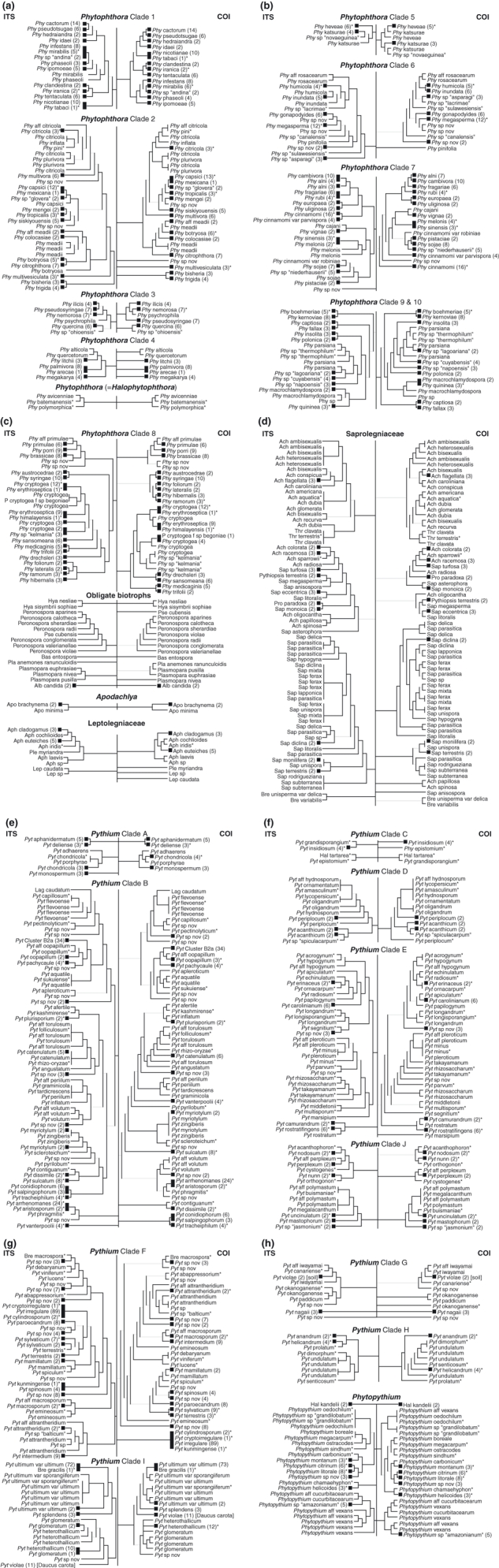
Direct comparison of ITS and COI phylograms by clade. ITS is shown on the left and COI on the right of an artificial vertical backbone. Black boxes at phylogram branch termini represent multiple isolates, with number of isolates shown in brackets. Asterisks denote ex-type specimen. Branches with less than 50% bootstrap support are greyed out. Branch lengths are not to scale. Most genus names are abbreviated to the first three letters. See Table S1 (Supporting information) for full names. (a) *Phytophthora* Clades 1, 2, 3, 4 and (=*Halophytophthora*). (b) *Phytophthora* Clades 5, 6, 7, 9 and 10. (c) *Phytophthora* Clade 8, Obligate biotrophs, *Apodachlya* and *Leptolegniaceae*. Note that grouping of all obligate biotroph isolates is superficial as they do not represent a coherent phylogenetic group. (d) Saprolegniaceae. (e) *Pythium* Clades A and B. (f) *Pythium* Clades C, D, E and J. (g) *Pythium* Clades F and I. (h) *Pythium* Clade G, H and *Phytopythium*.

### Distance matrix statistical analysis

Uncorrected ‘p’ (percentage) based distance matrices were analysed using matrix algebra and SAS. The average intraspecific distance was calculated for each species represented by more than one strain and coded as missing data when only one strain could be obtained to avoid having a bias towards zero variation. For each pair of species, the average pairwise distance was calculated for all the possible strain comparisons. A lower triangular uncorrected distance matrix was created with paup with the strains shown in Table S1 (Supporting information). The square pairwise distance matrix [PD] was imported into SAS as well as a column for species name (we did not have the same species name found in two different genera) and a column for corresponding strain coding. A 0/1 ‘dummy’ species variable design matrix [SV] was created in SAS using the species name column. The total of the distances [TD] for each species and pairwise comparison was found with the following equation: [TD] = [SV]^t^ × [PD] × [SV], where the diagonal was the number of pairwise comparisons for each species and the lower triangular matrix the total number of possible pairwise comparisons for each pair of species. A lower triangular matrix with a diagonal of 1’s [L1] was created with the same number of rows and columns as [PD]. The same equation as above was applied by replacing [PD] by [L1] to find the total number of pairwise distance comparisons [ND]. The average of all the pairwise comparisons was found by dividing [TD] by [ND], with the diagonal of the matrix giving the averages of all intraspecific comparisons and the lower matrix the averages of all interspecific comparisons. These values were used for distribution analyses.

## Results

### PCR primer performance

In an initial trial using the *Phytophthora* primers from Martin & Tooley (2003), consistent amplification of the COI barcode region was not achieved in a set of eight oomycete genera. However, the complete 5′ end and middle region of COI was sequenced with various combinations of their primers for *Saprolegnia*, *Achlya* and *Pythium* in addition to *Phytophthora*. Alignment of these sequences allowed design of new COI primers for the current study, OomCoxI-Levup and Fm85mod, which amplified a 727 bp fragment from the 5′ end of COI. For 18 isolates that did not amplify well with Fm85mod, an alternative reverse primer (OomCoxI-Levlo) was used. This amplified a smaller fragment of 680 bp compared to using Fm85mod (Fig. 1). Introns were not present in any COI sequence of the species studied. ITS fragments of varying length were used; from partial fragments as short as 402 bp from some *Pythium* isolates, up to 1351 bp from *Eurychasma*. In *Basidiophora*, *Plasmopara* and *Plasmoverna*, only the ITS1 region was sequenced due to long insertions in the ITS2 region. The LSU fragments ranged between 1246 and 1343 bp, although for three *Saprolegnia* isolates, partial fragments between 700 and 850 bp were used due to lack of high sequence quality for the entire D1–D3 region.

### Sequence distances

For each marker, distance matrices were used to calculate intraspecific (within species) variation, as well as interspecific (between species) variation. A graphical representation of the data and a table summarizing the results for all markers is shown in Fig. 2. The mean intraspecific variation for COI, ITS and LSU was 0.0048, 0.0046 and 0.0017, respectively. The mean interspecific variation was 0.1050, 0.2899 and 0.1037, respectively.

### Cluster analyses

Trees for each marker are shown in Fig. 2. Trees for COI and ITS contain 1205 sequences, including the basal oomycete *Eurychasma dicksonii* as the outgroup (Sekimoto *et al.* 2008). The LSU tree contains 388 sequences, including *E. dicksonii* as outgroup. Black squares at branch termini in Fig. 2 represent a collapsed subtree containing multiple species or in the case of the order Saprolegniales, multiple genera and species. Black rectangles at branch termini represent a clade of unresolved genera and indicate the presence of multiple species from the genera occupying the clade. Direct comparison between COI and ITS trees is shown in Fig. 3. Black squares at branch termini in Fig. 3 represent a collapsed subtree containing multiple isolates. Black rectangles at branch termini represent a clade of unresolved species and indicate the presence of multiple isolates from the species occupying the clade. Unresolved species with only single isolates are shown within clades represented by vertical lines at branch termini rather than rectangles. In Fig. 3, the genera *Phytophthora* and *Pythium* are divided and displayed by their previously established phylogenetic clades (Lévesque & de Cock 2004; Blair *et al.* 2008). Genera belonging to the families Saprolegniaceae and Leptolegniaceae are shown under the heading of their respective family. All obligate biotrophs are displayed together. Branch lengths in Figs 2 and 3 are not to scale, but full trees for each marker showing all isolates with scaled branch lengths and bootstrap values are given in Fig. S1 (Supporting information). For both COI and ITS, most isolates grouped into conspecific clusters, and the species composition of major clades did not differ between COI and ITS. Exceptions to this trend were *Phytophthora katsurae*, *Phytopythium* aff. *vexans*, *Pythium kunmingense* and *Pythium okanoganense*, which all appeared in different terminal nodes depending on the marker used. LSU sequences were more highly conserved and did not vary between some closely related species that were distinctly separate with COI and ITS. In some cases, two or more species shared identical or highly similar COI and ITS sequences, consistent across both markers, which invites further discussion of the possible synonymy of those species.

## Discussion

The primary purpose of the current study was to compare a validated oomycete de facto DNA barcode (ITS) with the default barcode (COI) which is officially accepted as the DNA barcode for eukaryotic groups unless proven ineffective. Our results indicate that both ITS and COI can be valid and useful barcodes for accurate identification of many oomycetes, whereas LSU more often lacks sufficient resolution between species. The genera *Pythium* and *Phytophthora* were almost completely covered by this study, and several other genera representing a wide range of oomycetes, including some obligate biotrophs, were partially covered. Intraspecific variation of COI is at par with that of ITS, although ITS does provide greater interspecific variation than COI. The benefit of COI barcoding is the ease of sequencing and aligning a relatively short fragment which has uniform length and can be amplified with degenerate primers throughout the entire oomycete class. This advantage over ITS is especially evident in the downy mildew genera *Basidiophora*, *Plasmopara*, *Plasmoverna* and relatives, which contain insertions in the ITS2 resulting in ITS sequences often longer than 2 kb (Thines 2007), raising difficulties to amplify, sequence and align the complete ITS region. LSU had the lowest interspecific variation of the three markers (Fig. 2), and the use of LSU as an oomycete barcode does not always provide enough resolution for identification to the species level. LSU appears to be better suited for studying genus- and family-level relationships in oomycetes (Riethmüller *et al.* 1999, 2002; Petersen & Rosendahl 2000; Voglmayr & Riethmüller 2006). A large portion of LSU was used to provide sufficient variation but this precludes amplification and sequencing with a single pair of primers. Barcoding with COI on the other hand, can quickly and easily lend additional evidence to identifications and new species descriptions by complementing nuclear DNA sequencing (ITS) with a mitochondrial DNA sequence (Bala *et al.* 2010a). The speed and ease of ITS and COI sequencing is also enhanced by the method of PCR amplification used in this study, which employed a minimal concentration of primers, thereby eliminating the need for purification of PCR products before sequencing. This approach, which was carried out in small PCR reaction volumes (10 μL), was able to reduce time and cost while still delivering high quality results.

Universality of PCR primers is also an important requirement of DNA barcode-based identification. The primers used for oomycete COI amplification (OomCoxI-Levup and Fm85mod) were able to amplify DNA from the entire range of oomycete genera in this study, including the basal genus *Eurychasma* and genera from the obligate biotrophic white blister rusts (*Albugo*) and downy mildews (*Basidiophora*, *Hyaloperonospora*, *Peronospora*, *Plasmopara*, *Plasmoverna* and *Pseudoperonospora*). There were, however, a few exceptional species of *Pythium* and *Phytopythium* (*Py. buismaniae*, *Py. contiguanum*, *Py. kashmirense*, *Py. ostracodes* and *Ph. cucurbitacearum*) that did not amplify with Fm85mod, and were instead amplified and sequenced using the alternative reverse primer OomCoxI-Levlo. Standard use of OomCoxI-Levlo is not recommended though, because our alignment of Fm85mod-derived COI sequences revealed that the 3′ end of OomCoxI-Levlo is not conserved throughout all *Pythium*, *Phytophthora* and *Aphanomyces* species.

Proposition of COI as a complement to ITS for species delimitation is based on the observation that relationships among closely related species and organization of major clades in *Pythium* and *Phytophthora* are concordant with the results of previous multilocus molecular studies (Kroon *et al.* 2004; Lévesque & de Cock 2004; Blair *et al.* 2008). Almost every terminal node on the UPGMA tree was composed of the same isolate(s) regardless of the marker used for sequencing. Replicated DNA sequencing of the isolates that did not follow this trend (*Phytophthora katsurae* P3389, *Phytopythium* aff. *vexans* CBS 261.30, *Pythium kunmingense* CBS 550.88 and *Pythium okanoganense* CBS 315.81) was performed to rule out the possibility of a DNA mix up during COI sequencing. In attempting to explain these situations biologically, the possibility of hybridization exists as has been well documented in *Phytophthora* (Érsek & Nagy 2008) and recently discovered in *Pythium* (Nechwatal & Mendgen 2008), but evidence of hybridization based on dimorphism in nuclear DNA sequence chromatograms was not found for any isolate mentioned above. An alternative scenario involving horizontal transfer of mitochondrial DNA (mtDNA) is not implausible based on previous findings in filamentous fungi. Mobile mitochondrial plasmids are prominent in filamentous fungi and they are known to recombine with mtDNA (Griffiths 1996). The presence of mitochondrial plasmids has not been documented in oomycetes, although it is interesting to note that a mobile plasmid derived from an intron of COI exists in the ascomycete fungus *Podospora anserina* (Osiewacz & Esser 1984). Fusion of hyphae (anastomosis), as has been reported in *Phytophthora* (Stephenson *et al.* 1974), could be a rare natural event that enables horizontal transfer of mtDNA in oomycetes. Although the true nature of the aforementioned results is unknown, it is worth stating that the use of both ITS and COI rather than one or the other, is recommended for taxonomic identification of oomycetes.

Considering that new species descriptions are a demanding process involving detailed morphological study, the ability to predict candidacy for a new species description with additional DNA sequence data will be very valuable and time-saving, providing more confidence so as to avoid questionable or synonymous species descriptions. Several putative new species are present in the isolates used for this study, denoted by the species epithet ‘sp. nov.’.

The augmented species resolution that COI provides is evident for arguably the most economically important oomycete, *Phytophthora infestans*. This species, which causes late blight of potato and tomato, has an ITS sequence that is indistinguishable from the closely related species *Phytophthora* sp. ‘*andina*’ and *Phytophthora mirabilis*. COI on the other hand, separates these three species into individual terminal nodes. The same situation has been seen between the strawberry pathogen *Phytophthora fragariae* and the recently circumscribed raspberry pathogenic species *Phytophthora rubi* (Man in ‘t Veld 2007), originally classified as *P. fragariae* var. *rubi*. While the ITS sequences do not vary between these two species, a clear distinction exists between their COI sequences. A similar example of species resolution by COI in *Pythium* is between the marine algal pathogens *P. chondricola* and *P. porphyrae*. Other examples of species resolution by COI are listed in Text S1A (Supporting information). The initial recognition of individuality between these species can be credited to examination of morphological characters and confirmation of these species descriptions by COI sequencing acknowledges the accuracy of morphological observation.

Though the use of COI sequencing is able to reinforce some species boundaries, there are several cases where formally described species are indistinguishable with either ITS or COI. Cases of apparent conspecificity are listed in Text S1B (Supporting information). Our results also implicate the existence of species complexes where gene flow may be occurring between species. In some cases there are several described species included in a complex, or alternatively a single described species may display substantial intraspecific variation, thus suggesting that a complex of multiple species exists within the single described species. For example, there is a large species complex referred to here and in Fig. 3e as *Pythium* Cluster B2a which includes *P. coloratum*, *P. diclinum*, *P.* cf. *dictyosporum*, *P. dissotocum*, *P. lutarium*, *P.* sp. ‘Group F’ and *P.* sp. ‘*tumidum*’*.* Other examples of species complexes in *Achlya*, *Phytophthora*, *Pythium*, *Phytopythium* and *Saprolegnia* are listed in Text S1C (Supporting information). Such complicated taxonomic situations are inextricable with a single marker, and it is therefore important to have additional evidence from other markers such as COI for taxonomic identification of oomycetes.

The conclusion drawn from this study is that COI sequencing is a very useful addition to the oomycete molecular toolbox which can now be used for identification of many oomycete species using the reference data generated by this study. In some of the most difficult cases of species concept in *Phytophthora* and *Pythium*, COI provides better resolution and support for current taxonomy than ITS does. However, because both markers provide an acceptable resolution when used individually, because of the history of using ITS in mycology and for oomycetes, and because it is desirable to have the complement of mitochondrial and nuclear markers, we are proposing that ITS be added to COI as a DNA barcode for oomycetes in GenBank. For any oomycete species that is not included in this study or for any new species to be described, both markers should be sequenced and deposited as barcodes.

## Data Accessibility

UPGMA trees are accessible in TreeBASE study S11552. Refer to Table S1 (Supporting information) for DNA sequence data accessibility.

## Supporting Information

**Table S1** Complete list of isolates used and accession numbers for their DNA sequences.

**Fig. S1** Full UPGMA trees for each marker showing all isolates with scaled branch lengths and bootstrap values. The COI tree spans pages 1–10, the internal transcribed spacer tree spans pages 11–20 and the LSU tree spans pages 21–23.
