# Supplementary material for: DNA barcoding of oomycetes with cytochrome c oxidase subunit I and internal transcribed spacer
Source: Mol Ecol Resour. 2011 Nov;11(6):1002–11. doi: 10.1111/j.1755-0998.2011.03041.x (PMC3195333; doi:10.1111/j.1755-0998.2011.03041.x)
Supplement: Supplementary file 1 [file men0011-1002-SD1.pdf]

Table S1. Complete list of isolates used and accession numbers for their DNA sequences.

Asterisks on isolate names denote ex-type specimens.

Genus Abbrev.	Isolate name	Isolate #	BOLD Process ID	Genbank Accessions
Ach	*Achlya ambisexualis*	CBS 101.50	OOMYA1184-08	HQ708156, HQ643083
Ach	*Achlya ambisexualis*	CBS 383.79	OOMYA1185-08	HQ708155, HQ643082
Ach	*Achlya americana*	CBS 100.52	OOMYA1186-08	HQ708157, HQ643084
Ach	*Achlya aquatica**	CBS 103.67	OOMYA1187-08	HQ708158, HQ643085
Ach	*Achlya bisexualis*	CBS 100.42	OOMYA1188-08	HQ708161, HQ643088
Ach	*Achlya bisexualis*	CBS 102.62	OOMYA1190-08	HQ708160, HQ643087
Ach	*Achlya bisexualis*	CBS 103.50	OOMYA1191-08	HQ708159, HQ643086
Ach	*Achlya caroliniana*	CBS 544.67	OOMYA1192-08	HQ708162, HQ643089
Ach	*Achlya colorata*	CBS 102.37	OOMYA1193-08	HQ708164, HQ643091
Ach	*Achlya colorata*	CBS 545.67	OOMYA1194-08	HQ708163, HQ643090
Ach	*Achlya conspicua*	CBS 103.37	OOMYA1195-08	HQ708165, HQ643092
Ach	*Achlya dubia*	CBS 101.38	OOMYA1196-08	HQ708167, HQ643094
Ach	*Achlya dubia*	CBS 546.67	OOMYA1197-08	HQ708166, HQ643093
Ach	*Achlya flagellata*	CBS 104.37	OOMYA1198-08	HQ708170, HQ643097
Ach	*Achlya flagellata*	CBS 107.35	OOMYA1199-08	HQ708169, HQ643096
Ach	*Achlya flagellata*	CBS 528.67	OOMYA1200-08	HQ708168, HQ643095
Ach	*Achlya glomerata*	CBS 105.50	OOMYA1201-08	HQ708171, HQ643098
Ach	*Achlya heterosexualis*	CBS 419.65	OOMYA1202-08	HQ708173, HQ643100
Ach	*Achlya heterosexualis*	CBS 420.65	OOMYA1203-08	HQ708172, HQ643099
Ach	*Achlya oligocantha*	CBS 101.44	OOMYA1204-08	HQ708174, HQ643101
Ach	*Achlya papillosa*	CBS 101.52	OOMYA1205-08	HQ708175, HQ643102
Ach	*Achlya racemosa*	CBS 103.38	OOMYA1206-08	HQ708178, HQ643105
Ach	*Achlya racemosa*	CBS 541.67	OOMYA1207-08	HQ708177, HQ643104
Ach	*Achlya racemosa*	CBS 578.67	OOMYA1208-08	HQ708176, HQ643103
Ach	*Achlya radiosa*	CBS 547.67	OOMYA1210-08	HQ708179, HQ643106
Ach	*Achlya recurva*	CBS 108.50	OOMYA1211-08	HQ708180, HQ643107
Ach	*Achlya sparrowii**	CBS 102.49	OOMYA1213-08	HQ708181, HQ643108
Ach	*Achlya spinosa*	CBS 576.67	OOMYA1214-08	HQ708182, HQ643109
Alb	*Albugo candida*	AC2V	OOMYA2013-10	HQ708184, HQ643111, HQ665049
Alb	*Albugo candida*	AC7A	OOMYA2014-10	HQ708183, HQ643110, HQ665050
Aph	*Aphanomyces cladogamus*	BR 693	OOMYA1218-08	HQ708187, HQ643114
Aph	*Aphanomyces cladogamus*	CBS 690.79	OOMYA1217-08	HQ708185, HQ643112
Aph	*Aphanomyces cladogamus*	CBS 108.29	OOMYA1215-08	HQ708186, HQ643113, HQ665056
Aph	*Aphanomyces cochlioides*	CBS 477.71	OOMYA1216-08	HQ708188, HQ643115, HQ665241
Aph	*Aphanomyces euteiches*	BR 694	OOMYA1219-08	HQ708193, HQ643120
Aph	*Aphanomyces euteiches*	CBS 155.73	OOMYA1221-08	HQ708191, HQ643118
Aph	*Aphanomyces euteiches*	CBS 157.73	OOMYA1223-08	HQ708189, HQ643116
Aph	*Aphanomyces euteiches*	CBS 154.73	OOMYA1220-08	HQ708192, HQ643119, HQ665129
Aph	*Aphanomyces euteiches*	CBS 156.73	OOMYA1222-08	HQ708190, HQ643117, HQ665132
Aph	*Aphanomyces iridis**	CBS 524.87	OOMYA1224-08	HQ708194, HQ643121, HQ665248
Aph	*Aphanomyces laevis*	CBS 478.71	OOMYA1226-08	HQ708195, HQ643122, HQ665242
Aph	*Aphanomyces* sp.	CBS 583.85	OOMYA1227-08	HQ708196, HQ643123, HQ665276
Apo	*Apodachlya brachynema*	CBS 184.82	OOMYA1229-08	HQ708197, HQ643124
Apo	*Apodachlya brachynema*	CBS 557.69	OOMYB040-08	HQ708198, HQ643125
Apo	*Apodachlya minima*	CBS 185.82	OOMYA1230-08	HQ708199, HQ643126
Bas	*Basidiophora entospora*	HV 119	DM011-10	HM033184, EF553487
Bas	*Basidiophora entospora*	HV 123		AY035513
Bre	*Brevilegnia gracilis**	CBS 131.37	OOMYA1232-08	HQ708200, HQ643127, HQ665122
Bre	*Brevilegnia macrospora**	CBS 132.37	OOMYA1908-08	HQ708201, HQ643128, HQ665124
Bre	*Brevilegnia unisperma* var. *delica*	CBS 143.52	OOMYA1233-08	HQ708202, HQ643129, HQ665125
Bre	*Brevilegnia variabilis*	CBS 110006	OOMYA1234-08	HQ708203, HQ643130, HQ665058
Eur	*Eurychasma dicksonii*	CCAP 4018/2	OOMYA2124-10	HQ708204, HQ665307
Eur	*Eurychasma dicksonii*	FI373	OOMYA2126-10	HQ643131
Hal	*Halophytophthora exoprolifera*	CBS 252.93	OOMYA1236-08	HQ708205, HQ643132, HQ665174
Hal	*Halophytophthora kandelii*	CBS 111.91	OOMYA006-07	HQ708207, HQ643134, HQ665065
Hal	*Halophytophthora kandelii*	CBS 113.91	OOMYA1237-08	HQ708206, HQ643133, HQ665079
Hal	*Halophytophthora tartarea**	CBS 208.95	OOMYA043-07	HQ708208, HQ643135
Hya	*Hyaloperonospora nesliae*	HV 203	DM004-10	HM033185, AY198250, EU054892
Hya	*Hyaloperonospora sisymbrii-sophiae*	HV 276	DM007-10	HM033186, AY198253, EU054910

Lag	*Lagenidium caudatum*	CBS 584.85	OOMYA199-07	HQ708209, HQ643136, HQ665277
Lag	*Lagenidium giganteum*	CBS 580.84	OOMYA2112-10	HQ708210
Lag	*Lagenidium giganteum*	ATCC 36492		AY151183
Lep	*Leptolegnia caudata*	CBS 680.69	OOMYA213-07	HQ708211, HQ643137, HQ665287
Lep	*Leptolegnia* sp.	CBS 582.85	OOMYA1238-08	HQ708212, HQ643138, HQ665275
	Peronospora aparines	HV 97	DM001-10	HM033187, AY198300
	Peronospora calotheca	HV 81	DM002-10	HM033188, AY198298
	Peronospora calotheca	HV 83		AY035483
	Peronospora conglomerata	HV 26	DM003-10	HM033189, AY198246
	Peronospora conglomerata	HV 27		AY035489
	Peronospora radii	HV 22	DM005-10	HM033190, AY198296
	Peronospora sherardiae	HV 211	DM006-10	HM033191, AY198301
	Peronospora valerianellae	HV 41	DM008-10	HM033192, AY198293
	Peronospora violae	HV 34	DM009-10	HM033193, AY198240
Phy	*Phytophthora* aff. *citricola*	BR 1073	OOMYA1250-08	HQ708271, HQ643204
Phy	*Phytophthora* aff. *infestans*	P13660	PHYTO099-10	HQ261238, HQ261491
Phy	*Phytophthora* aff. *meadii*	CBS 235.30	OOMYA2081-10	HQ708214, HQ643140
Phy	*Phytophthora* aff. *meadii*	CBS 238.28	OOMYA2082-10	HQ708213, HQ643139
Phy	*Phytophthora* aff. *primulae*	P6817	PHYTO203-10	HQ261239, HQ261492
Phy	*Phytophthora* aff. *rosacearum*	P10678	PHYTO065-10	HQ261240, HQ261493
Phy	*Phytophthora alni*	CBS 117375	OOMYA2060-10	HQ708217, HQ643143
Phy	*Phytophthora alni*	CBS 117376	OOMYA2061-10	HQ708216, HQ643142
Phy	*Phytophthora alni*	IMI 392314	OOMYA2163-10	GU945463, GU993881
Phy	*Phytophthora alni*	P10564	PHYTO055-10	HQ261244, HQ261497
Phy	*Phytophthora alni*	P11193	PHYTO084-10	HQ261243, HQ261496
Phy	*Phytophthora alni*	P11318	PHYTO086-10	HQ261242, HQ261495
Phy	*Phytophthora alni*	P16202	PHYTO126-10	HQ261241, HQ261494
Phy	*Phytophthora alticola*	P16052	PHYTO120-10	HQ261245, HQ261498
Phy	*Phytophthora arecae*	CBS 305.62	OOMYA115-07	HQ708218, HQ643146, HQ665200
Phy	*Phytophthora austrocedrae*	P15132	PHYTO108-10	HQ261247, HQ261500
Phy	*Phytophthora austrocedrae*	P16040	PHYTO117-10	HQ261246, HQ261499
Phy	*Phytophthora avicenniae*	CBS 188.85	OOMYA036-07	HQ708219, HQ643147, HQ665146
Phy	*Phytophthora batemanensis**	CBS 679.84	OOMYA212-07	HQ708220, HQ643148, HQ665286
Phy	*Phytophthora bisheria*	P11311	PHYTO085-10	HQ261249, HQ261502
Phy	*Phytophthora bisheria*	P7191	PHYTO217-10	HQ261248, HQ261501
Phy	*Phytophthora bisheria*	P10117	PHYTO013-10	HQ261250, HQ261503, EU080746
Phy	*Phytophthora boehmeriae*	P1257	PHYTO095-10	HQ261253, HQ261506
Phy	*Phytophthora boehmeriae*	P1378	PHYTO100-10	HQ261252, HQ261505
Phy	*Phytophthora boehmeriae*	p43	OOMYA2137-10	GU945465, GU993882
Phy	*Phytophthora boehmeriae*	P6950	PHYTO211-10	HQ261251, HQ261504, EU080166
Phy	*Phytophthora boehmeriae**	CBS 291.29	OOMYA105-07	HQ708221, HQ643149, HQ665190
Phy	*Phytophthora botryosa*	P1044	PHYTO050-10	HQ261257, HQ261510
Phy	*Phytophthora botryosa*	P3425	PHYTO169-10	HQ261256, HQ261509
Phy	*Phytophthora botryosa*	p44	OOMYA2138-10	GU945466, GU993883
Phy	*Phytophthora botryosa*	P6944	PHYTO209-10	HQ261255, HQ261508
Phy	*Phytophthora botryosa*	P6945	PHYTO210-10	HQ261254, HQ261507, EU079939
Phy	*Phytophthora botryosa**	CBS 581.69	OOMYA197-07	HQ708222, HQ643151
Phy	*Phytophthora brassicae*	CBS 112277	OOMYA2038-10	HQ708228, HQ643158
Phy	*Phytophthora brassicae*	CBS 180.87	OOMYA2074-10	HQ708224, HQ643154
Phy	*Phytophthora brassicae*	CBS 212.82	OOMYA2076-10	HQ708223, HQ643153
Phy	*Phytophthora brassicae*	CBS 782.97	OOMYA2115-10	HQ708226, HQ643156
Phy	*Phytophthora brassicae*	CBS 178.87	OOMYA034-07	HQ708225, HQ643155, HQ665144
Phy	*Phytophthora brassicae*	CBS 686.95	OOMYA2009-09	HQ708227, HQ643157, HQ665289
Phy	*Phytophthora brassicae*	P10155	PHYTO018-10	HQ261259, HQ261512, EU080794
Phy	*Phytophthora brassicae*	P3273	PHYTO164-10	HQ261258, HQ261511, EU079812
Phy	*Phytophthora cactorum*	BR 1067	OOMYA1249-08	HQ708240, HQ643170
Phy	*Phytophthora cactorum*	BR 194	OOMYA1242-08	HQ708239, HQ643169
Phy	*Phytophthora cactorum*	BR 672	OOMYA1245-08	HQ708237, HQ643167
Phy	*Phytophthora cactorum*	BR 673	OOMYA1246-08	HQ708236, HQ643166
Phy	*Phytophthora cactorum*	BR 675	OOMYA1248-08	HQ708235, HQ643165
Phy	*Phytophthora cactorum*	CBS 110121	OOMYA2019-10	HQ708238, HQ643168
Phy	*Phytophthora cactorum*	CBS 112275	OOMYA2037-10	HQ708234, HQ643164
Phy	*Phytophthora cactorum*	CBS 113344	OOMYA2039-10	HQ708233, HQ643163
Phy	*Phytophthora cactorum*	CBS 151.88	OOMYA2072-10	HQ708229, HQ643159
Phy	*Phytophthora cactorum*	CBS 231.30	OOMYA2078-10	HQ708231, HQ643161
Phy	*Phytophthora cactorum*	CBS 279.37	OOMYA2087-10	HQ708232, HQ643162
Phy	*Phytophthora cactorum*	CBS 294.29	OOMYA2089-10	HQ708230, HQ643160

Phy	*Phytophthora cactorum*	P10365	PHYTO043-10	HQ261260, HQ261513
Phy	*Phytophthora cactorum*	P0714	PHYTO008-10	HQ261261, HQ261514, EU080282
Phy	*Phytophthora cajani*	P3105	PHYTO157-10	HQ261262, HQ261515, EU080105
Phy	*Phytophthora cambivora*	CBS 111329	OOMYA2023-10	HQ708248, HQ643179
Phy	*Phytophthora cambivora*	CBS 114085	OOMYA2045-10	HQ708247, HQ643178
Phy	*Phytophthora cambivora*	CBS 114086	OOMYA2046-10	HQ708246, HQ643177
Phy	*Phytophthora cambivora*	CBS 114087	OOMYA2047-10	HQ708245, HQ643176
Phy	*Phytophthora cambivora*	CBS 114093	OOMYA2048-10	HQ708244, HQ643175
Phy	*Phytophthora cambivora*	CBS 114094	OOMYA2049-10	HQ708243, HQ643174
Phy	*Phytophthora cambivora*	CBS 114095	OOMYA2050-10	HQ708242, HQ643173
Phy	*Phytophthora cambivora*	CBS 356.78	OOMYA2100-10	HQ708241, HQ643172
Phy	*Phytophthora cambivora*	p64	OOMYA2142-10	GU945467, GU993885
Phy	*Phytophthora cambivora*	P0592	PHYTO006-10	HQ261263, HQ261516, EU080555
Phy	*Phytophthora capsici*	CBS 111333	OOMYA2024-10	HQ708256, HQ643188
Phy	*Phytophthora capsici*	CBS 111334	OOMYA2025-10	HQ708255, HQ643187
Phy	*Phytophthora capsici*	CBS 111335	OOMYA2026-10	HQ708254, HQ643186
Phy	*Phytophthora capsici*	CBS 111336	OOMYA2027-10	HQ708253, HQ643185
Phy	*Phytophthora capsici*	CBS 254.93	OOMYA2084-10	HQ708252, HQ643183
Phy	*Phytophthora capsici*	CBS 370.72	OOMYA2103-10	HQ708251, HQ643182
Phy	*Phytophthora capsici*	P3375	PHYTO167-10	HQ261265, HQ261518
Phy	*Phytophthora capsici*	P3605	PHYTO172-10	HQ261264, HQ261517
Phy	*Phytophthora capsici*	p8	OOMYA2144-10	GU945468, GU993886
Phy	*Phytophthora capsici*	CBS 554.88	OOMYA188-07	HQ708250, HQ643181, HQ665266
Phy	*Phytophthora capsici*	P10386	PHYTO047-10	HQ261267, HQ261520, EU079548
Phy	*Phytophthora capsici*	P1319	PHYTO097-10	HQ261266, HQ261519, EU079741
Phy	*Phytophthora capsici**	CBS 128.23	OOMYA012-07	HQ708249, HQ643180, HQ665120
Phy	*Phytophthora captiosa*	P10720	PHYTO073-10	HQ261268, HQ261521
Phy	*Phytophthora captiosa*	P10719	PHYTO072-10	HQ261269, HQ261522, EU079663
Phy	*Phytophthora cinnamomi*	CBS 232.30	OOMYA2079-10	HQ708263, HQ643195
Phy	*Phytophthora cinnamomi*	CBS 249.60	OOMYA2083-10	HQ708262, HQ643194
Phy	*Phytophthora cinnamomi*	CBS 270.55	OOMYA2085-10	HQ708261, HQ643193
Phy	*Phytophthora cinnamomi*	CBS 304.36	OOMYA2093-10	HQ708260, HQ643192
Phy	*Phytophthora cinnamomi*	CBS 319.49	OOMYA2097-10	HQ708266, HQ643198
Phy	*Phytophthora cinnamomi*	CBS 341.72	OOMYA2098-10	HQ708265, HQ643197
Phy	*Phytophthora cinnamomi*	CBS 342.72	OOMYA2099-10	HQ708264, HQ643196
Phy	*Phytophthora cinnamomi*	CBS 402.48	OOMYA2105-10	HQ708259, HQ643191
Phy	*Phytophthora cinnamomi*	CBS 403.48	OOMYA2106-10	HQ708258, HQ643190
Phy	*Phytophthora cinnamomi*	P2100	PHYTO147-10	HQ261277, HQ261530
Phy	*Phytophthora cinnamomi*	P2121	PHYTO148-10	HQ261276, HQ261529
Phy	*Phytophthora cinnamomi*	P2160	PHYTO151-10	HQ261275, HQ261528
Phy	*Phytophthora cinnamomi*	P2301	PHYTO152-10	HQ261274, HQ261527
Phy	*Phytophthora cinnamomi*	P3232	PHYTO162-10	HQ261273, HQ261526, EU079801
Phy	*Phytophthora cinnamomi*	P6305	PHYTO192-10	HQ261272, HQ261525, EU079898
Phy	*Phytophthora cinnamomi* var. *parvispora*	CBS 411.96	OOMYA154-07	HQ708268, HQ643200, HQ665231
Phy	*Phytophthora cinnamomi* var. *parvispora*	CBS 413.96	OOMYA155-07	HQ708267, HQ643199, HQ665232
Phy	*Phytophthora cinnamomi* var. *parvispora*	P7154	PHYTO216-10	HQ261271, HQ261524, EU080457
Phy	*Phytophthora cinnamomi* var. *parvispora*	P8495	PHYTO248-10	HQ261270, HQ261523, EU079953
Phy	*Phytophthora cinnamomi* var. *robiniae*	P16351	PHYTO129-10	HQ261278, HQ261531
Phy	*Phytophthora cinnamomi**	CBS 144.22	OOMYA017-07	HQ708257, HQ643189, HQ665126
Phy	*Phytophthora citricola*	BR 519	OOMYA1262-08	HQ708270, HQ643203
Phy	*Phytophthora citricola*	IIp52	OOMYA2127-10	GU945470, GU993888
Phy	*Phytophthora citricola*	P0716	PHYTO009-10	HQ261281, HQ261534
Phy	*Phytophthora citricola*	P0767	PHYTO010-10	HQ261280, HQ261533
Phy	*Phytophthora citricola*	P1805	PHYTO139-10	HQ261279, HQ261532
Phy	*Phytophthora citricola**	CBS 221.88	OOMYA059-07	HQ708269, HQ643201, HQ665161
Phy	*Phytophthora citrophthora*	136	OOMYA2012-10	GU945471, GU993889
Phy	*Phytophthora citrophthora*	CBS 111338	OOMYA2029-10	HQ708275, HQ643208
Phy	*Phytophthora citrophthora*	CBS 111339	OOMYA2030-10	HQ708274, HQ643207
Phy	*Phytophthora citrophthora*	CBS 111726	OOMYA2033-10	HQ708273, HQ643206
Phy	*Phytophthora citrophthora*	P10368	PHYTO044-10	HQ261282, HQ261535
Phy	*Phytophthora citrophthora*	CBS 950.87	OOMYA241-07	HQ708272, HQ643205, HQ665305
Phy	*Phytophthora citrophthora*	P10341	PHYTO041-10	HQ261283, HQ261536, EU080389
Phy	*Phytophthora clandestina*	P3943	PHYTO182-10	HQ261284, HQ261537

Phy	*Phytophthora clandestina*	P3942	PHYTO181-10	HQ261285, HQ261538, EU079871
Phy	*Phytophthora colocasiae*	P6290	PHYTO191-10	HQ261287, HQ261540, EU080128
Phy	*Phytophthora colocasiae*	P6317	PHYTO194-10	HQ261286, HQ261539, EU079911
Phy	*Phytophthora cryptogea*	BR 521	OOMYA410-07	HQ708284, HQ643219
Phy	*Phytophthora cryptogea*	BR 589	OOMYA1259-08	HQ708283, HQ643218
Phy	*Phytophthora cryptogea*	BR 682	OOMYA1266-08	HQ708282, HQ643217
Phy	*Phytophthora cryptogea*	CBS 114074	OOMYA2042-10	HQ708280, HQ643215
Phy	*Phytophthora cryptogea*	CBS 290.35	OOMYA2088-10	HQ708278, HQ643212
Phy	*Phytophthora cryptogea*	CBS 418.71	OOMYA2108-10	HQ708277, HQ643211
Phy	*Phytophthora cryptogea*	Lev 1802	OOMYA1268-08	HQ708279, HQ643214
Phy	*Phytophthora cryptogea*	p13	OOMYA2131-10	GU945473, GU993890
Phy	*Phytophthora cryptogea*	P16165	PHYTO125-10	HQ261294, HQ261547
Phy	*Phytophthora cryptogea*	P1693	PHYTO136-10	HQ261293, HQ261546
Phy	*Phytophthora cryptogea*	P1739	PHYTO137-10	HQ261292, HQ261545
Phy	*Phytophthora cryptogea*	P1810	PHYTO140-10	HQ261291, HQ261544
Phy	*Phytophthora cryptogea*	P3700	PHYTO174-10	HQ261289, HQ261542
Phy	*Phytophthora cryptogea*	P10705	PHYTO070-10	HQ261297, HQ261550
Phy	*Phytophthora cryptogea*	P1088	PHYTO079-10	HQ261296, HQ261549, EU080451
Phy	*Phytophthora cryptogea*	P11822	PHYTO090-10	HQ261295, HQ261548, EU080082
Phy	*Phytophthora cryptogea*	P3103	PHYTO156-10	HQ261290, HQ261543, EU080631
Phy	*Phytophthora cryptogea*	P3876	PHYTO178-10	HQ261288, HQ261541, EU079851
Phy	*Phytophthora cryptogea* f. sp. *begoniae*	CBS 468.81	OOMYA167-07	HQ708276, HQ643210, HQ665238
Phy	*Phytophthora cryptogea**	CBS 113.19	OOMYA007-07	HQ708281, HQ643216, HQ665075
Phy	*Phytophthora drechsleri*	P1087	PHYTO078-10	HQ261299, HQ261552
Phy	*Phytophthora drechsleri*	P11638	PHYTO087-10	HQ261298, HQ261551
Phy	*Phytophthora drechsleri*	P10331	PHYTO034-10	HQ261300, HQ261553, EU079511
Phy	*Phytophthora epistomium**	CBS 590.85	OOMYA203-07	HQ708285, HQ643220, HQ665279
Phy	*Phytophthora erythroseptica*	BR 464	OOMYA1272-08	HQ708291, HQ643227
Phy	*Phytophthora erythroseptica*	BR 664	OOMYA1273-08	HQ708290, HQ643226
Phy	*Phytophthora erythroseptica*	CBS 111343	OOMYA2031-10	HQ708292, HQ643228
Phy	*Phytophthora erythroseptica*	CBS 233.30	OOMYA2080-10	HQ708287, HQ643222
Phy	*Phytophthora erythroseptica*	CBS 951.87	OOMYA2120-10	HQ708289, HQ643225
Phy	*Phytophthora erythroseptica*	CBS 956.87	OOMYA2121-10	HQ708288, HQ643224
Phy	*Phytophthora erythroseptica*	P0340	PHYTO003-10	HQ261302, HQ261555
Phy	*Phytophthora erythroseptica*	p50	OOMYA2140-10	GU945475, GU993891
Phy	*Phytophthora erythroseptica*	P10382	PHYTO046-10	HQ261301, HQ261554, EU080780
Phy	*Phytophthora erythroseptica**	CBS 129.23	OOMYA013-07	HQ708286, HQ643221, HQ665121
Phy	*Phytophthora europaea*	BR 1072	OOMYA1302-08	HQ708293, HQ643229
Phy	*Phytophthora europaea*	P10324	PHYTO031-10	HQ261303, HQ261556, EU079486
Phy	*Phytophthora fallax*	P10722	PHYTO074-10	HQ261306, HQ261559
Phy	*Phytophthora fallax*	P10723	PHYTO075-10	HQ261305, HQ261558, EU080033
Phy	*Phytophthora fallax*	P10725	PHYTO076-10	HQ261304, HQ261557, EU080039
Phy	*Phytophthora foliorum*	P10969	PHYTO081-10	HQ261308, HQ261561, EU079684
Phy	*Phytophthora foliorum*	P10971	PHYTO082-10	HQ261307, HQ261560, EU079704
Phy	*Phytophthora fragariae*	CBS 309.62	OOMYA2095-10	HQ708295, HQ643231
Phy	*Phytophthora fragariae*	P11808	PHYTO089-10	HQ261312, HQ261565
Phy	*Phytophthora fragariae*	P6406	PHYTO196-10	HQ261309, HQ261562
Phy	*Phytophthora fragariae*	CBS 209.46	OOMYA044-07	HQ708294, HQ643230, HQ665150
Phy	*Phytophthora fragariae*	P1435	PHYTO101-10	HQ261311, HQ261564, EU079748
Phy	*Phytophthora fragariae*	P3820	PHYTO175-10	HQ261310, HQ261563, EU079839
Phy	*Phytophthora frigida*	P16051	PHYTO119-10	HQ261316, HQ261569
Phy	*Phytophthora frigida*	P16053	PHYTO121-10	HQ261315, HQ261568
Phy	*Phytophthora frigida*	P16054	PHYTO122-10	HQ261314, HQ261567
Phy	*Phytophthora frigida*	P16059	PHYTO123-10	HQ261313, HQ261566
Phy	*Phytophthora gonapodyides*	CBS 113346	OOMYA2040-10	HQ708299, HQ643236
Phy	*Phytophthora gonapodyides*	CBS 117380	OOMYA2063-10	HQ708296, HQ643232
Phy	*Phytophthora gonapodyides*	IMI 345174	OOMYA2160-10	GU945477, GU993893
Phy	*Phytophthora gonapodyides*	P7050	PHYTO213-10	HQ261317, HQ261570
Phy	*Phytophthora gonapodyides*	CBS 363.79	OOMYA134-07	HQ708298, HQ643235, HQ665216
Phy	*Phytophthora gonapodyides*	CBS 554.67	OOMYA187-07	HQ708297, HQ643233, HQ665265
Phy	*Phytophthora hedraiandra*	CBS 118732	OOMYA2066-10	HQ708300, HQ643237
Phy	*Phytophthora hedraiandra*	P11678	PHYTO088-10	HQ261318, HQ261571
Phy	*Phytophthora heveae*	P1000	PHYTO011-10	HQ261321, HQ261574
Phy	*Phytophthora heveae*	p28	OOMYA2133-10	GU945478, GU993894
Phy	*Phytophthora heveae*	P3428	PHYTO170-10	HQ261320, HQ261573
Phy	*Phytophthora heveae*	P8240	PHYTO240-10	HQ261319, HQ261572
Phy	*Phytophthora heveae*	P0578	PHYTO005-10	HQ261322, HQ261575, EU080701

Phy	*Phytophthora heveae**	CBS 296.29	OOMYA111-07	HQ708301, HQ643238, HQ665194
Phy	*Phytophthora hibernalis*	CBS 119904	OOMYA2068-10	HQ708302, HQ643240
Phy	*Phytophthora hibernalis*	CBS 522.77	OOMYA176-07	HQ708303, HQ643241
Phy	*Phytophthora hibernalis*	P3822	PHYTO176-10	HQ261323, HQ261576, EU079518
Phy	*Phytophthora himalayensis**	CBS 357.59	OOMYA132-07	HQ708304, HQ643242, HQ665215
Phy	*Phytophthora humicola*	CBS 114082	OOMYA2043-10	HQ708307, HQ643245
Phy	*Phytophthora humicola*	CBS 114083	OOMYA2044-10	HQ708306, HQ643244
Phy	*Phytophthora humicola*	P3826	PHYTO177-10	HQ261325, HQ261578, EU080173
Phy	*Phytophthora humicola*	P6701	PHYTO200-10	HQ261324, HQ261577, EU079923
Phy	*Phytophthora humicola**	CBS 200.81	OOMYA038-07	HQ708305, HQ643243, HQ665148
Phy	*Phytophthora idaei*	CBS 968.95	OOMYA2123-10	HQ708308, HQ643246
Phy	*Phytophthora idaei*	P6767	PHYTO202-10	HQ261326, HQ261579, EU080134
Phy	*Phytophthora ilicis*	P6098	PHYTO184-10	HQ261329, HQ261582
Phy	*Phytophthora ilicis*	P6099	PHYTO185-10	HQ261328, HQ261581
Phy	*Phytophthora ilicis*	P3939	PHYTO180-10	HQ261330, HQ261583, EU079864
Phy	*Phytophthora ilicis*	P6860	PHYTO204-10	HQ261327, HQ261580, EU080140
Phy	*Phytophthora infestans*	P12022	PHYTO091-10	HQ261335, HQ261588
Phy	*Phytophthora infestans*	P13198	PHYTO098-10	HQ261334, HQ261587
Phy	*Phytophthora infestans*	P15168	PHYTO110-10	HQ261333, HQ261586
Phy	*Phytophthora infestans*	P15938	PHYTO114-10	HQ261332, HQ261585
Phy	*Phytophthora infestans*	P15941	PHYTO115-10	HQ261331, HQ261584
Phy	*Phytophthora infestans*	CBS 366.51	OOMYA135-07	HQ708309, HQ643247, HQ665217
Phy	*Phytophthora infestans*	P10650	PHYTO062-10	HQ261336, HQ261589, EU079630
Phy	*Phytophthora inflata*	p122	OOMYA2130-10	GU945481, GU993896
Phy	*Phytophthora insolita*	IMI 288805	OOMYA2157-10	GU945482, GU993897
Phy	*Phytophthora insolita*	P6195	PHYTO187-10	HQ261338, HQ261591, EU080180
Phy	*Phytophthora insolita*	P6703	PHYTO201-10	HQ261337, HQ261590, EU080214
Phy	*Phytophthora inundata*	BR 332	OOMYA1316-08	HQ708312, HQ643252
Phy	*Phytophthora inundata*	CBS 216.85	OOMYA2077-10	HQ708310, HQ643250
Phy	*Phytophthora inundata*	P8479	PHYTO246-10	HQ261340, HQ261593
Phy	*Phytophthora inundata*	CBS 215.85	OOMYA1324-08	HQ708311, HQ643251, HQ665154
Phy	*Phytophthora inundata*	P8478	PHYTO245-10	HQ261341, HQ261594, EU079946
Phy	*Phytophthora inundata*	P8619	PHYTO251-10	HQ261339, HQ261592, EU080207
Phy	*Phytophthora ipomoeae*	PRI 810	OOMYA2153-10	HQ708313, HQ643253
Phy	*Phytophthora ipomoeae*	P10225	PHYTO022-10	HQ261344, HQ261597, EU080835
Phy	*Phytophthora ipomoeae*	P10226	PHYTO023-10	HQ261343, HQ261596, EU080842
Phy	*Phytophthora ipomoeae*	P10227	PHYTO024-10	HQ261342, HQ261595, EU080849
Phy	*Phytophthora iranica*	P3882	PHYTO179-10	HQ261345, HQ261598, EU080116
Phy	*Phytophthora iranica**	CBS 374.72	OOMYA138-07	HQ708314, HQ643254, HQ665219
Phy	*Phytophthora katsurae*	p45	OOMYA2139-10	GU945485, GU993899
Phy	*Phytophthora katsurae*	P6921	PHYTO208-10	HQ261346, HQ261599
Phy	*Phytophthora katsurae*	CBS 587.85	OOMYA201-07	HQ708315, HQ643255, HQ665278
Phy	*Phytophthora katsurae*	P10187	PHYTO019-10	HQ261348, HQ261601, EU080807
Phy	*Phytophthora katsurae*	P3389	PHYTO168-10	HQ261347, HQ261600, EU079819
Phy	*Phytophthora kernoviae*	IMI 393172	OOMYA2164-10	GU945486, GU993900
Phy	*Phytophthora kernoviae*	PRI 712	OOMYA2149-10	HQ708319, HQ643261
Phy	*Phytophthora kernoviae*	PRI 713	OOMYA2150-10	HQ708318, HQ643260
Phy	*Phytophthora kernoviae*	PRI 714	OOMYA2151-10	HQ708317, HQ643259
Phy	*Phytophthora kernoviae*	PRI 715	OOMYA2152-10	HQ708316, HQ643258
Phy	*Phytophthora kernoviae*	P10671	PHYTO064-10	HQ261351, HQ261604, EU080032
Phy	*Phytophthora kernoviae*	P10681	PHYTO066-10	HQ261350, HQ261603, EU079650
Phy	*Phytophthora kernoviae*	P10958	PHYTO080-10	HQ261349, HQ261602, EU080057
Phy	*Phytophthora lateralis*	Lev 1213	OOMYA249-07	HQ708320, HQ643263
Phy	*Phytophthora lateralis*	p51	OOMYA2141-10	GU945487, GU993901
Phy	*Phytophthora litchii*	BR 892	OOMYA1239-08	HQ708322, HQ643265
Phy	*Phytophthora litchii*	BR 893	OOMYA1240-08	HQ708321, HQ643264
Phy	*Phytophthora litchii*	CBS 100.81	OOMYA002-07	HQ708323, HQ643266
Phy	*Phytophthora macrochlamydospora*	IMI 351473	OOMYA2161-10	GU945488, GU993902
Phy	*Phytophthora macrochlamydospora*	P10267	PHYTO026-10	HQ261353, HQ261606, EU080009
Phy	*Phytophthora macrochlamydospora*	P8017	PHYTO222-10	HQ261352, HQ261605, EU080662
Phy	*Phytophthora meadii*	p75	OOMYA2143-10	GU945489, GU993903
Phy	*Phytophthora meadii*	CBS 219.88	OOMYA055-07	HQ708324, HQ643268, HQ665159
Phy	*Phytophthora meadii*	P6128	PHYTO186-10	HQ261354, HQ261607, EU079878
Phy	*Phytophthora medicaginis*	BR 610	OOMYA1583-08	HQ708328, HQ643273
Phy	*Phytophthora medicaginis*	BR 611	OOMYA1584-08	HQ708327, HQ643272
Phy	*Phytophthora medicaginis*	BR 622	OOMYA1265-08	HQ708326, HQ643271
Phy	*Phytophthora medicaginis*	CBS 117685	OOMYA2064-10	HQ708325, HQ643270

Phy	*Phytophthora medicaginis*	P10127	PHYTO014-10	HQ261355, HQ261608
Phy	*Phytophthora megakarya*	P1664	PHYTO133-10	HQ261358, HQ261611
Phy	*Phytophthora megakarya*	P1672	PHYTO134-10	HQ261357, HQ261610
Phy	*Phytophthora megakarya*	p42	OOMYA2136-10	GU945490, GU993904
Phy	*Phytophthora megakarya*	P8516	PHYTO250-10	HQ261356, HQ261609, EU079974
Phy	*Phytophthora megasperma*	BR 395	OOMYA1296-08	HQ708335, HQ643282
Phy	*Phytophthora megasperma*	BR 396	OOMYA1297-08	HQ708334, HQ643281
Phy	*Phytophthora megasperma*	BR 398	OOMYA1299-08	HQ708333, HQ643280
Phy	*Phytophthora megasperma*	BR 528	OOMYA413-07	HQ708332, HQ643279
Phy	*Phytophthora megasperma*	BR 529	OOMYA1300-08	HQ708331, HQ643278
Phy	*Phytophthora megasperma*	Ip81	OOMYA2129-10	GU945491, GU993905
Phy	*Phytophthora megasperma*	P6957	PHYTO212-10	HQ261359, HQ261612
Phy	*Phytophthora megasperma*	CBS 306.36	OOMYA116-07	HQ708330, HQ643277, HQ665201
Phy	*Phytophthora megasperma*	P10340	PHYTO040-10	HQ261362, HQ261615, EU080383
Phy	*Phytophthora megasperma*	P1679	PHYTO135-10	HQ261361, HQ261614, EU080337
Phy	*Phytophthora megasperma*	P3136	PHYTO159-10	HQ261360, HQ261613, EU080063
Phy	*Phytophthora megasperma**	CBS 402.72	OOMYA150-07	HQ708329, HQ643275, HQ665228
Phy	*Phytophthora melonis*	IMI 325917	OOMYA2158-10	GU945492, GU993906
Phy	*Phytophthora melonis*	P6870	PHYTO205-10	HQ261363, HQ261616
Phy	*Phytophthora melonis*	P3609	PHYTO173-10	HQ261364, HQ261617, EU080476
Phy	*Phytophthora melonis**	CBS 582.69	OOMYA198-07	HQ708336, HQ643283, HQ665274
Phy	*Phytophthora mengei*	P10139	PHYTO015-10	HQ261366, HQ261619
Phy	*Phytophthora mengei*	P1275	PHYTO096-10	HQ261365, HQ261618
Phy	*Phytophthora mexicana*	P0646	PHYTO007-10	HQ261367, HQ261620, EU080707
Phy	*Phytophthora mirabilis*	p3006	OOMYA2134-10	HQ708338
Phy	*Phytophthora mirabilis*	CBS 122204	OOMYA2069-10	HQ708337, HQ643285
Phy	*Phytophthora mirabilis*	P10231	PHYTO025-10	HQ261370, HQ261623
Phy	*Phytophthora mirabilis*	P3010	PHYTO154-10	HQ261368, HQ261621
Phy	*Phytophthora mirabilis*	P3005	PHYTO153-10	HQ261369, HQ261622, EU079780
Phy	*Phytophthora mirabilis*	p3007	OOMYA2135-10	GU993907
Phy	*Phytophthora mirabilis**	CBS 678.85	OOMYA211-07	HQ708339, HQ643287, HQ665285
Phy	*Phytophthora multivesiculata*	IMI 386053	OOMYA2128-10	GU945493, GU993908
Phy	*Phytophthora multivesiculata*	P10670	PHYTO063-10	HQ261371, HQ261624
Phy	*Phytophthora multivesiculata**	CBS 545.96	OOMYA180-07	HQ708340, HQ643288, HQ665257
Phy	*Phytophthora multivora*	BR 514	OOMYA1244-08	HQ708343, HQ643292
Phy	*Phytophthora multivora*	CBS 111337	OOMYA2028-10	HQ708344, HQ643293
Phy	*Phytophthora multivora*	CBS 113347	OOMYA2041-10	HQ708342, HQ643291
Phy	*Phytophthora multivora*	CBS 119108	OOMYA2067-10	HQ708341, HQ643290
Phy	*Phytophthora multivora*	P1233	PHYTO093-10	HQ261373, HQ261626
Phy	*Phytophthora multivora*	P7902	PHYTO221-10	HQ261372, HQ261625, EU080240
Phy	*Phytophthora nemorosa*	P16352	PHYTO130-10	HQ261374, HQ261627
Phy	*Phytophthora nemorosa*	PRI 708	OOMYA2145-10	HQ708349, HQ643298
Phy	*Phytophthora nemorosa*	PRI 709	OOMYA2146-10	HQ708348, HQ643297
Phy	*Phytophthora nemorosa*	PRI 710	OOMYA2147-10	HQ708347, HQ643296
Phy	*Phytophthora nemorosa*	PRI 711	OOMYA2148-10	HQ708346, HQ643295
Phy	*Phytophthora nemorosa*	P10288	PHYTO028-10	HQ261375, HQ261628, EU079479
Phy	*Phytophthora nemorosa**	CBS 114870	OOMYA2057-10	HQ708345, HQ643294
Phy	*Phytophthora nicotianae*	81	OOMYA2011-10	GU945494, GU993910
Phy	*Phytophthora nicotianae*	CBS 101655	OOMYA2017-10	HQ708354, HQ643303
Phy	*Phytophthora nicotianae*	CBS 303.29	OOMYA2091-10	HQ708352, HQ643301
Phy	*Phytophthora nicotianae*	CBS 304.29	OOMYA2092-10	HQ708351, HQ643300
Phy	*Phytophthora nicotianae*	CBS 310.62	OOMYA2096-10	HQ708353, HQ643302
Phy	*Phytophthora nicotianae*	CBS 534.92	OOMYA2111-10	HQ708350, HQ643299
Phy	*Phytophthora nicotianae*	P10297	PHYTO029-10	HQ261379, HQ261632
Phy	*Phytophthora nicotianae*	P10381	PHYTO045-10	HQ261378, HQ261631
Phy	*Phytophthora nicotianae*	P6915	PHYTO207-10	HQ261377, HQ261630
Phy	*Phytophthora nicotianae*	P7146	PHYTO215-10	HQ261376, HQ261629, EU079560
Phy	*Phytophthora palmivora*	CBS 179.26	OOMYA2073-10	HQ708355, HQ643305
Phy	*Phytophthora palmivora*	CBS 274.33	OOMYA2086-10	HQ708358, HQ643308
Phy	*Phytophthora palmivora*	CBS 299.29	OOMYA2090-10	HQ708356, HQ643306
Phy	*Phytophthora palmivora*	P16385	PHYTO132-10	HQ261381, HQ261634
Phy	*Phytophthora palmivora*	P6390	PHYTO195-10	HQ261380, HQ261633
Phy	*Phytophthora palmivora*	CBS 298.29	OOMYA113-07	HQ708357, HQ643307, HQ665195
Phy	*Phytophthora palmivora*	P0113	PHYTO001-10	HQ261383, HQ261636, EU080469
Phy	*Phytophthora palmivora*	P0255	PHYTO002-10	HQ261382, HQ261635, EU080343
Phy	*Phytophthora parsiana*	P15164	PHYTO109-10	HQ261386, HQ261639
Phy	*Phytophthora parsiana*	P21281	PHYTO149-10	HQ261385, HQ261638

Phy	*Phytophthora parsiana*	P21282	PHYTO150-10	HQ261384, HQ261637
Phy	*Phytophthora phaseoli*	CBS 556.88	OOMYA189-07	HQ708359, HQ643309, HQ665267
Phy	*Phytophthora phaseoli*	P10145	PHYTO016-10	HQ261389, HQ261642, EU080753
Phy	*Phytophthora phaseoli*	P10150	PHYTO017-10	HQ261388, HQ261641, EU080766
Phy	*Phytophthora phaseoli*	P6609	PHYTO199-10	HQ261387, HQ261640, EU079918
Phy	*Phytophthora pini**	CBS 181.25	OOMYA035-07	HQ708360, HQ643310, HQ665145
Phy	*Phytophthora pinifolia*	P16100	PHYTO124-10	HQ261390, HQ261643
Phy	*Phytophthora pistaciae*	P6196	PHYTO188-10	HQ261392, HQ261645, EU080323
Phy	*Phytophthora pistaciae*	P6197	PHYTO189-10	HQ261391, HQ261644, EU080329
Phy	*Phytophthora plurivora*	CBS 117378	OOMYA2062-10	HQ708361, HQ643311
Phy	*Phytophthora plurivora*	CBS 379.61	OOMYA2104-10	HQ708362, HQ643312
Phy	*Phytophthora polonica*	P15004	PHYTO104-10	HQ261394, HQ261647, EU080268
Phy	*Phytophthora polonica*	P15005	PHYTO105-10	HQ261393, HQ261646, EU080261
Phy	*Phytophthora polymorphica**	CBS 680.84	OOMYA214-07	HQ708363, HQ643313, HQ665288
Phy	*Phytophthora porri*	CBS 114100	OOMYA2051-10	HQ708370, HQ643320
Phy	*Phytophthora porri*	CBS 116662	OOMYA2059-10	HQ708367, HQ643317
Phy	*Phytophthora porri*	CBS 141.87	OOMYA2070-10	HQ708366, HQ643316
Phy	*Phytophthora porri*	CBS 142.87	OOMYA2071-10	HQ708365, HQ643315
Phy	*Phytophthora porri*	CBS 181.87	OOMYA2075-10	HQ708364, HQ643314
Phy	*Phytophthora porri*	CBS 783.97	OOMYA2116-10	HQ708369, HQ643319
Phy	*Phytophthora porri*	P7518	PHYTO219-10	HQ261396, HQ261649
Phy	*Phytophthora porri*	P7899	PHYTO220-10	HQ261395, HQ261648
Phy	*Phytophthora porri*	CBS 567.86	OOMYA191-07	HQ708368, HQ643318, HQ665271
Phy	*Phytophthora primulae*	CBS 100531	OOMYA2016-10	HQ708372, HQ643322
Phy	*Phytophthora primulae*	CBS 110162	OOMYA2021-10	HQ708374, HQ643324
Phy	*Phytophthora primulae*	CBS 110167	OOMYA2022-10	HQ708373, HQ643323
Phy	*Phytophthora primulae*	CBS 620.97	OOMYA2113-10	HQ708371, HQ643321
Phy	*Phytophthora primulae*	P10220	PHYTO021-10	HQ261398, HQ261651, EU080821
Phy	*Phytophthora primulae*	P10333	PHYTO036-10	HQ261397, HQ261650, EU080403
Phy	*Phytophthora pseudosyringae*	CBS 111773	OOMYA2034-10	HQ708378, HQ643329
Phy	*Phytophthora pseudosyringae*	CBS 111774	OOMYA2035-10	HQ708377, HQ643328
Phy	*Phytophthora pseudosyringae*	CBS 111775	OOMYA2036-10	HQ708376, HQ643327
Phy	*Phytophthora pseudosyringae*	CBS 114108	OOMYA2053-10	HQ708375, HQ643326
Phy	*Phytophthora pseudosyringae*	IMI 391716	OOMYA2162-10	GU945496, GU993912
Phy	*Phytophthora pseudosyringae*	P16355	PHYTO131-10	HQ261399, HQ261652
Phy	*Phytophthora pseudosyringae*	P10443	PHYTO052-10	HQ261400, HQ261653, EU080026
Phy	*Phytophthora pseudotsugae*	CBS 445.84	OOMYA2109-10	HQ708380, HQ643331
Phy	*Phytophthora pseudotsugae*	CBS 446.84	OOMYA2110-10	HQ708379, HQ643330
Phy	*Phytophthora pseudotsugae*	IMI 331663	OOMYA2159-10	GU945497, GU993913
Phy	*Phytophthora pseudotsugae*	CBS 444.84	OOMYA160-07	HQ708381, HQ643332, HQ665234
Phy	*Phytophthora pseudotsugae*	P10218	PHYTO020-10	HQ261402, HQ261655, EU079992
Phy	*Phytophthora pseudotsugae*	P10339	PHYTO039-10	HQ261401, HQ261654, EU080431
Phy	*Phytophthora psychrophila*	P10433	PHYTO049-10	HQ261403, HQ261656, EU080521
Phy	*Phytophthora quercetorum*	P15555	PHYTO111-10	HQ261404, HQ261657
Phy	*Phytophthora quercina*	CBS 781.95	OOMYA2114-10	HQ708385, HQ643337
Phy	*Phytophthora quercina*	CBS 786.95	OOMYA2117-10	HQ708384, HQ643336
Phy	*Phytophthora quercina*	CBS 787.95	OOMYA2118-10	HQ708383, HQ643335
Phy	*Phytophthora quercina*	CBS 788.95	OOMYA2119-10	HQ708382, HQ643334
Phy	*Phytophthora quercina*	P10334	PHYTO037-10	HQ261406, HQ261659, EU080494
Phy	*Phytophthora quercina*	P10441	PHYTO051-10	HQ261405, HQ261658, EU080596
Phy	*Phytophthora quininea*	P8488	PHYTO247-10	HQ261407, HQ261660
Phy	*Phytophthora quininea*	P3247	PHYTO163-10	HQ261408, HQ261661, EU079806
Phy	*Phytophthora quininea**	CBS 407.48	OOMYA153-07	HQ708386, HQ643338, HQ665230
Phy	*Phytophthora ramorum*	P10102	PHYTO012-10	HQ261410, HQ261663, EU080734
Phy	*Phytophthora ramorum*	P10301	PHYTO030-10	HQ261409, HQ261662, EU080688
Phy	*Phytophthora ramorum**	CBS 101553	OOMYA246-07	HQ708387, HQ643339, HQ665053
Phy	*Phytophthora rosacearum*	P3159	PHYTO160-10	HQ261411, HQ261664
Phy	*Phytophthora rubi*	CBS 109892	OOMYA2018-10	HQ708389, HQ643341
Phy	*Phytophthora rubi*	P3289	PHYTO165-10	HQ261413, HQ261666
Phy	*Phytophthora rubi*	P3316	PHYTO166-10	HQ261412, HQ261665
Phy	*Phytophthora rubi**	CBS 967.95	OOMYA244-07	HQ708388, HQ643340, HQ665306
Phy	*Phytophthora sansomeana*	BR 1058	OOMYA1318-08	HQ708391, HQ643343
Phy	*Phytophthora sansomeana*	BR 623	OOMYA1295-08	HQ708390, HQ643342
Phy	*Phytophthora sansomeana*	CBS 957.87	OOMYA2122-10	HQ708215, HQ643141
Phy	*Phytophthora sansomeana*	P8048	PHYTO223-10	HQ261415, HQ261668
Phy	*Phytophthora sansomeana*	P8049	PHYTO224-10	HQ261414, HQ261667
Phy	*Phytophthora sansomeana*	P3163	PHYTO161-10	HQ261416, HQ261669, EU080275

Phy	*Phytophthora sinensis*	P1748	PHYTO138-10	HQ261417, HQ261670
Phy	*Phytophthora sinensis*	P1475	PHYTO103-10	HQ261418, HQ261671, EU079754
Phy	*Phytophthora sinensis**	CBS 557.88	OOMYA190-07	HQ708392, HQ643344, HQ665269
Phy	*Phytophthora siskiyouensis*	P16301	PHYTO128-10	HQ261419, HQ261672
Phy	*Phytophthora siskiyouensis*	PRI 816	OOMYA2155-10	HQ708394, HQ643346
Phy	*Phytophthora siskiyouensis*	PRI 817	OOMYA2156-10	HQ708393, HQ643345
Phy	*Phytophthora siskiyouensis*	P15122	PHYTO106-10	HQ261421, HQ261674, HQ665311
Phy	*Phytophthora siskiyouensis*	P15123	PHYTO107-10	HQ261420, HQ261673, HQ665312
Phy	*Phytophthora sojae*	BR 1079	OOMYA1311-08	HQ708397, HQ643349
Phy	*Phytophthora sojae*	BR 582	OOMYA1308-08	HQ708396, HQ643348
Phy	*Phytophthora sojae*	P0405	PHYTO004-10	HQ261426, HQ261679
Phy	*Phytophthora sojae*	P10704	PHYTO069-10	HQ261425, HQ261678
Phy	*Phytophthora sojae*	P6497	PHYTO197-10	HQ261423, HQ261676
Phy	*Phytophthora sojae*	P7061	PHYTO214-10	HQ261422, HQ261675
Phy	*Phytophthora sojae*	CBS 382.61	OOMYA146-07	HQ708395, HQ643347, HQ665224
Phy	*Phytophthora sojae*	P3114	PHYTO158-10	HQ261424, HQ261677, EU079794
Phy	*Phytophthora* sp.	P6875	PHYTO206-10	HQ261427, HQ261680, EU080548
Phy	*Phytophthora* sp. *"andina"*	EC 3163	OOMYA2125-10	GU945464, FJ769180
Phy	*Phytophthora* sp. *"andina"*	PRI 814	OOMYA2154-10	HQ708398, HQ643350
Phy	*Phytophthora* sp. *"asparagi"*	P10693	PHYTO068-10	HQ261429, HQ261682
Phy	*Phytophthora* sp. *"asparagi"*	P10707	PHYTO071-10	HQ261428, HQ261681
Phy	*Phytophthora* sp. *"asparagi"*	P10690	PHYTO067-10	HQ261430, HQ261683, EU080569
Phy	*Phytophthora* sp. *"canalensis"*	P10456	PHYTO053-10	HQ261431, HQ261684, EU079574
Phy	*Phytophthora* sp. *"cuyabensis"*	P8230	PHYTO236-10	HQ261433, HQ261686
Phy	*Phytophthora* sp. *"cuyabensis"*	P8232	PHYTO237-10	HQ261432, HQ261685
Phy	*Phytophthora* sp. *"cuyabensis"*	P8213	PHYTO230-10	HQ261435, HQ261688, EU080669
Phy	*Phytophthora* sp. *"cuyabensis"*	P8218	PHYTO231-10	HQ261434, HQ261687, EU080356
Phy	*Phytophthora* sp. *"glovera"*	P10618	PHYTO060-10	HQ261437, HQ261690, EU080220
Phy	*Phytophthora* sp. *"glovera"*	P10619	PHYTO061-10	HQ261436, HQ261689, EU080227
Phy	*Phytophthora* sp. *"kelmania"*	CBS 307.62	OOMYA2094-10	HQ708399, HQ643351
Phy	*Phytophthora* sp. *"kelmania"*	P10614	PHYTO057-10	HQ261438, HQ261691
Phy	*Phytophthora* sp. *"kelmania"*	P10613	PHYTO056-10	HQ261439, HQ261692, EU079610
Phy	*Phytophthora* sp. *"lacrimae"*	P15880	PHYTO113-10	HQ261440, HQ261693
Phy	*Phytophthora* sp. *"lagoariana"*	P8220	PHYTO232-10	HQ261442, HQ261695, EU080362
Phy	*Phytophthora* sp. *"lagoariana"*	P8223	PHYTO234-10	HQ261441, HQ261694, EU080369
Phy	*Phytophthora* sp. *"napoensis"*	P8221	PHYTO233-10	HQ261445, HQ261698
Phy	*Phytophthora* sp. *"napoensis"*	P8225	PHYTO235-10	HQ261444, HQ261697
Phy	*Phytophthora* sp. *"napoensis"*	P8233	PHYTO238-10	HQ261443, HQ261696
Phy	*Phytophthora* sp. *"niederhauserii"*	P10279	PHYTO027-10	HQ261450, HQ261703
Phy	*Phytophthora* sp. *"niederhauserii"*	P10976	PHYTO083-10	HQ261447, HQ261700
Phy	*Phytophthora* sp. *"niederhauserii"*	P16237	PHYTO127-10	HQ261446, HQ261699
Phy	*Phytophthora* sp. *"niederhauserii"*	P10616	PHYTO058-10	HQ261449, HQ261702, EU080233
Phy	*Phytophthora* sp. *"niederhauserii"*	P10617	PHYTO059-10	HQ261448, HQ261701, EU080247
Phy	*Phytophthora* sp. *"novaeguinea"*	P1256	PHYTO094-10	HQ261456, HQ261709
Phy	*Phytophthora* sp. *"ohioensis"*	P16050	PHYTO118-10	HQ261457, HQ261710
Phy	*Phytophthora* sp. *"sulawesiensis"*	P6306	PHYTO193-10	HQ261458, HQ261711, EU080349
Phy	*Phytophthora* sp. *"thermophilum"*	P1896	PHYTO141-10	HQ261459, HQ261712
Phy	*Phytophthora* sp. *"thermophilum"*	P10457	PHYTO054-10	HQ261460, HQ261713, EU079580
Phy	*Phytophthora* sp. nov.	BR 333	OOMYA1243-08	HQ708403, HQ643355
Phy	*Phytophthora* sp. nov.	CBS 111346	OOMYA2032-10	HQ708402, HQ643354
Phy	*Phytophthora* sp. nov.	CBS 114338	OOMYA2056-10	HQ708400, HQ643352
Phy	*Phytophthora* sp. nov.	CBS 368.79	OOMYA2102-10	HQ708401, HQ643353
Phy	*Phytophthora* sp. nov.	P1212	PHYTO092-10	HQ261453, HQ261706
Phy	*Phytophthora* sp. nov.	P3600	PHYTO171-10	HQ261452, HQ261705
Phy	*Phytophthora* sp. nov.	P10337	PHYTO038-10	HQ261455, HQ261708, EU080535
Phy	*Phytophthora* sp. nov.	P10728	PHYTO077-10	HQ261454, HQ261707, EU079677
Phy	*Phytophthora* sp. nov.	P6207	PHYTO190-10	HQ261451, HQ261704, EU079885
Phy	*Phytophthora syringae*	CBS 110161	OOMYA2020-10	HQ708410, HQ643362
Phy	*Phytophthora syringae*	CBS 114107	OOMYA2052-10	HQ708409, HQ643361
Phy	*Phytophthora syringae*	CBS 114109	OOMYA2054-10	HQ708408, HQ643360
Phy	*Phytophthora syringae*	CBS 114110	OOMYA2055-10	HQ708407, HQ643359
Phy	*Phytophthora syringae*	CBS 275.74	OOMYA097-07	HQ708405, HQ643357
Phy	*Phytophthora syringae*	CBS 364.52	OOMYA2101-10	HQ708406, HQ643358
Phy	*Phytophthora syringae*	P2004	PHYTO146-10	HQ261461, HQ261714
Phy	*Phytophthora syringae*	CBS 132.23	OOMYA014-07	HQ708404, HQ643356, HQ665123
Phy	*Phytophthora syringae*	P10330	PHYTO033-10	HQ261463, HQ261716, EU080562
Phy	*Phytophthora syringae*	P10332	PHYTO035-10	HQ261462, HQ261715, EU079767

Phy	*Phytophthora tabaci**	CBS 305.29	OOMYA114-07	HQ708411, HQ643363, HQ665198
Phy	*Phytophthora tentaculata*	CBS 100411	OOMYA2015-10	HQ708415, HQ643367
Phy	*Phytophthora tentaculata*	CBS 115458	OOMYA2058-10	HQ708412, HQ643364
Phy	*Phytophthora tentaculata*	CBS 412.96	OOMYA2107-10	HQ708414, HQ643366
Phy	*Phytophthora tentaculata*	P10363	PHYTO042-10	HQ261465, HQ261718
Phy	*Phytophthora tentaculata*	CBS 552.96	OOMYA185-07	HQ708413, HQ643365, HQ665264
Phy	*Phytophthora tentaculata*	P8497	PHYTO249-10	HQ261464, HQ261717, EU079960
Phy	*Phytophthora trifolii*	CBS 117688	OOMYA2065-10	HQ708416, HQ643368
Phy	*Phytophthora trifolii*	P1462	PHYTO102-10	HQ261466, HQ261719, EU080088
Phy	*Phytophthora tropicalis*	p27	OOMYA2132-10	GU945500, GU993915
Phy	*Phytophthora tropicalis*	P6522	PHYTO198-10	HQ261467, HQ261720
Phy	*Phytophthora tropicalis**	CBS 434.91	OOMYA159-07	HQ708417, HQ643369, HQ665233
Phy	*Phytophthora uliginosa*	P10328	PHYTO032-10	HQ261469, HQ261722, EU079697
Phy	*Phytophthora uliginosa*	P10413	PHYTO048-10	HQ261468, HQ261721, EU080015
Phy	*Phytophthora vignae*	P7471	PHYTO218-10	HQ261470, HQ261723
Phy	*Phytophthora vignae*	P3019	PHYTO155-10	HQ261471, HQ261724, EU079787
	Phytopythium aff. *cucurbitacearum*	Lev 2210	OOMYA450-07	HQ708996, HQ643955
	Phytopythium aff. *vexans*	Lev 3100	OOMYA1375-08	HQ708995, HQ643954
	Phytopythium aff. *vexans*	CBS 261.30	OOMYA090-07	HQ708418, HQ643371, HQ665178
	Phytopythium boreale	CBS 551.88	OOMYA183-07	HQ708419, HQ643372, HQ665261
	*Phytopythium carbonicum**	CBS 112544	OOMYA1372-08	HQ708420, HQ643373, HQ665073
	*Phytopythium chamaehyphon**	CBS 259.30	OOMYA088-07	HQ708421, HQ643374, HQ665177
	Phytopythium citrinum	ADC 9442	OOMYA1649-08	HQ708427, HQ643380
	Phytopythium citrinum	ADC 9512	OOMYA1754-08	HQ708426, HQ643379
	Phytopythium citrinum	ADC 9513	OOMYA1755-08	HQ708425, HQ643378
	Phytopythium citrinum	ADC 9515	OOMYA1756-08	HQ708424, HQ643377
	Phytopythium citrinum	ADC 9519	OOMYA1758-08	HQ708423, HQ643376
	*Phytopythium citrinum**	CBS 119171	OOMYA1378-08	HQ708422, HQ643375, HQ665088
	Phytopythium cucurbitacearum	CBS 748.96	OOMYA230-07	HQ708428, HQ643381, HQ665292
	Phytopythium helicoides	ADC 9423	OOMYA1648-08	HQ708431, HQ643384
	Phytopythium helicoides	CBS 167.68	OOMYA1419-08	HQ708429, HQ643382
	*Phytopythium helicoides**	CBS 286.31	OOMYA1420-08	HQ708430, HQ643383, HQ665186
	Phytopythium litorale	BR 896	OOMYA1695-08	HQ708434, HQ643387
	Phytopythium litorale	Lev 3002	OOMYA452-07	HQ708721, HQ643677
	Phytopythium litorale	Lev 3006	OOMYA454-07	HQ708720, HQ643676
	Phytopythium litorale	Lev 3011	OOMYA1676-08	HQ708719, HQ643675
	Phytopythium litorale	Lev 3012	OOMYA1677-08	HQ708718, HQ643674
	Phytopythium litorale	Lev 3013	OOMYA1678-08	HQ708717, HQ643673
	Phytopythium litorale	CBS 122662	OOMYA1379-08	HQ708432, HQ643385, HQ665114
	*Phytopythium litorale**	CBS 118360	OOMYA1570-08	HQ708433, HQ643386, HQ665082
	*Phytopythium megacarpum**	CBS 112351	OOMYA1585-08	HQ708435, HQ643388, HQ665067
	Phytopythium montanum	ADC 9762	OOMYA1591-08	HQ708438, HQ643391
	Phytopythium montanum	ADC 9766	OOMYA1592-08	HQ708437, HQ643390
	*Phytopythium montanum**	CBS 111349	OOMYA1593-08	HQ708436, HQ643389, HQ665064
	Phytopythium oedochilum	DAOM 229148	OOMYA1560-08	HQ708756, HQ643712
	*Phytopythium oedochilum**	CBS 292.37	OOMYA107-07	HQ708439, HQ643392, HQ665191
	Phytopythium ostracodes	CBS 768.73	OOMYA233-07	HQ708442, HQ643395, HQ665295
	*Phytopythium sindhum**	DAOM 238986	OOMYA2165-10	HQ708443, HQ643396, HQ665309
	Phytopythium sp. *"amazonianum"*	P8239	PHYTO239-10	HQ261476, HQ261729
	Phytopythium sp. *"amazonianum"*	P8242	PHYTO241-10	HQ261475, HQ261728
	Phytopythium sp. *"amazonianum"*	P8243	PHYTO242-10	HQ261474, HQ261727
	Phytopythium sp. *"amazonianum"*	P8246	PHYTO243-10	HQ261473, HQ261726
	Phytopythium sp. *"amazonianum"*	P8247	PHYTO244-10	HQ261472, HQ261725
	Phytopythium sp. *"grandilobatum"*	CBS 738.94	OOMYA1415-08	HQ708441, HQ643394
	Phytopythium sp. *"grandilobatum"*	CBS 739.94	OOMYA1416-08	HQ708440, HQ643393
	Phytopythium sp. nov.	ADC 0004	OOMYA1753-08	HQ708446, HQ643399
	Phytopythium sp. nov.	ADC 9517	OOMYA1757-08	HQ708445, HQ643398
	Phytopythium sp. nov.	ADC 9520	OOMYA1650-08	HQ708444, HQ643397
	Phytopythium vexans	CBS 455.62	OOMYA164-07	HQ708448, HQ643401
	Phytopythium vexans	CBS 119.80	OOMYA011-07	HQ708447, HQ643400, HQ665090
	Phytopythium vexans	P3980	PHYTO183-10	HQ261477, HQ261730, EU080487
Pla	*Plasmopara euphrasiae*	HV 2226	DM016-10	HM033194, EF553509, EF553465
Pla	*Plasmopara nivea*	HV 75	DM012-10	HM033195, HM004513
Pla	*Plasmopara pusilla*	HV 140	DM013-10	HM033196, HM004512, DQ148402
	Plasmoverna anemones-ranunculoidis	HV 125	DM014-10	HM033198, HM004511, HM004514
	Plasmoverna anemones-ranunculoidis	HV 101		EF553471

Ple	*Plectospira myriandra*	CBS 523.87	OOMYA1328-08	HQ708449, HQ643402, HQ665247
Pro	*Protoachlya paradoxa*	CBS 100.44	OOMYA1189-08	HQ708451, HQ643404
Pro	*Protoachlya paradoxa*	CBS 158.45	OOMYA1329-08	HQ708450, HQ643403, HQ665135
Pse	*Pseudoperonospora cubensis*	HV 222	DM010-10	HM033199, AY198306
Pse	*Pseudoperonospora cubensis*	HV 221h		AY035496
	Pythiogeton zeae	Lev 3132	OOMYA2166-10	HQ708452, HQ643405, HQ665310
	Pythiopsis terrestris	CBS 110058	OOMYA1816-08	HQ708454, HQ643407
	Pythiopsis terrestris	CBS 110059	OOMYA1817-08	HQ708453, HQ643406
Pyt	*Pythium abappressorium**	CBS 110198	OOMYA1331-08	HQ708455, HQ643408, HQ665063
Pyt	*Pythium acanthicum*	BR 228	OOMYA1332-08	HQ708458, HQ643411
Pyt	*Pythium acanthicum*	BR 500	OOMYA556-08	HQ708459, HQ643412
Pyt	*Pythium acanthicum*	Lev 1618	OOMYA342-07	HQ708457, HQ643410
Pyt	*Pythium acanthicum*	CBS 377.34	OOMYA141-07	HQ708456, HQ643409, HQ665222
Pyt	*Pythium acanthophoron**	CBS 337.29	OOMYA128-07	HQ708460, HQ643413, HQ665212
Pyt	*Pythium acrogynum**	CBS 549.88	OOMYA181-07	HQ708461, HQ643414, HQ665258
Pyt	*Pythium adhaerens*	CBS 520.74	OOMYA174-07	HQ708462, HQ643415, HQ665245
Pyt	*Pythium afertile*	Lev 2066	OOMYA1334-08	HQ708463, HQ643416
Pyt	*Pythium* aff. *attrantheridium*	Lev 3004	OOMYA1669-08	HQ708520, HQ643473
Pyt	*Pythium* aff. *diclinum*	CBS 229.94	OOMYA1336-08	HQ708464, HQ643417
Pyt	*Pythium* aff. *dictyosporum*	ADC 0113	OOMYA1662-08	HQ708466, HQ643419
Pyt	*Pythium* aff. *dictyosporum*	ADC 0116	OOMYA1664-08	HQ708465, HQ643418
Pyt	*Pythium* aff. *dissotocum*	BR 725	OOMYA1665-08	HQ708470, HQ643423
Pyt	*Pythium* aff. *dissotocum*	BR 726	OOMYA1666-08	HQ708469, HQ643422
Pyt	*Pythium* aff. *dissotocum*	BR 907	OOMYA1345-08	HQ708467, HQ643420
Pyt	*Pythium* aff. *dissotocum*	BR 992	OOMYA1821-08	HQ708468, HQ643421
Pyt	*Pythium* aff. *hydnosporum*	BR 978	OOMYA1697-08	HQ708471, HQ643424
Pyt	*Pythium* aff. *hypogynum*	BR 116	OOMYA1636-08	HQ708472, HQ643425
Pyt	*Pythium* aff. *iwayamai*	DAOM 229147	OOMYA1452-08	HQ708827, HQ643786
Pyt	*Pythium* aff. *macrosporum*	BR 1029	OOMYA1737-08	HQ708729, HQ643685
Pyt	*Pythium* aff. *oopapillum*	Lev 1619	OOMYA343-07	HQ708761, HQ643717
Pyt	*Pythium* aff. *periilum*	CBS 237.83	OOMYA1626-08	HQ708473, HQ643426
Pyt	*Pythium* aff. *perplexum*	BR 925	OOMYA1686-08	HQ708474, HQ643427
Pyt	*Pythium* aff. *pleroticum*	ADC 9912	OOMYA1779-08	HQ708475, HQ643428
Pyt	*Pythium* aff. *pleroticum*	Lev 2114	OOMYA423-07	HQ708788, HQ643747
Pyt	*Pythium* aff. *polymastum*	ADC 9817	OOMYA1652-08	HQ708476, HQ643429
Pyt	*Pythium* aff. *polymastum*	Lev 1630	OOMYA350-07	HQ708792, HQ643751
Pyt	*Pythium* aff. *torulosum*	BR 626	OOMYA349-07	HQ708479, HQ643432
Pyt	*Pythium* aff. *torulosum*	BR 780	OOMYA1683-08	HQ708478, HQ643431
Pyt	*Pythium* aff. *torulosum*	BR 897	OOMYA1641-08	HQ708477, HQ643430
Pyt	*Pythium* aff. *volutum*	BR 974	OOMYA1355-08	HQ708480, HQ643433
Pyt	*Pythium* aff. *volutum*	Lev 1528	OOMYA1594-08	HQ709011, HQ643970
Pyt	*Pythium amasculinum**	CBS 552.88	OOMYA184-07	HQ708481, HQ643434, HQ665263
Pyt	*Pythium anandrum*	Lev 2116	OOMYA424-07	HQ708483, HQ643436
Pyt	*Pythium anandrum**	CBS 285.31	OOMYA1335-08	HQ708482, HQ643435, HQ665185
Pyt	*Pythium angustatum*	CBS 522.74	OOMYA175-07	HQ708484, HQ643437, HQ665246
Pyt	*Pythium aphanidermatum*	CBS 287.79	OOMYA1337-08	HQ708486, HQ643439
Pyt	*Pythium aphanidermatum*	Lev 1800	OOMYA385-07	HQ708489, HQ643442
Pyt	*Pythium aphanidermatum*	Lev 2150	OOMYA1607-08	HQ708488, HQ643441
Pyt	*Pythium aphanidermatum*	Lev 3014	OOMYA1657-08	HQ708487, HQ643440
Pyt	*Pythium aphanidermatum*	CBS 118.80	OOMYA010-07	HQ708485, HQ643438, HQ665084
Pyt	*Pythium apiculatum**	CBS 120945	OOMYA1341-08	HQ708490, HQ643443, HQ665092
Pyt	*Pythium apleroticum*	CBS 772.81	OOMYA234-07	HQ708491, HQ643444, HQ665296
Pyt	*Pythium aquatile*	BR 654	OOMYA1343-08	HQ708493, HQ643446
Pyt	*Pythium aquatile*	CBS 215.80	OOMYA048-07	HQ708492, HQ643445, HQ665153
Pyt	*Pythium aristosporum*	BR 166	OOMYA1346-08	HQ708495, HQ643448
Pyt	*Pythium aristosporum**	CBS 263.38	OOMYA1347-08	HQ708494, HQ643447, HQ665179
Pyt	*Pythium arrhenomanes*	BR 1028	OOMYA1361-08	HQ708519, HQ643472
Pyt	*Pythium arrhenomanes*	BR 122	OOMYA1349-08	HQ708518, HQ643471
Pyt	*Pythium arrhenomanes*	BR 140	OOMYA1350-08	HQ708517, HQ643470
Pyt	*Pythium arrhenomanes*	BR 155	OOMYA1412-08	HQ708516, HQ643469
Pyt	*Pythium arrhenomanes*	BR 605	OOMYA1658-08	HQ708515, HQ643468
Pyt	*Pythium arrhenomanes*	BR 606	OOMYA1688-08	HQ708514, HQ643467
Pyt	*Pythium arrhenomanes*	BR 615	OOMYA1689-08	HQ708513, HQ643466
Pyt	*Pythium arrhenomanes*	BR 618	OOMYA1690-08	HQ708512, HQ643465
Pyt	*Pythium arrhenomanes*	BR 619	OOMYA1691-08	HQ708511, HQ643464
Pyt	*Pythium arrhenomanes*	BR 625	OOMYA1413-08	HQ708510, HQ643463
Pyt	*Pythium arrhenomanes*	BR 667	OOMYA1351-08	HQ708509, HQ643462

Pyt	*Pythium arrhenomanes*	BR 712	OOMYA1352-08	HQ708508, HQ643461
Pyt	*Pythium arrhenomanes*	BR 729	OOMYA1667-08	HQ708507, HQ643460
Pyt	*Pythium arrhenomanes*	BR 817	OOMYA1353-08	HQ708506, HQ643459
Pyt	*Pythium arrhenomanes*	BR 889	OOMYA1354-08	HQ708496, HQ643449
Pyt	*Pythium arrhenomanes*	BR 975	OOMYA1659-08	HQ708505, HQ643458
Pyt	*Pythium arrhenomanes*	BR 980	OOMYA1356-08	HQ708504, HQ643457
Pyt	*Pythium arrhenomanes*	BR 981	OOMYA1357-08	HQ708503, HQ643456
Pyt	*Pythium arrhenomanes*	BR 982	OOMYA1358-08	HQ708502, HQ643455
Pyt	*Pythium arrhenomanes*	BR 983	OOMYA1359-08	HQ708501, HQ643454
Pyt	*Pythium arrhenomanes*	BR 985	OOMYA1360-08	HQ708500, HQ643453
Pyt	*Pythium arrhenomanes*	Lev 1538	OOMYA313-07	HQ708498, HQ643451
Pyt	*Pythium arrhenomanes*	Lev 1728	OOMYA1362-08	HQ708497, HQ643450
Pyt	*Pythium arrhenomanes**	CBS 324.62	OOMYA122-07	HQ708499, HQ643452, HQ665208
Pyt	*Pythium attrantheridium*	DAOM 230386	OOMYA1367-08	HQ708523, HQ643476
Pyt	*Pythium attrantheridium*	DAOM 230387	OOMYA1368-08	HQ708522, HQ643475
Pyt	*Pythium attrantheridium*	DAOM 230388	OOMYA1369-08	HQ708521, HQ643474
Pyt	*Pythium attrantheridium**	DAOM 230383	OOMYA1364-08	HQ708524, HQ643477, HQ665308
Pyt	*Pythium buismaniae**	CBS 288.31	OOMYA102-07	HQ708526, HQ643479, HQ665188
Pyt	*Pythium camurandrum*	CBS 124096	OOMYA1653-08	HQ708527, HQ643481
Pyt	*Pythium camurandrum**	BR 876	OOMYA1715-08	GQ244425, GQ244426
Pyt	*Pythium canariense**	CBS 112353	OOMYA1371-08	HQ708528, HQ643482, HQ665069
Pyt	*Pythium capillosum**	CBS 222.94	OOMYA061-07	HQ708529, HQ643483, HQ665164
Pyt	*Pythium carolinianum*	BR 161	OOMYA1637-08	HQ708534, HQ643488
Pyt	*Pythium carolinianum*	BR 786	OOMYA1692-08	HQ708533, HQ643487
Pyt	*Pythium carolinianum*	BR 803	OOMYA1714-08	HQ708532, HQ643486
Pyt	*Pythium carolinianum*	P19301	PHYTO142-10	HQ261478, HQ261731
Pyt	*Pythium carolinianum*	CBS 122658	OOMYA1605-08	HQ708531, HQ643485, HQ665110
Pyt	*Pythium carolinianum*	CBS 122659	OOMYA1373-08	HQ708530, HQ643484, HQ665111
Pyt	*Pythium catenulatum*	CBS 226.94	OOMYA1333-08	HQ708536, HQ643490
Pyt	*Pythium catenulatum*	CBS 461.75	OOMYA165-07	HQ708535, HQ643489
Pyt	*Pythium catenulatum*	Lev 1524	OOMYA302-07	HQ708539, HQ643493
Pyt	*Pythium catenulatum*	Lev 1532	OOMYA308-07	HQ708538, HQ643492
Pyt	*Pythium catenulatum*	Lev 1533	OOMYA1627-08	HQ708537, HQ643491
Pyt	*Pythium catenulatum*	CBS 842.68	OOMYA238-07	HQ708540, HQ643494, HQ665302
Pyt	*Pythium* cf. *dictyosporum*	ADC 0114	OOMYA1663-08	HQ708541, HQ643495
Pyt	*Pythium chondricola*	CBS 206.85	OOMYA1376-08	HQ708543, HQ643497
Pyt	*Pythium chondricola*	CBS 207.85	OOMYA1377-08	HQ708542, HQ643496
Pyt	*Pythium chondricola*	CBS 312.93	OOMYA1581-08	HQ708545, HQ643499
Pyt	*Pythium chondricola**	CBS 203.85	OOMYA040-07	HQ708544, HQ643498, HQ665149
Pyt	*Pythium coloratum*	BR 1063	OOMYA1391-08	HQ708553, HQ643507
Pyt	*Pythium coloratum*	BR 176	OOMYA1380-08	HQ708552, HQ643506
Pyt	*Pythium coloratum*	BR 323	OOMYA1382-08	HQ708551, HQ643505
Pyt	*Pythium coloratum*	BR 621	OOMYA1383-08	HQ708550, HQ643504
Pyt	*Pythium coloratum*	BR 677	OOMYA1385-08	HQ708549, HQ643503
Pyt	*Pythium coloratum*	BR 678	OOMYA1386-08	HQ708548, HQ643502
Pyt	*Pythium coloratum*	BR 908	OOMYA1389-08	HQ708546, HQ643500
Pyt	*Pythium coloratum**	CBS 154.64	OOMYA019-07	HQ708547, HQ643501, HQ665128
Pyt	*Pythium conidiophorum*	BR 160	OOMYA1399-08	HQ708559, HQ643513
Pyt	*Pythium conidiophorum*	BR 260	OOMYA1433-08	HQ708558, HQ643512
Pyt	*Pythium conidiophorum*	CBS 224.88	OOMYA063-07	HQ708554, HQ643508
Pyt	*Pythium conidiophorum*	Lev 1465	OOMYA279-07	HQ708557, HQ643511
Pyt	*Pythium conidiophorum*	Lev 1624	OOMYA346-07	HQ708556, HQ643510
Pyt	*Pythium conidiophorum*	CBS 223.88	OOMYA062-07	HQ708555, HQ643509, HQ665166
Pyt	*Pythium contiguanum**	CBS 221.94	OOMYA1392-08	HQ708560, HQ643514, HQ665162
Pyt	*Pythium cryptoirregulare**	CBS 118731	OOMYA1393-08	HQ708561, HQ643515, HQ665083
Pyt	*Pythium cylindrosporum*	DAOM 232335	OOMYA1394-08	HQ708563, HQ643517
Pyt	*Pythium cylindrosporum**	CBS 218.94	OOMYA053-07	HQ708562, HQ643516, HQ665157
Pyt	*Pythium cystogenes**	CBS 675.85	OOMYA1395-08	HQ708564, HQ643518, HQ665284
Pyt	*Pythium debaryanum*	CBS 752.96	OOMYA232-07	HQ708565, HQ643519, HQ665294
Pyt	*Pythium deliense*	CBS 114.84	OOMYA008-07	HQ708566, HQ643520
Pyt	*Pythium deliense*	Lev 2095	OOMYA018-07	HQ708567, HQ643521
Pyt	*Pythium deliense**	CBS 314.33	OOMYA118-07	HQ708568, HQ643522, HQ665204
Pyt	*Pythium diclinum*	CBS 526.74	OOMYA1398-08	HQ708569, HQ643523
Pyt	*Pythium diclinum*	CBS 664.79	OOMYA208-07	HQ708570, HQ643524, HQ665282
Pyt	*Pythium dimorphum**	CBS 406.72	OOMYA152-07	HQ708571, HQ643525, HQ665229
Pyt	*Pythium dissimile*	CBS 523.74	OOMYA1401-08	HQ708573, HQ643527
Pyt	*Pythium dissimile**	CBS 155.64	OOMYA020-07	HQ708572, HQ643526, HQ665130

Pyt	*Pythium dissotocum*	BR 127	OOMYA1402-08	HQ708576, HQ643530
Pyt	*Pythium dissotocum*	DAOM 229134	OOMYA1403-08	HQ708575, HQ643529
Pyt	*Pythium dissotocum*	CBS 166.68	OOMYA027-07	HQ708574, HQ643528, HQ665139
Pyt	*Pythium echinulatum*	CBS 281.64	OOMYA099-07	HQ708577, HQ643531, HQ665183
Pyt	*Pythium emineosum*	BR 836	OOMYA1578-08	GQ244424, GQ244428
Pyt	*Pythium emineosum**	BR 479	OOMYA1576-08	GQ244423, GQ244427
Pyt	*Pythium erinaceus*	Lev 1620	OOMYA344-07	HQ708579, HQ643535
Pyt	*Pythium erinaceus**	CBS 505.80	OOMYA172-07	HQ708578, HQ643534, HQ665243
Pyt	*Pythium flevoense*	CBS 233.72	OOMYA1405-08	HQ708583, HQ643539
Pyt	*Pythium flevoense*	CBS 236.72	OOMYA076-07	HQ708581, HQ643537
Pyt	*Pythium flevoense*	CBS 278.81	OOMYA1225-08	HQ708580, HQ643536, HQ665182
Pyt	*Pythium flevoense**	CBS 234.72	OOMYA073-07	HQ708582, HQ643538, HQ665170
Pyt	*Pythium folliculosum**	CBS 220.94	OOMYA057-07	HQ708584, HQ643540, HQ665160
Pyt	*Pythium glomeratum*	CBS 119165	OOMYA1406-08	HQ708588, HQ643544, HQ665085
Pyt	*Pythium glomeratum*	CBS 120914	OOMYA1407-08	HQ708587, HQ643543, HQ665091
Pyt	*Pythium glomeratum*	CBS 122644	OOMYA1408-08	HQ708586, HQ643542, HQ665097
Pyt	*Pythium glomeratum*	CBS 122651	OOMYA1410-08	HQ708585, HQ643541, HQ665104
Pyt	*Pythium graminicola*	CBS 327.62	OOMYA125-07	HQ708589, HQ643545, HQ665211
Pyt	*Pythium grandisporangium**	CBS 286.79	OOMYA100-07	HQ708590, HQ643546, HQ665187
Pyt	*Pythium helicandrum*	CBS 527.74	OOMYA1417-08	HQ708591, HQ643547
Pyt	*Pythium helicandrum*	CBS 694.79	OOMYA1418-08	HQ708594, HQ643550
Pyt	*Pythium helicandrum*	CBS 844.68	OOMYA239-07	HQ708593, HQ643549
Pyt	*Pythium helicandrum**	CBS 393.54	OOMYA147-07	HQ708592, HQ643548, HQ665225
Pyt	*Pythium heterothallicum*	ADC 9868	OOMYA1747-08	HQ708607, HQ643563
Pyt	*Pythium heterothallicum*	BR 440	OOMYA1422-08	HQ708606, HQ643562
Pyt	*Pythium heterothallicum*	BR 749	OOMYA1712-08	HQ708605, HQ643561
Pyt	*Pythium heterothallicum*	BR 806	OOMYA1425-08	HQ708604, HQ643560
Pyt	*Pythium heterothallicum*	BR 828	OOMYA1746-08	HQ708603, HQ643559
Pyt	*Pythium heterothallicum*	DAOM 229136	OOMYA1429-08	HQ708602, HQ643558
Pyt	*Pythium heterothallicum*	DAOM 229137	OOMYA1430-08	HQ708601, HQ643557
Pyt	*Pythium heterothallicum*	DAOM 229138	OOMYA1431-08	HQ708600, HQ643556
Pyt	*Pythium heterothallicum*	DAOM 229140	OOMYA441-07	HQ708599, HQ643555
Pyt	*Pythium heterothallicum*	Lev 1590	OOMYA326-07	HQ708598, HQ643554
Pyt	*Pythium heterothallicum*	CBS 122655	OOMYA1427-08	HQ708596, HQ643552, HQ665107
Pyt	*Pythium heterothallicum*	CBS 122656	OOMYA1428-08	HQ708595, HQ643551, HQ665108
Pyt	*Pythium heterothallicum**	CBS 450.67	OOMYA161-07	HQ708597, HQ643553, HQ665235
Pyt	*Pythium hydnosporum*	CBS 253.60	OOMYA085-07	HQ708608, HQ643564, HQ665175
Pyt	*Pythium hypogynum*	CBS 234.94	OOMYA074-07	HQ708609, HQ643565, HQ665171
Pyt	*Pythium inflatum*	CBS 168.68	OOMYA029-07	HQ708610, HQ643566, HQ665140
Pyt	*Pythium insidiosum*	CBS 577.85	OOMYA1435-08	HQ708613, HQ643569
Pyt	*Pythium insidiosum*	CBS 578.85	OOMYA1436-08	HQ708612, HQ643568
Pyt	*Pythium insidiosum*	CBS 580.85	OOMYA1437-08	HQ708611, HQ643567
Pyt	*Pythium insidiosum**	CBS 574.85	OOMYA193-07	HQ708614, HQ643570, HQ665273
Pyt	*Pythium intermedium*	BR 1042	OOMYA1447-08	HQ708623, HQ643579
Pyt	*Pythium intermedium*	BR 128	OOMYA1828-08	HQ708622, HQ643578
Pyt	*Pythium intermedium*	BR 339	OOMYA1438-08	HQ708621, HQ643577
Pyt	*Pythium intermedium*	BR 485	OOMYA1439-08	HQ708620, HQ643576
Pyt	*Pythium intermedium*	BR 707	OOMYA1440-08	HQ708619, HQ643575
Pyt	*Pythium intermedium*	BR 734	OOMYA1441-08	HQ708618, HQ643574
Pyt	*Pythium intermedium*	BR 869	OOMYA1800-08	HQ708617, HQ643573
Pyt	*Pythium intermedium*	BR 924	OOMYA1443-08	HQ708615, HQ643571
Pyt	*Pythium intermedium*	CBS 266.38	OOMYA091-07	HQ708616, HQ643572, HQ665180
Pyt	*Pythium irregulare*	BR 1000	OOMYA1533-08	HQ708711, HQ643667
Pyt	*Pythium irregulare*	BR 1001	OOMYA401-07	HQ708710, HQ643666
Pyt	*Pythium irregulare*	BR 1002	OOMYA1534-08	HQ708709, HQ643665
Pyt	*Pythium irregulare*	BR 1003	OOMYA1535-08	HQ708708, HQ643664
Pyt	*Pythium irregulare*	BR 1004	OOMYA1536-08	HQ708707, HQ643663
Pyt	*Pythium irregulare*	BR 1005	OOMYA1537-08	HQ708706, HQ643662
Pyt	*Pythium irregulare*	BR 1006	OOMYA1538-08	HQ708705, HQ643661
Pyt	*Pythium irregulare*	BR 1008	OOMYA1539-08	HQ708704, HQ643660
Pyt	*Pythium irregulare*	BR 1009	OOMYA1540-08	HQ708703, HQ643659
Pyt	*Pythium irregulare*	BR 1013	OOMYA402-07	HQ708702, HQ643658
Pyt	*Pythium irregulare*	BR 1014	OOMYA1543-08	HQ708701, HQ643657
Pyt	*Pythium irregulare*	BR 1015	OOMYA1544-08	HQ708700, HQ643656
Pyt	*Pythium irregulare*	BR 1016	OOMYA1545-08	HQ708699, HQ643655
Pyt	*Pythium irregulare*	BR 1017	OOMYA1546-08	HQ708698, HQ643654
Pyt	*Pythium irregulare*	BR 1018	OOMYA1547-08	HQ708697, HQ643653

Pyt	*Pythium irregulare*	BR 1019	OOMYA1548-08	HQ708696, HQ643652
Pyt	*Pythium irregulare*	BR 1021	OOMYA1549-08	HQ708695, HQ643651
Pyt	*Pythium irregulare*	BR 1022	OOMYA1550-08	HQ708694, HQ643650
Pyt	*Pythium irregulare*	BR 1039	OOMYA1553-08	HQ708693, HQ643649
Pyt	*Pythium irregulare*	BR 1040	OOMYA1554-08	HQ708692, HQ643648
Pyt	*Pythium irregulare*	BR 1051	OOMYA1555-08	HQ708691, HQ643647
Pyt	*Pythium irregulare*	BR 1052	OOMYA1556-08	HQ708690, HQ643646
Pyt	*Pythium irregulare*	BR 1068	OOMYA1558-08	HQ708689, HQ643645
Pyt	*Pythium irregulare*	BR 387	OOMYA1456-08	HQ708688, HQ643644
Pyt	*Pythium irregulare*	BR 469	OOMYA1457-08	HQ708687, HQ643643
Pyt	*Pythium irregulare*	BR 598	OOMYA1458-08	HQ708686, HQ643642
Pyt	*Pythium irregulare*	BR 629	OOMYA1460-08	HQ708685, HQ643641
Pyt	*Pythium irregulare*	BR 630	OOMYA1461-08	HQ708684, HQ643640
Pyt	*Pythium irregulare*	BR 631	OOMYA1462-08	HQ708683, HQ643639
Pyt	*Pythium irregulare*	BR 636	OOMYA331-07	HQ708682, HQ643638
Pyt	*Pythium irregulare*	BR 642	OOMYA1463-08	HQ708681, HQ643637
Pyt	*Pythium irregulare*	BR 722	OOMYA1465-08	HQ708680, HQ643636
Pyt	*Pythium irregulare*	BR 733	OOMYA1467-08	HQ708679, HQ643635
Pyt	*Pythium irregulare*	BR 772	OOMYA1469-08	HQ708678, HQ643634
Pyt	*Pythium irregulare*	BR 775	OOMYA1470-08	HQ708677, HQ643633
Pyt	*Pythium irregulare*	BR 778	OOMYA1471-08	HQ708676, HQ643632
Pyt	*Pythium irregulare*	BR 802	OOMYA1473-08	HQ708675, HQ643631
Pyt	*Pythium irregulare*	BR 804	OOMYA1474-08	HQ708674, HQ643630
Pyt	*Pythium irregulare*	BR 808	OOMYA1476-08	HQ708673, HQ643629
Pyt	*Pythium irregulare*	BR 811	OOMYA1477-08	HQ708672, HQ643628
Pyt	*Pythium irregulare*	BR 812	OOMYA1478-08	HQ708671, HQ643627
Pyt	*Pythium irregulare*	BR 815	OOMYA1479-08	HQ708670, HQ643626
Pyt	*Pythium irregulare*	BR 818	OOMYA1480-08	HQ708669, HQ643625
Pyt	*Pythium irregulare*	BR 819	OOMYA1693-08	HQ708668, HQ643624
Pyt	*Pythium irregulare*	BR 820	OOMYA1481-08	HQ708667, HQ643623
Pyt	*Pythium irregulare*	BR 821	OOMYA1694-08	HQ708666, HQ643622
Pyt	*Pythium irregulare*	BR 870	OOMYA1482-08	HQ708665, HQ643621
Pyt	*Pythium irregulare*	BR 900	OOMYA1486-08	HQ708639, HQ643595
Pyt	*Pythium irregulare*	BR 909	OOMYA1489-08	HQ708638, HQ643594
Pyt	*Pythium irregulare*	BR 911	OOMYA1490-08	HQ708637, HQ643593
Pyt	*Pythium irregulare*	BR 912	OOMYA1491-08	HQ708636, HQ643592
Pyt	*Pythium irregulare*	BR 914	OOMYA1492-08	HQ708635, HQ643591
Pyt	*Pythium irregulare*	BR 918	OOMYA1495-08	HQ708634, HQ643590
Pyt	*Pythium irregulare*	BR 920	OOMYA1496-08	HQ708633, HQ643589
Pyt	*Pythium irregulare*	BR 921	OOMYA1497-08	HQ708632, HQ643588
Pyt	*Pythium irregulare*	BR 923	OOMYA1498-08	HQ708631, HQ643587
Pyt	*Pythium irregulare*	BR 933	OOMYA338-07	HQ708630, HQ643586
Pyt	*Pythium irregulare*	BR 934	OOMYA1500-08	HQ708629, HQ643585
Pyt	*Pythium irregulare*	BR 940	OOMYA1506-08	HQ708628, HQ643584
Pyt	*Pythium irregulare*	BR 946	OOMYA1508-08	HQ708627, HQ643583
Pyt	*Pythium irregulare*	BR 947	OOMYA1509-08	HQ708626, HQ643582
Pyt	*Pythium irregulare*	BR 948	OOMYA1510-08	HQ708625, HQ643581
Pyt	*Pythium irregulare*	BR 959	OOMYA1514-08	HQ708624, HQ643580
Pyt	*Pythium irregulare*	BR 960	OOMYA1515-08	HQ708664, HQ643620
Pyt	*Pythium irregulare*	BR 961	OOMYA1516-08	HQ708663, HQ643619
Pyt	*Pythium irregulare*	BR 962	OOMYA339-07	HQ708662, HQ643618
Pyt	*Pythium irregulare*	BR 963	OOMYA1517-08	HQ708661, HQ643617
Pyt	*Pythium irregulare*	BR 964	OOMYA1518-08	HQ708660, HQ643616
Pyt	*Pythium irregulare*	BR 965	OOMYA1519-08	HQ708659, HQ643615
Pyt	*Pythium irregulare*	BR 966	OOMYA1520-08	HQ708658, HQ643614
Pyt	*Pythium irregulare*	BR 967	OOMYA1521-08	HQ708657, HQ643613
Pyt	*Pythium irregulare*	BR 969	OOMYA1522-08	HQ708656, HQ643612
Pyt	*Pythium irregulare*	BR 970	OOMYA1523-08	HQ708655, HQ643611
Pyt	*Pythium irregulare*	BR 971	OOMYA1524-08	HQ708654, HQ643610
Pyt	*Pythium irregulare*	BR 972	OOMYA1525-08	HQ708653, HQ643609
Pyt	*Pythium irregulare*	BR 979	OOMYA1698-08	HQ708652, HQ643608
Pyt	*Pythium irregulare*	BR 994	OOMYA1528-08	HQ708651, HQ643607
Pyt	*Pythium irregulare*	BR 995	OOMYA1529-08	HQ708650, HQ643606
Pyt	*Pythium irregulare*	BR 996	OOMYA1530-08	HQ708649, HQ643605
Pyt	*Pythium irregulare*	BR 997	OOMYA1531-08	HQ708648, HQ643604
Pyt	*Pythium irregulare*	BR 999	OOMYA1532-08	HQ708647, HQ643603
Pyt	*Pythium irregulare*	CBS 749.96	OOMYA1559-08	HQ708646, HQ643602

Pyt	*Pythium irregulare*	DAOM 232336	OOMYA1561-08	HQ708645, HQ643601
Pyt	*Pythium irregulare*	Lev 1680	OOMYA1762-08	HQ708644, HQ643600
Pyt	*Pythium irregulare*	Lev 2120	OOMYA1563-08	HQ708643, HQ643599
Pyt	*Pythium irregulare*	Lev 2151	OOMYA433-07	HQ708642, HQ643598
Pyt	*Pythium irregulare*	Lev 3105	OOMYA1566-08	HQ708641, HQ643597
Pyt	*Pythium irregulare*	ADC 0838	OOMYA1453-08	HQ708712, HQ643668, HQ665051
Pyt	*Pythium irregulare*	CBS 250.28	OOMYA083-07	HQ708640, HQ643596, HQ665172
Pyt	*Pythium iwayamai*	CBS 156.64	OOMYA021-07	HQ708713, HQ643669, HQ665131
Pyt	*Pythium kashmirense**	CBS 122908	OOMYA1569-08	HQ708715, HQ643671, HQ665118
Pyt	*Pythium kunmingense**	CBS 550.88	OOMYA182-07	HQ708716, HQ643672, HQ665259
Pyt	*Pythium longandrum*	CBS 122660	OOMYA1572-08	HQ708722, HQ643678, HQ665112
Pyt	*Pythium longandrum**	CBS 112355	OOMYA1571-08	HQ708723, HQ643679, HQ665071
Pyt	*Pythium longisporangium**	CBS 122646	OOMYA1573-08	HQ708724, HQ643680, HQ665099
Pyt	*Pythium lucens**	CBS 113342	OOMYA1574-08	HQ708725, HQ643681, HQ665077
Pyt	*Pythium lutarium**	CBS 222.88	OOMYA060-07	HQ708726, HQ643682, HQ665163
Pyt	*Pythium lycopersicum**	CBS 122909	OOMYA1575-08	HQ708727, HQ643683, HQ665119
Pyt	*Pythium macrosporum*	ADC 0029	OOMYA1776-08	HQ708730, HQ643686
Pyt	*Pythium macrosporum**	CBS 574.80	OOMYA192-07	HQ708728, HQ643684, HQ665272
Pyt	*Pythium mamillatum*	BR 648	OOMYA1580-08	HQ708733, HQ643689
Pyt	*Pythium mamillatum*	BR 765	OOMYA340-07	HQ708732, HQ643688
Pyt	*Pythium mamillatum*	CBS 251.28	OOMYA084-07	HQ708731, HQ643687, HQ665173
Pyt	*Pythium marsipium*	CBS 773.81	OOMYA235-07	HQ708734, HQ643690, HQ665297
Pyt	*Pythium mastophorum*	ADC 0158	OOMYA1582-08	HQ708736, HQ643692
Pyt	*Pythium mastophorum*	CBS 375.72	OOMYA139-07	HQ708735, HQ643691, HQ665220
Pyt	*Pythium megalacanthum*	DAOM 229154	OOMYA1586-08	HQ708737, HQ643693
Pyt	*Pythium middletonii*	CBS 528.74	OOMYA177-07	HQ708738, HQ643694, HQ665249
Pyt	*Pythium minus*	CBS 122657	OOMYA1589-08	HQ708739, HQ643695, HQ665109
Pyt	*Pythium minus**	CBS 226.88	OOMYA066-07	HQ708740, HQ643696, HQ665168
Pyt	*Pythium monospermum*	BR 1031	OOMYA1681-08	HQ708743, HQ643699
Pyt	*Pythium monospermum*	BR 1032	OOMYA1682-08	HQ708742, HQ643698
Pyt	*Pythium monospermum*	CBS 158.73	OOMYA025-07	HQ708741, HQ643697, HQ665137
Pyt	*Pythium multisporum**	CBS 470.50	OOMYA168-07	HQ708744, HQ643700, HQ665239
Pyt	*Pythium myriotylum*	CBS 695.79	OOMYA218-07	HQ708748, HQ643704
Pyt	*Pythium myriotylum*	Lev 1529	OOMYA306-07	HQ708747, HQ643703
Pyt	*Pythium myriotylum*	Lev 1737	OOMYA376-07	HQ708746, HQ643702
Pyt	*Pythium myriotylum*	CBS 254.70	OOMYA086-07	HQ708745, HQ643701, HQ665176
Pyt	*Pythium nagaii*	BR 602	OOMYA330-07	HQ708751, HQ643707
Pyt	*Pythium nagaii*	BR 646	OOMYA1612-08	HQ708750, HQ643706
Pyt	*Pythium nagaii*	CBS 779.96	OOMYA1597-08	HQ708749, HQ643705, HQ665299
Pyt	*Pythium nodosum*	CBS 122661	OOMYA1614-08	HQ708752, HQ643708, HQ665113
Pyt	*Pythium nodosum**	CBS 102274	OOMYA1598-08	HQ708753, HQ643709, HQ665055
Pyt	*Pythium nunn*	Lev 2098	OOMYA1599-08	HQ708754, HQ643710
Pyt	*Pythium nunn**	CBS 808.96	OOMYA237-07	HQ708755, HQ643711, HQ665300
Pyt	*Pythium okanoganense*	CBS 701.83	OOMYA222-07	HQ708757, HQ643713
Pyt	*Pythium okanoganense**	CBS 315.81	OOMYA119-07	HQ708758, HQ643714, HQ665205
Pyt	*Pythium oligandrum*	BR 252	OOMYA1602-08	HQ708760, HQ643716
Pyt	*Pythium oligandrum*	CBS 382.34	OOMYA145-07	HQ708759, HQ643715, HQ665223
Pyt	*Pythium oopapillum*	BR 180	OOMYA1381-08	FJ655180, FJ655176
Pyt	*Pythium oopapillum*	BR 641	OOMYA1342-08	FJ655179, FJ655175
Pyt	*Pythium oopapillum**	BR 632	OOMYA1384-08	FJ655178, FJ655174
Pyt	*Pythium ornacarpum**	CBS 112350	OOMYA1603-08	HQ708762, HQ643721, HQ665066
Pyt	*Pythium ornamentatum*	CBS 122665	OOMYA1604-08	HQ708763, HQ643722, HQ665117
Pyt	*Pythium orthogonon**	CBS 376.72	OOMYA140-07	HQ708764, HQ643723, HQ665221
Pyt	*Pythium pachycaule*	BR 679	OOMYA1387-08	HQ708768, HQ643727
Pyt	*Pythium pachycaule*	CBS 224.94	OOMYA064-07	HQ708767, HQ643726
Pyt	*Pythium pachycaule*	CBS 225.94	OOMYA1606-08	HQ708766, HQ643725
Pyt	*Pythium pachycaule**	CBS 227.88	OOMYA067-07	HQ708765, HQ643724, HQ665169
Pyt	*Pythium paddicum*	CBS 698.83	OOMYA219-07	HQ708769, HQ643728, HQ665290
Pyt	*Pythium papilogynum*	CBS 122648	OOMYA1608-08	HQ708770, HQ643729, HQ665101
Pyt	*Pythium paroecandrum*	ADC 9909	OOMYA1655-08	HQ708778, HQ643737
Pyt	*Pythium paroecandrum*	ADC 9910	OOMYA1656-08	HQ708777, HQ643736
Pyt	*Pythium paroecandrum*	BR 601	OOMYA1459-08	HQ708776, HQ643735
Pyt	*Pythium paroecandrum*	BR 773	OOMYA1852-08	HQ708775, HQ643734
Pyt	*Pythium paroecandrum*	BR 774	OOMYA1670-08	HQ708774, HQ643733
Pyt	*Pythium paroecandrum*	BR 807	OOMYA1475-08	HQ708773, HQ643732
Pyt	*Pythium paroecandrum*	BR 929	OOMYA1499-08	HQ708771, HQ643730
Pyt	*Pythium paroecandrum*	CBS 157.64	OOMYA022-07	HQ708772, HQ643731, HQ665133

Pyt	*Pythium parvum**	CBS 225.88	OOMYA065-07	HQ708779, HQ643738, HQ665167
Pyt	*Pythium pectinolyticum**	CBS 122643	OOMYA1615-08	HQ708780, HQ643739, HQ665096
Pyt	*Pythium periilum*	CBS 169.68	OOMYA030-07	HQ708781, HQ643740, HQ665141
Pyt	*Pythium periplocum*	CBS 170.68	OOMYA1618-08	HQ708782, HQ643741
Pyt	*Pythium periplocum*	CBS 122664	OOMYA1617-08	HQ708783, HQ643742, HQ665116
Pyt	*Pythium periplocum**	CBS 289.31	OOMYA103-07	HQ708784, HQ643743, HQ665189
Pyt	*Pythium perplexum*	BR 984	OOMYA1619-08	HQ708786, HQ643745
Pyt	*Pythium perplexum*	CBS 674.85	OOMYA210-07	HQ708785, HQ643744, HQ665283
Pyt	*Pythium phragmitis**	CBS 117104	OOMYA1620-08	HQ708787, HQ643746, HQ665081
Pyt	*Pythium pleroticum*	CBS 776.81	OOMYA236-07	HQ708789, HQ643748, HQ665298
Pyt	*Pythium plurisporium*	ADC 9824	OOMYA1623-08	HQ708791, HQ643750
Pyt	*Pythium plurisporium**	CBS 100530	OOMYA1624-08	HQ708790, HQ643749, HQ665052
Pyt	*Pythium polymastum*	CBS 811.70	OOMYA1625-08	HQ708793, HQ643752, HQ665301
Pyt	*Pythium porphyrae*	CBS 369.79	OOMYA137-07	HQ708794, HQ643753, HQ665218
Pyt	*Pythium prolatum**	CBS 845.68	OOMYA240-07	HQ708795, HQ643754, HQ665303
Pyt	*Pythium pyrilobum**	CBS 158.64	OOMYA024-07	HQ708796, HQ643755, HQ665136
Pyt	*Pythium radiosum**	CBS 217.94	OOMYA051-07	HQ708797, HQ643756, HQ665156
Pyt	*Pythium rhizo-oryzae**	CBS 119169	OOMYA1628-08	HQ708798, HQ643757, HQ665087
Pyt	*Pythium rhizosaccharum*	CBS 122652	OOMYA1630-08	HQ708800, HQ643759, HQ665105
Pyt	*Pythium rhizosaccharum*	CBS 122654	OOMYA1632-08	HQ708799, HQ643758, HQ665106
Pyt	*Pythium rhizosaccharum**	CBS 112356	OOMYA1629-08	HQ708801, HQ643760, HQ665072
Pyt	*Pythium rostratifingens*	BR 1061	OOMYA1699-08	HQ708806, HQ643765
Pyt	*Pythium rostratifingens*	BR 197	OOMYA351-07	HQ708807, HQ643766
Pyt	*Pythium rostratifingens*	BR 627	OOMYA352-07	HQ708805, HQ643764
Pyt	*Pythium rostratifingens*	CBS 383.34	OOMYA1634-08	HQ708803, HQ643762
Pyt	*Pythium rostratifingens*	Lev 3023	OOMYA1635-08	HQ708804, HQ643763
Pyt	*Pythium rostratifingens**	CBS 115464	OOMYA1633-08	HQ708802, HQ643761, HQ665080
Pyt	*Pythium rostratum*	CBS 533.74	OOMYA179-07	HQ708808, HQ643767, HQ665252
Pyt	*Pythium salpingophorum*	BR 1024	OOMYA1642-08	HQ708811, HQ643770
Pyt	*Pythium salpingophorum*	BR 750	OOMYA1640-08	HQ708810, HQ643769
Pyt	*Pythium salpingophorum*	CBS 471.50	OOMYA169-07	HQ708809, HQ643768, HQ665240
Pyt	*Pythium scleroteichum**	CBS 294.37	OOMYA109-07	HQ708812, HQ643771, HQ665192
Pyt	*Pythium segnitium**	CBS 112354	OOMYA1644-08	HQ708813, HQ643772, HQ665070
Pyt	*Pythium senticosum**	CBS 122490	OOMYA1645-08	HQ708814, HQ643773, HQ665093
Pyt	*Pythium* sp.	CBS 113341	OOMYA1639-08	HQ708818, HQ643777, HQ665076
Pyt	*Pythium* sp.	CBS 750.96	OOMYA231-07	HQ708817, HQ643776, HQ665293
Pyt	*Pythium* sp. *"balticum"*	CBS 122649	OOMYA1370-08	HQ708525, HQ643478, HQ665102
Pyt	*Pythium* sp. *"Group F"*	ADC 0014	OOMYA1706-08	HQ708826, HQ643785
Pyt	*Pythium* sp. *"Group F"*	ADC 9425	OOMYA1707-08	HQ708825, HQ643784
Pyt	*Pythium* sp. *"Group F"*	ADC 9426	OOMYA1708-08	HQ708824, HQ643783
Pyt	*Pythium* sp. *"Group F"*	ADC 9518	OOMYA1709-08	HQ708823, HQ643782
Pyt	*Pythium* sp. *"Group F"*	ADC 9989	OOMYA1710-08	HQ708822, HQ643781
Pyt	*Pythium* sp. *"Group F"*	BR 1041	OOMYA1716-08	HQ708821, HQ643780
Pyt	*Pythium* sp. *"Group F"*	BR 1044	OOMYA1717-08	HQ708820, HQ643779
Pyt	*Pythium* sp. *"Group F"*	BR 710	OOMYA335-07	HQ708830, HQ643789
Pyt	*Pythium* sp. *"Group F"*	BR 739	OOMYA1711-08	HQ708829, HQ643788
Pyt	*Pythium* sp. *"Group F"*	BR 777	OOMYA1713-08	HQ708828, HQ643787
Pyt	*Pythium* sp. *"jasmonium"*	DAOM 229150	OOMYA422-07	HQ708714, HQ643670
Pyt	*Pythium* sp. *"jasmonium"*	CBS 101876	OOMYA1568-08	HQ708819, HQ643778, HQ665054
Pyt	*Pythium* sp. *"spiculacarpum"*	CBS 122647	OOMYA1781-08	HQ708815, HQ643774, HQ665100
Pyt	*Pythium* sp. *"tumidum"*	CBS 223.94	OOMYA1827-08	HQ708816, HQ643775
Pyt	*Pythium* sp. nov.	ADC 0013	OOMYA1587-08	HQ708876, HQ643835
Pyt	*Pythium* sp. nov.	ADC 0110	OOMYA1588-08	HQ708875, HQ643834
Pyt	*Pythium* sp. nov.	ADC 0111	OOMYA1434-08	HQ708874, HQ643833
Pyt	*Pythium* sp. nov.	ADC 9407	OOMYA1777-08	HQ708873, HQ643832
Pyt	*Pythium* sp. nov.	ADC 9409	OOMYA1646-08	HQ708872, HQ643831
Pyt	*Pythium* sp. nov.	ADC 9421	OOMYA1647-08	HQ708871, HQ643830
Pyt	*Pythium* sp. nov.	ADC 9759	OOMYA1651-08	HQ708870, HQ643829
Pyt	*Pythium* sp. nov.	ADC 9966	OOMYA1748-08	HQ708869, HQ643828
Pyt	*Pythium* sp. nov.	ADC 9982	OOMYA1749-08	HQ708868, HQ643827
Pyt	*Pythium* sp. nov.	BR 1033	OOMYA1822-08	HQ708850, HQ643809
Pyt	*Pythium* sp. nov.	BR 1034	OOMYA1414-08	HQ708849, HQ643808
Pyt	*Pythium* sp. nov.	BR 1047	OOMYA1448-08	HQ708848, HQ643807
Pyt	*Pythium* sp. nov.	BR 1062	OOMYA1613-08	HQ708847, HQ643806
Pyt	*Pythium* sp. nov.	BR 147	OOMYA1680-08	HQ708867, HQ643826
Pyt	*Pythium* sp. nov.	BR 205	OOMYA1685-08	HQ708866, HQ643825
Pyt	*Pythium* sp. nov.	BR 574	OOMYA400-07	HQ708865, HQ643824

Pyt	*Pythium* sp. nov.	BR 613	OOMYA1720-08	HQ708864, HQ643823
Pyt	*Pythium* sp. nov.	BR 655	OOMYA1344-08	HQ708863, HQ643822
Pyt	*Pythium* sp. nov.	BR 688	OOMYA1388-08	HQ708862, HQ643821
Pyt	*Pythium* sp. nov.	BR 706	OOMYA333-07	HQ708861, HQ643820
Pyt	*Pythium* sp. nov.	BR 779	OOMYA1472-08	HQ708860, HQ643819
Pyt	*Pythium* sp. nov.	BR 798	OOMYA1577-08	HQ708859, HQ643818
Pyt	*Pythium* sp. nov.	BR 879	OOMYA1483-08	HQ708858, HQ643817
Pyt	*Pythium* sp. nov.	BR 887	OOMYA353-07	HQ708854, HQ643813
Pyt	*Pythium* sp. nov.	BR 901	OOMYA1487-08	HQ708853, HQ643812
Pyt	*Pythium* sp. nov.	BR 902	OOMYA1488-08	HQ708852, HQ643811
Pyt	*Pythium* sp. nov.	BR 951	OOMYA1445-08	HQ708851, HQ643810
Pyt	*Pythium* sp. nov.	CBS 232.94	OOMYA1616-08	HQ708855, HQ643814
Pyt	*Pythium* sp. nov.	CBS 607.81	OOMYA1700-08	HQ708857, HQ643816
Pyt	*Pythium* sp. nov.	CBS 633.85	OOMYA1702-08	HQ708856, HQ643815
Pyt	*Pythium* sp. nov.	DAOM 229155	OOMYA1601-08	HQ708846, HQ643805
Pyt	*Pythium* sp. nov.	Lev 1457	OOMYA275-07	HQ708845, HQ643804
Pyt	*Pythium* sp. nov.	Lev 1523	OOMYA301-07	HQ708844, HQ643803
Pyt	*Pythium* sp. nov.	Lev 2156	OOMYA436-07	HQ708843, HQ643802
Pyt	*Pythium* sp. nov.	Lev 2168	OOMYA440-07	HQ708842, HQ643801
Pyt	*Pythium* sp. nov.	Lev 3062	OOMYA1672-08	HQ708841, HQ643800
Pyt	*Pythium* sp. nov.	Lev 3063	OOMYA1673-08	HQ708840, HQ643799
Pyt	*Pythium* sp. nov.	Lev 3106	OOMYA1567-08	HQ708839, HQ643798
Pyt	*Pythium* sp. nov.	P16024	PHYTO116-10	HQ261487, HQ261740
Pyt	*Pythium* sp. nov.	P19400	PHYTO143-10	HQ261486, HQ261739
Pyt	*Pythium* sp. nov.	P19448	PHYTO144-10	HQ261485, HQ261738
Pyt	*Pythium* sp. nov.	P19510	PHYTO145-10	HQ261484, HQ261737
Pyt	*Pythium* sp. nov.	P8201	PHYTO225-10	HQ261483, HQ261736
Pyt	*Pythium* sp. nov.	P8204	PHYTO226-10	HQ261482, HQ261735
Pyt	*Pythium* sp. nov.	P8207	PHYTO227-10	HQ261481, HQ261734
Pyt	*Pythium* sp. nov.	P8209	PHYTO228-10	HQ261480, HQ261733
Pyt	*Pythium* sp. nov.	P8212	PHYTO229-10	HQ261479, HQ261732
Pyt	*Pythium spiculum**	CBS 122645	OOMYA1782-08	HQ708831, HQ643790, HQ665098
Pyt	*Pythium spinosum*	CBS 276.67	OOMYA098-07	HQ708833, HQ643792
Pyt	*Pythium spinosum*	Lev 1526	OOMYA304-07	HQ708835, HQ643794
Pyt	*Pythium spinosum*	CBS 122663	OOMYA1783-08	HQ708832, HQ643791, HQ665115
Pyt	*Pythium spinosum*	CBS 275.67	OOMYA1784-08	HQ708834, HQ643793, HQ665181
Pyt	*Pythium splendens*	BR 788	OOMYA1786-08	HQ708838, HQ643797
Pyt	*Pythium splendens*	Lev 1497	OOMYA1759-08	HQ708837, HQ643796
Pyt	*Pythium splendens*	CBS 462.48	OOMYA166-07	HQ708836, HQ643795, HQ665237
Pyt	*Pythium sukuiense**	CBS 110030	OOMYA1787-08	HQ708877, HQ643836, HQ665059
Pyt	*Pythium sulcatum*	BR 113	OOMYA1788-08	HQ708884, HQ643843
Pyt	*Pythium sulcatum*	BR 146	OOMYA1789-08	HQ708883, HQ643842
Pyt	*Pythium sulcatum*	BR 652	OOMYA1791-08	HQ708882, HQ643841
Pyt	*Pythium sulcatum*	BR 653	OOMYA341-07	HQ708885, HQ643844
Pyt	*Pythium sulcatum*	BR 708	OOMYA334-07	HQ708881, HQ643840
Pyt	*Pythium sulcatum*	BR 709	OOMYA1792-08	HQ708880, HQ643839
Pyt	*Pythium sulcatum*	Lev 3111	OOMYA1794-08	HQ708879, HQ643838
Pyt	*Pythium sulcatum**	CBS 603.73	OOMYA205-07	HQ708878, HQ643837, HQ665281
Pyt	*Pythium sylvaticum*	BR 1045	OOMYA1807-08	HQ708893, HQ643852
Pyt	*Pythium sylvaticum*	BR 1069	OOMYA1738-08	HQ708892, HQ643851
Pyt	*Pythium sylvaticum*	BR 171	OOMYA1455-08	HQ708891, HQ643850
Pyt	*Pythium sylvaticum*	BR 179	OOMYA1797-08	HQ708890, HQ643849
Pyt	*Pythium sylvaticum*	BR 599	OOMYA1798-08	HQ708889, HQ643848
Pyt	*Pythium sylvaticum*	BR 647	OOMYA332-07	HQ708888, HQ643847
Pyt	*Pythium sylvaticum*	Lev 1544	OOMYA317-07	HQ708887, HQ643846
Pyt	*Pythium sylvaticum*	P15580	PHYTO112-10	HQ261488, HQ261741
Pyt	*Pythium sylvaticum**	CBS 453.67	OOMYA163-07	HQ708886, HQ643845, HQ665236
Pyt	*Pythium takayamanum*	CBS 122492	OOMYA1814-08	HQ708894, HQ643853, HQ665095
Pyt	*Pythium takayamanum**	CBS 122491	OOMYA1813-08	HQ708895, HQ643854, HQ665094
Pyt	*Pythium tardicrescens*	Lev 1534	OOMYA309-07	HQ708896, HQ643855
Pyt	*Pythium terrestris*	ADC 9906	OOMYA1654-08	HQ708899, HQ643858
Pyt	*Pythium terrestris*	BR 922	OOMYA1801-08	HQ708897, HQ643856
Pyt	*Pythium terrestris**	CBS 112352	OOMYA1818-08	HQ708898, HQ643857, HQ665068
Pyt	*Pythium torulosum*	CBS 316.33	OOMYA120-07	HQ708900, HQ643859, HQ665206
Pyt	*Pythium tracheiphilum*	BR 659	OOMYA1823-08	HQ708904, HQ643863
Pyt	*Pythium tracheiphilum*	BR 931	OOMYA1825-08	HQ708902, HQ643861
Pyt	*Pythium tracheiphilum*	BR 932	OOMYA1826-08	HQ708901, HQ643860

Pyt	*Pythium tracheiphilum**	CBS 323.65	OOMYA121-07	HQ708903, HQ643862, HQ665207
Pyt	*Pythium ultimum* var. *sporangiiferum*	BR 651	OOMYA1836-08	HQ708921, HQ643880
Pyt	*Pythium ultimum* var. *sporangiiferum**	CBS 219.65	OOMYA054-07	HQ708920, HQ643879, HQ665158
Pyt	*Pythium ultimum* var. *ultimum*	ADC 9967	OOMYA1718-08	HQ708919, HQ643878
Pyt	*Pythium ultimum* var. *ultimum*	BR 1036	OOMYA1882-08	HQ708918, HQ643877
Pyt	*Pythium ultimum* var. *ultimum*	BR 1037	OOMYA1883-08	HQ708917, HQ643876
Pyt	*Pythium ultimum* var. *ultimum*	BR 1038	OOMYA1884-08	HQ708916, HQ643875
Pyt	*Pythium ultimum* var. *ultimum*	BR 1054	OOMYA1886-08	HQ708915, HQ643874
Pyt	*Pythium ultimum* var. *ultimum*	BR 1060	OOMYA407-07	HQ708914, HQ643873
Pyt	*Pythium ultimum* var. *ultimum*	BR 1064	OOMYA1888-08	HQ708913, HQ643872
Pyt	*Pythium ultimum* var. *ultimum*	BR 1065	OOMYA408-07	HQ708912, HQ643871
Pyt	*Pythium ultimum* var. *ultimum*	BR 1089	OOMYA1889-08	HQ708911, HQ643870
Pyt	*Pythium ultimum* var. *ultimum*	BR 144	OOMYA386-07	HQ708984, HQ643943
Pyt	*Pythium ultimum* var. *ultimum*	BR 229	OOMYA1668-08	HQ708983, HQ643942
Pyt	*Pythium ultimum* var. *ultimum*	BR 319	OOMYA1829-08	HQ708982, HQ643941
Pyt	*Pythium ultimum* var. *ultimum*	BR 511	OOMYA1830-08	HQ708981, HQ643940
Pyt	*Pythium ultimum* var. *ultimum*	BR 583	OOMYA1831-08	HQ708980, HQ643939
Pyt	*Pythium ultimum* var. *ultimum*	BR 600	OOMYA1832-08	HQ708979, HQ643938
Pyt	*Pythium ultimum* var. *ultimum*	BR 612	OOMYA1719-08	HQ708978, HQ643937
Pyt	*Pythium ultimum* var. *ultimum*	BR 628	OOMYA1833-08	HQ708977, HQ643936
Pyt	*Pythium ultimum* var. *ultimum*	BR 640	OOMYA1834-08	HQ708976, HQ643935
Pyt	*Pythium ultimum* var. *ultimum*	BR 656	OOMYA1721-08	HQ708975, HQ643934
Pyt	*Pythium ultimum* var. *ultimum*	BR 657	OOMYA1722-08	HQ708974, HQ643933
Pyt	*Pythium ultimum* var. *ultimum*	BR 658	OOMYA1723-08	HQ708973, HQ643932
Pyt	*Pythium ultimum* var. *ultimum*	BR 718	OOMYA389-07	HQ708972, HQ643931
Pyt	*Pythium ultimum* var. *ultimum*	BR 736	OOMYA1840-08	HQ708971, HQ643930
Pyt	*Pythium ultimum* var. *ultimum*	BR 737	OOMYA1841-08	HQ708970, HQ643929
Pyt	*Pythium ultimum* var. *ultimum*	BR 738	OOMYA1842-08	HQ708969, HQ643928
Pyt	*Pythium ultimum* var. *ultimum*	BR 745	OOMYA1843-08	HQ708968, HQ643927
Pyt	*Pythium ultimum* var. *ultimum*	BR 747	OOMYA1844-08	HQ708967, HQ643926
Pyt	*Pythium ultimum* var. *ultimum*	BR 748	OOMYA1845-08	HQ708966, HQ643925
Pyt	*Pythium ultimum* var. *ultimum*	BR 754	OOMYA1847-08	HQ708965, HQ643924
Pyt	*Pythium ultimum* var. *ultimum*	BR 755	OOMYA1729-08	HQ708964, HQ643923
Pyt	*Pythium ultimum* var. *ultimum*	BR 759	OOMYA1733-08	HQ708963, HQ643922
Pyt	*Pythium ultimum* var. *ultimum*	BR 760	OOMYA1734-08	HQ708962, HQ643921
Pyt	*Pythium ultimum* var. *ultimum*	BR 763	OOMYA1848-08	HQ708961, HQ643920
Pyt	*Pythium ultimum* var. *ultimum*	BR 768	OOMYA1850-08	HQ708960, HQ643919
Pyt	*Pythium ultimum* var. *ultimum*	BR 776	OOMYA1853-08	HQ708959, HQ643918
Pyt	*Pythium ultimum* var. *ultimum*	BR 781	OOMYA393-07	HQ708958, HQ643917
Pyt	*Pythium ultimum* var. *ultimum*	BR 783	OOMYA404-07	HQ708957, HQ643916
Pyt	*Pythium ultimum* var. *ultimum*	BR 784	OOMYA1854-08	HQ708956, HQ643915
Pyt	*Pythium ultimum* var. *ultimum*	BR 792	OOMYA1855-08	HQ708955, HQ643914
Pyt	*Pythium ultimum* var. *ultimum*	BR 793	OOMYA1856-08	HQ708954, HQ643913
Pyt	*Pythium ultimum* var. *ultimum*	BR 810	OOMYA387-07	HQ708953, HQ643912
Pyt	*Pythium ultimum* var. *ultimum*	BR 813	OOMYA1857-08	HQ708952, HQ643911
Pyt	*Pythium ultimum* var. *ultimum*	BR 814	OOMYA1858-08	HQ708951, HQ643910
Pyt	*Pythium ultimum* var. *ultimum*	BR 816	OOMYA1859-08	HQ708950, HQ643909
Pyt	*Pythium ultimum* var. *ultimum*	BR 822	OOMYA1740-08	HQ708949, HQ643908
Pyt	*Pythium ultimum* var. *ultimum*	BR 823	OOMYA1741-08	HQ708948, HQ643907
Pyt	*Pythium ultimum* var. *ultimum*	BR 824	OOMYA1742-08	HQ708947, HQ643906
Pyt	*Pythium ultimum* var. *ultimum*	BR 825	OOMYA1743-08	HQ708946, HQ643905
Pyt	*Pythium ultimum* var. *ultimum*	BR 826	OOMYA1744-08	HQ708945, HQ643904
Pyt	*Pythium ultimum* var. *ultimum*	BR 827	OOMYA1745-08	HQ708944, HQ643903
Pyt	*Pythium ultimum* var. *ultimum*	BR 833	OOMYA1860-08	HQ708943, HQ643902
Pyt	*Pythium ultimum* var. *ultimum*	BR 835	OOMYA1861-08	HQ708942, HQ643901
Pyt	*Pythium ultimum* var. *ultimum*	BR 841	OOMYA392-07	HQ708941, HQ643900
Pyt	*Pythium ultimum* var. *ultimum*	BR 842	OOMYA1864-08	HQ708940, HQ643899
Pyt	*Pythium ultimum* var. *ultimum*	BR 843	OOMYA1865-08	HQ708939, HQ643898
Pyt	*Pythium ultimum* var. *ultimum*	BR 844	OOMYA1866-08	HQ708938, HQ643897
Pyt	*Pythium ultimum* var. *ultimum*	BR 845	OOMYA1867-08	HQ708937, HQ643896
Pyt	*Pythium ultimum* var. *ultimum*	BR 847	OOMYA1868-08	HQ708936, HQ643895
Pyt	*Pythium ultimum* var. *ultimum*	BR 848	OOMYA1869-08	HQ708935, HQ643894
Pyt	*Pythium ultimum* var. *ultimum*	BR 849	OOMYA1870-08	HQ708934, HQ643893
Pyt	*Pythium ultimum* var. *ultimum*	BR 850	OOMYA1871-08	HQ708933, HQ643892
Pyt	*Pythium ultimum* var. *ultimum*	BR 851	OOMYA1872-08	HQ708932, HQ643891
Pyt	*Pythium ultimum* var. *ultimum*	BR 854	OOMYA1873-08	HQ708931, HQ643890
Pyt	*Pythium ultimum* var. *ultimum*	BR 858	OOMYA1739-08	HQ708930, HQ643889

Pyt	*Pythium ultimum* var. *ultimum*	BR 861	OOMYA1876-08	HQ708929, HQ643888
Pyt	*Pythium ultimum* var. *ultimum*	BR 863	OOMYA1751-08	HQ708928, HQ643887
Pyt	*Pythium ultimum* var. *ultimum*	BR 864	OOMYA1752-08	HQ708927, HQ643886
Pyt	*Pythium ultimum* var. *ultimum*	BR 867	OOMYA1877-08	HQ708926, HQ643885
Pyt	*Pythium ultimum* var. *ultimum*	BR 930	OOMYA1878-08	HQ708923, HQ643882
Pyt	*Pythium ultimum* var. *ultimum*	BR 944	OOMYA1696-08	HQ708922, HQ643881
Pyt	*Pythium ultimum* var. *ultimum*	BR 987	OOMYA1879-08	HQ708925, HQ643884
Pyt	*Pythium ultimum* var. *ultimum*	BR 988	OOMYA1880-08	HQ708924, HQ643883
Pyt	*Pythium ultimum* var. *ultimum*	CBS 729.94	OOMYA1890-08	HQ708910, HQ643869
Pyt	*Pythium ultimum* var. *ultimum*	DAOM 232337	OOMYA1894-08	HQ708909, HQ643868
Pyt	*Pythium ultimum* var. *ultimum*	Lev 1441	OOMYA112-07	HQ708908, HQ643867
Pyt	*Pythium ultimum* var. *ultimum*	Lev 1442	OOMYA171-07	HQ708907, HQ643866
Pyt	*Pythium ultimum* var. *ultimum*	CBS 122650	OOMYA1409-08	HQ708905, HQ643864, HQ665103
Pyt	*Pythium ultimum* var. *ultimum*	CBS 398.51	OOMYA149-07	HQ708906, HQ643865, HQ665227
Pyt	*Pythium uncinulatum*	ADC 0108	OOMYA1684-08	HQ708986, HQ643945
Pyt	*Pythium uncinulatum**	CBS 518.77	OOMYA173-07	HQ708985, HQ643944, HQ665244
Pyt	*Pythium undulatum*	ADC 9929	OOMYA1899-08	HQ708990, HQ643949
Pyt	*Pythium undulatum*	CBS 323.47	OOMYA1116-08	HQ708989, HQ643948
Pyt	*Pythium undulatum*	Lev 1232	OOMYA250-07	HQ708988, HQ643947
Pyt	*Pythium undulatum*	CBS 157.69	OOMYA023-07	HQ708987, HQ643946, HQ665134
Pyt	*Pythium vanterpoolii*	CBS 115.77	OOMYA009-07	HQ708991, HQ643950
Pyt	*Pythium vanterpoolii*	CBS 431.91	OOMYA1400-08	HQ708992, HQ643951
Pyt	*Pythium vanterpoolii*	Lev 1536	OOMYA311-07	HQ708994, HQ643953
Pyt	*Pythium vanterpoolii**	CBS 295.37	OOMYA110-07	HQ708993, HQ643952, HQ665193
Pyt	*Pythium viniferum**	CBS 119168	OOMYA1900-08	HQ708997, HQ643956, HQ665086
Pyt	*Pythium violae*	ADC 9739	OOMYA1901-08	HQ709010, HQ643969
Pyt	*Pythium violae*	ADC 9754	OOMYA1902-08	HQ709009, HQ643968
Pyt	*Pythium violae*	ADC 9923	OOMYA1903-08	HQ709008, HQ643967
Pyt	*Pythium violae*	ADC 9924	OOMYA1904-08	HQ709007, HQ643966
Pyt	*Pythium violae*	ADC 9974	OOMYA1905-08	HQ709006, HQ643965
Pyt	*Pythium violae*	ADC 9975	OOMYA1906-08	HQ709005, HQ643964
Pyt	*Pythium violae*	BR 322	OOMYA1907-08	HQ709004, HQ643963
Pyt	*Pythium violae*	Lev 1518	OOMYA299-07	HQ709003, HQ643962
Pyt	*Pythium violae*	Lev 1519	OOMYA300-07	HQ709002, HQ643961
Pyt	*Pythium violae*	Lev 1604	OOMYA336-07	HQ709001, HQ643960
Pyt	*Pythium violae*	Lev 1605	OOMYA337-07	HQ709000, HQ643959
Pyt	*Pythium violae*	CBS 159.64	OOMYA026-07	HQ708999, HQ643958, HQ665138
Pyt	*Pythium violae*	CBS 178.86	OOMYA033-07	HQ708998, HQ643957, HQ665143
Pyt	*Pythium volutum*	CBS 699.83	OOMYA220-07	HQ709012, HQ643971, HQ665291
Pyt	*Pythium zingiberis*	CBS 217.82	OOMYA1909-08	HQ709013, HQ643972
Pyt	*Pythium zingiberis*	CBS 216.82	OOMYA050-07	HQ709014, HQ643973, HQ665155
Sap	*Saprolegnia anisospora*	CBS 110060	OOMYA1910-08	HQ709015, HQ643974
Sap	*Saprolegnia asterophora*	CBS 531.67	OOMYA1911-08	HQ709016, HQ643975, HQ665250
Sap	*Saprolegnia delica*	CBS 344.62	OOMYA1912-08	HQ709018, HQ643977
Sap	*Saprolegnia delica*	CBS 345.62	OOMYA1913-08	HQ709017, HQ643976, HQ665214
Sap	*Saprolegnia diclina*	CBS 282.38	OOMYA1915-08	HQ709020, HQ643979
Sap	*Saprolegnia diclina*	CBS 326.35	OOMYA1916-08	HQ709021, HQ643980, HQ665209
Sap	*Saprolegnia diclina*	CBS 536.67	OOMYA1917-08	HQ709019, HQ643978, HQ665254
Sap	*Saprolegnia eccentrica*	CBS 199.38	OOMYA1918-08	HQ709023, HQ643982, HQ665147
Sap	*Saprolegnia eccentrica*	CBS 211.35	OOMYA1919-08	HQ709022, HQ643981, HQ665151
Sap	*Saprolegnia eccentrica*	CBS 551.67	OOMYA1920-08	HQ709024, HQ643983, HQ665260
Sap	*Saprolegnia ferax*	BR 114	OOMYA1921-08	HQ709029, HQ643988
Sap	*Saprolegnia ferax*	CBS 283.38	OOMYA1923-08	HQ709027, HQ643986
Sap	*Saprolegnia ferax*	CBS 534.67	OOMYA1925-08	HQ709026, HQ643985
Sap	*Saprolegnia ferax*	CBS 173.42	OOMYA1922-08	HQ709025, HQ643984, HQ665142
Sap	*Saprolegnia ferax*	CBS 305.37	OOMYA1924-08	HQ709028, HQ643987, HQ665199
Sap	*Saprolegnia hypogyna*	CBS 869.72	OOMYA1927-08	HQ709030, HQ643989, HQ665304
Sap	*Saprolegnia lapponica*	CBS 284.38	OOMYA1928-08	HQ709031, HQ643990, HQ665184
Sap	*Saprolegnia litoralis*	CBS 110062	OOMYA1929-08	HQ709033, HQ643992, HQ665060
Sap	*Saprolegnia litoralis*	CBS 535.67	OOMYA1930-08	HQ709032, HQ643991, HQ665253
Sap	*Saprolegnia megasperma*	CBS 532.67	OOMYA1932-08	HQ709034, HQ643993, HQ665251
Sap	*Saprolegnia mixta*	CBS 149.65	OOMYA1933-08	HQ709035, HQ643994, HQ665127
Sap	*Saprolegnia mixta*	CBS 307.37	OOMYA1934-08	HQ709036, HQ643995, HQ665202
Sap	*Saprolegnia monilifera*	CBS 552.67	OOMYA1935-08	HQ709038, HQ643997, HQ665262
Sap	*Saprolegnia monilifera*	CBS 558.67	OOMYA1936-08	HQ709037, HQ643996, HQ665270
Sap	*Saprolegnia monoica*	CBS 539.67	OOMYA1939-08	HQ709040, HQ643999, HQ665255
Sap	*Saprolegnia monoica*	CBS 599.67	OOMYA1940-08	HQ709039, HQ643998, HQ665280

Sap	*Saprolegnia parasitica*	CBS 113187	OOMYA1941-08	HQ709046, HQ644005, HQ665074
Sap	*Saprolegnia parasitica*	CBS 223.65	OOMYA1942-08	HQ709045, HQ644004, HQ665165
Sap	*Saprolegnia parasitica*	CBS 300.32	OOMYA1943-08	HQ709044, HQ644003, HQ665196
Sap	*Saprolegnia parasitica*	CBS 302.56	OOMYA1944-08	HQ709043, HQ644002, HQ665197
Sap	*Saprolegnia parasitica*	CBS 397.34	OOMYA1945-08	HQ709042, HQ644001, HQ665226
Sap	*Saprolegnia parasitica*	CBS 540.67	OOMYA1946-08	HQ709041, HQ644000, HQ665256
Sap	*Saprolegnia rodrigueziana*	CBS 119354	OOMYA1948-08	HQ709047, HQ644006, HQ665089
Sap	*Saprolegnia* sp.	CBS 632.85	OOMYA1701-08	HQ709048, HQ644007
Sap	*Saprolegnia subterranea*	CBS 278.52	OOMYA1950-08	HQ709049, HQ644008
Sap	*Saprolegnia subterranea*	CBS 113343	OOMYA1914-08	HQ709050, HQ644009, HQ665078
Sap	*Saprolegnia terrestris*	CBS 533.67	OOMYA1952-08	HQ709051, HQ644010
Sap	*Saprolegnia terrestris*	CBS 110063	OOMYA1951-08	HQ709052, HQ644011, HQ665061
Sap	*Saprolegnia turfosa*	CBS 110065	OOMYA1953-08	HQ709055, HQ644014
Sap	*Saprolegnia turfosa*	CBS 313.81	OOMYA1954-08	HQ709054, HQ644013, HQ665203
Sap	*Saprolegnia turfosa*	CBS 327.35	OOMYA1938-08	HQ709053, HQ644012, HQ665210
Sap	*Saprolegnia unispora*	CBS 110066	OOMYA1955-08	HQ709057, HQ644016, HQ665062
Sap	*Saprolegnia unispora*	CBS 213.35	OOMYA1956-08	HQ709056, HQ644015, HQ665152
Thr	*Thraustotheca clavata*	CBS 343.33	OOMYA1957-08	HQ709059, HQ644018, HQ665213
Thr	*Thraustotheca clavata*	CBS 557.67	OOMYA1958-08	HQ709058, HQ644017, HQ665268
Thr	*Thraustotheca terrestris**	CBS 109851	OOMYA1960-08	HQ709060, HQ644019, HQ665057

Text S1A. Species resolved by COI that are indistinguishable by ITS

Phytophthora:

- *Phytophthora cambivora* and *Phytophthora alni*

Pythium:

- *Pythium catenulatum* and *Pythium rhizo-oryzae*
- *Pythium graminicola*, *Pythium periilum*, and *Pythium tardicrescens*
- *Pythium sylvaticum* and *Pythium terrestris*
- *Pythium mamillatum* and *Pythium spiculum*
- *Pythium attrantheridium* and *Pythium* sp. "*balticum*"
- *Pythium aquatile* and *Pythium sukuiense*
- *Pythium capillosum* and *Pythium flevoense*

Phytopythium:

- *Phytopythium boreale* and *Phytopythium megacarpum*
- *Phytopythium* sp. "*grandilobatum*" and *Phytopythium oedochilum*

Text S1B. Species that are indistinguishable with either COI or ITS

Phytophthora:

- *Phytophthora capsici* and *Phytophthora mexicana* (Mchau & Coffey 1995)
- *Phytophthora melonis* and *Phytophthora sinensis* (Erwin & Ribeiro 1996; Guharoy *et al.* 2006; Mirabolfathy *et al.* 2001)
- *Phytophthora arecae* and *Phytophthora palmivora*
- *Phytophthora erythroseptica* and *Phytophthora himalayensis*
- *Phytophthora nicotianae* and *Phytophthora tabaci*
- *Phytophthora parsiana* and *Phytophthora* sp. "*thermophilum*" or *Phytophthora* sp. "*lagoariana*"

In the last example, one isolate of *P. parsiana* shows conspecificity with *P.* sp. "*thermophilum*", one shows conspecificity with *P.* sp. "*lagoariana*", and the third *P. parsiana* (P21281) seems separate from the rest.

Pythium:

- *Pythium myriotylum* and *Pythium zingiberis*
- *Pythium aristosporum* and *Pythium arrhenomanes*
- *Pythium amasculinum, Pythium hydnosporum, Pythium lycopersicum,* and *Pythium ornamentatum.*
- *Pythium folliculosum* and *Pythium torulosum*
- *Pythium conidiophorum* and *Pythium salpingophorum*
- *Pythium debaryanum* and *Pythium viniferum*

- *Pythium irregulare, Pythium cryptoirregulare,* and *Pythium cylindrosporum*
- *Pythium acrogynum* and *Pythium hypogynum*
- *Pythium erinaceus* and *Pythium ornacarpum*
- *Pythium minus* and *Pythium pleroticum*
- *Pythium dimorphum* and *Pythium undulatum*

Saprolegnia:

- *Saprolegnia subterranea* and *Saprolegnia rodrigueziana*

Thraustotheca:

- *Thraustotheca clavata* and *Thraustotheca terrestris*

Text S1C. Apparent species complexes based on COI and ITS

Achlya:

- *Achlya ambisexualis, Achlya bisexualis,* and *Achlya heterosexualis*

Phytophthora:

- *Phytophthora citricola, Phytophthora inflata, Phytophthora pini,* and *Phytophthora plurivora.*
- *Phytophthora cryptogea, Phytophthora erythroseptica, Phytophthora* sp. *"kelmania",* and *Phytophthora cryptogea* f. sp. *begoniae*

Other *Phytophthora* species that could be considered a complex on their own are:

- *Phytophthora megasperma* (Hansen & Maxwell 1991)
- *Phytophthora cryptogea* (Gallegly & Hong 2008)

Pythium:

- *Pythium glomeratum* and *Pythium heterothallicum*
- *Pythium rhizosaccharum* and *Pythium takayamanum*
- *Pythium buismaniae, Pythium megalacanthum, Pythium polymastum,* and *Pythium uncinulatum.*

Other *Pythium* species that could be considered a complex on their own are:

- *Pythium irregulare* (Barr *et al.* 1997)
- *Pythium ultimum* var. *ultimum* (Barr *et al.* 1996)
- *Pythium acanthicum*
- *Pythium okanoganense*
- *Pythium violae* (epithet is currently assigned to two distinct isolates from carrot and soil that occupy different clades in the genus *Pythium*).

Phytopythium:

- *Phytopythium vexans* and *Phytopythium cucurbitacearum*

Saprolegnia

- *Saprolegnia ferax, Saprolegnia parasitica, Saprolegnia diclina, Saprolegnia lapponica, Saprolegnia mixta, Saprolegnia unispora, Saprolegnia hypogyna,* and *Saprolegnia delica*

Barr DJS, Warwick SI, Desaulniers NL (1997) Isozyme variation, morphology, and growth response to temperature in *Pythium irregulare*. *Can J Botany* **75**, 2073-2081.

Barr DJS, Warwick SI, Désaulniers NL (1996) Isozyme variation, morphology, and growth response to temperature in *Pythium ultimum*. *Can J Botany* **74**, 753-761.

Erwin DC, Ribeiro OK (1996) *Phytophthora diseases worldwide* American Phytopathological Society, St Paul.

Gallegly ME, Hong C (2008) *Phytophthora: Identifying species by morphology and DNA fingerprints* American Phytopathological Society, St Paul.

Guharoy S, Bhattacharyya S, Mukherjee SK, Mandal N, Khatua DC (2006) *Phytophthora melonis* associated with fruit and vine rot disease of pointed gourd in India as revealed by RFLP and sequencing of ITS region. *J Phytopathology* **154**, 612-615.

Hansen EM, Maxwell DP (1991) Species of the *Phytophthora megasperma* complex. *Mycologia* **83**, 376-381.

Mchau GRA, Coffey MD (1995) Evidence for the existence of two subpopulations in *Phytophthora capsici* and a redescription of the species. *Mycological Research* **99**, 89-102.

Mirabolfathy M, Cooke DEL, Duncan JM*, et al.* (2001) *Phytophthora pistaciae* sp. nov. and *P. melonis*: the principal causes of pistachio gummosis in Iran. *Mycological Research* **105**, 1166-1175.

Text S2. Additional methods

DNA extraction. For cultures from the Ristaino lab, a CTAB extraction of cultures grown in pea broth was done (Trout *et al.* 1997). For cultures grown from the personal collection of André Lévesque (Lev), mycelia from 5 -14 day old liquid cultures grown in potato dextrose broth (Difco) at room temp were removed from broth and DNA was extracted with the FastDNA® kit (BIO 101) following manufacturer's protocol. For cultures from the World Oomycete Genetic

Resource Collection (WOC), accessions were grown for 7-14 days either in clarified V8 juice or rye seed broths and were extracted using a modified protocol with the FastDNA® kit (Blair *et al.* 2008). For Plant Research International (PRI) isolates, mycelia from 8 - 14 day old liquid cultures grown in pea broth (de Cock *et al.* 1992) were harvested by vacuum filtration, freeze dried, and DNA was extracted following the protocol from the Wizard® Magnetic DNA Purification System for Food (Promega). From the obligate biotrophic downy mildews, DNA was extracted from frozen sporangia or silica gel dried infected host tissue according to the protocol of Riethmüller *et al.* (Riethmüller *et al.* 2002).

COI primer design. In order to design COI barcode primers for a broader range of oomycete genera, the 5' and middle region of COI, overlapping the commonly used barcode primers, was amplified and sequenced using eight different genera of oomycetes with a combination of FM79, FM80, FM82, FM83 and FM85 originally designed for *Phytophthora* (Martin & Tooley 2003). OomCoxI-Levup was designed to overlap LCO1490 (Folmer *et al.* 1994) and GazF1 (Saunders 2005) primers whereas Fm85mod was a modified version of Fm85 from Martin and Tooley (Martin & Tooley 2003). OomCoxI-Levlo was designed to overlap HCO2198 (Folmer *et al.* 1994) and GazR1 (Saunders 2005).

DNA amplification. For isolates from the WOC, the ITS region was amplified with either ITS1 and ITS4 (White *et al.* 1990) or ITS6 (Cooke *et al.* 2000) and ITS4. For PRI isolates, ITS1 and ITS4 were used. For Ristaino lab isolates, ITS6 and ITS4 were used. ITS5-P2 (Voglmayr & Constantinescu 2008) and ITS4 were used to amplify the complete ITS region of downy mildews. In *Basidiophora*, *Plasmopara* and *Plasmoverna* the ITS2 contained long insertions hampering sequencing of the complete ITS region, so in these cases only the ITS 1 region was

amplified with primers ITS5-P2 and 5.8S-Pr (Voglmayr & Constantinescu 2008). The LSU of downy mildews was amplified with primers LR0R (Moncalvo *et al.* 1995) and LR6-O (Riethmüller *et al.* 2002). COI of *Phytophthora* isolates from the Ristaino lab was amplified with FM 85 and the reverse complement of FM 80 (FM80RC) (Martin & Tooley 2003). For PRI isolates, COI was amplified with M13F-ligated OomCoxI-Levup (5'-CGTGTAAAACGACGGCCAGTTCAWCWMGATGGCTTTTTCAAC-3') and M13R-ligated Fm85mod (5'- CGCAGGAAACAGCTATGACCRRHWACKTGACTDATRATACCAAA-3'). PCR reaction volume for PRI isolates was 25 µL with 1 µL DNA and 24 µL Roche PCR Master mix including 5 µM each of forward and reverse primer. Thermocycler program for amplification of the ITS region in PRI isolates was: 94 ºC for 2 min followed by 35 cycles of 94 ºC for 30 s, 56 ºC for 30 s, 72 ºC for 1 min. A final extension was made at 72 ºC for 5 min. Thermocycler program for amplification of the COI region in PRI isolates was: 94 ºC for 2 min followed by 35 cycles of 94 ºC for 30 s, 55 ºC for 30 s, 72 ºC for 90 s. A final extension was made at 72 ºC for 5 min.

Sequencing amplification. Sequencing primers for downy mildews were ITS5-P2, 5.8S-Pr and ITS4 for the ITS region. For LSU, LR0R and LR6-O were used. For sequencing from Ristaino lab, FM 85 and FM80RC were used for COI and ITS4 and ITS6 were used for ITS. For PRI isolates, amplification of PCR products for sequencing was done with ABI Big Dye Terminator v3.1 in a reaction volume of 10 µL, with Big Dye Seq Mix diluted 3:5 with Seq buffer. Final concentrations of each reagent were 0.625X Sequencing buffer, 5% trehalose, 0.375X Big Dye Seq Mix, and 0.4 µM primer. Reaction volume was brought to 10 µL with sterile HPLC water and 2 µL of PCR product was added directly from initial PCR amplification with purification by

sephadex column. Thermocycler program was: 95 ºC for 3 min followed by 25 cycles of 94 ºC for 20 s, 58 ºC for 15 s, 60 ºC for 1 min. Sequencing primers for ITS were ITS1, ITS2, ITS3 and ITS4 (White *et al.* 1990). Sequencing primers for COI were M13R (5'-CAGGAAACAGCTATGAC-3'), M13F (5'-GTAAAACGACGGCCAG-3'), CoxF4N (5'-CGTGAACTAATGTTACATATAC-3') and OomCoxI-Levlo.

Cloning PCR products. In some cases, direct sequencing of PCR products from the ITS/LSU region resulted in poor sequence results due to apparent polymerase slippage at polyT or polyA regions, or due to the possibility of indels in some copies or alleles of this multicopy locus. Cloning of PCR products and sequencing directly from the cloning vectors was able to overcome this problem. PCR products were purified by mixing 5 µL of PCR product with 2 µL ExoSAP-IT® (USB), incubating at 37ºC for 15 min, followed by incubation at 80ºC for 15 min. Purified PCR products were then poly-adenylated and ligated to the pGEM®-T Easy vector (Promega). Transfection of competent cells, selection of transformants, and growth of transformant cultures were performed according to manufacturer's protocols. Isolation of plasmid DNA was performed according to Sambrook and Russel (Sambrook & Russell 2001), with omission of the optional phenol/chloroform step. Plasmids isolated from transformants were screened for inserts by PCR with T7 (5'-TAATACGACTCACTATAGGG-3') and SP6 (5'-TATTTAGGTGACACTATAG-3') plasmid primers. Program for screening PCR was: 95 ºC for 3 min followed by 40 cycles of 95 ºC for 30 s, 50 ºC for 45 s, 72 ºC for 2 min 30 s. A final extension was made at 72 ºC for 8 min.

Plasmid sequencing amplification. Inserted PCR products in isolated plasmids were amplified for sequencing with ABI Big Dye Terminator v3.1 in a reaction volume of 10 µL, with Big Dye

Seq Mix diluted 1:8 with Seq buffer. Sequencing primers were T7 and SP6. Final concentrations of each reagent were 0.875X Sequencing buffer, 0.125X Big Dye Seq Mix, 0.16 µM primer and approx 400 ng plasmid. Thermocycler program was: 95 ºC for 3 min followed by 40 cycles of 95 ºC for 30 s, 50 ºC for 30 s, 60 ºC for 4 min.

Sequencing. DNA sequences from downey mildews were generated from sequencing amplification reactions using the ABI Prism 3730*xl* Genetic analyzer.

Barr DJS, Warwick SI, Desaulniers NL (1997) Isozyme variation, morphology, and growth response to temperature in *Pythium irregulare*. *Can J Botany* **75**, 2073-2081.

Barr DJS, Warwick SI, Désaulniers NL (1996) Isozyme variation, morphology, and growth response to temperature in *Pythium ultimum*. *Can J Botany* **74**, 753-761.

Blair JE, Coffey MD, Park SY, Geiser DM, Kang S (2008) A multi-locus phylogeny for *Phytophthora* utilizing markers derived from complete genome sequences. *Fungal Genetics and Biology* **45**, 266-277.

Cooke DEL, Drenth A, Duncan JM, Wagels G, Brasier CM (2000) A molecular phylogeny of *Phytophthora* and related oomycetes. *Fungal Genetics and Biology* **30**, 17-32.

de Cock AWAM, Neuvel A, Bahnweg G, de Cock JCJM, Prell HH (1992) A comparison of morphology, pathogenicity and restriction fragment patterns of mitochondrial DNA among isolates of *Phytophthora porri* Foister. *Netherlands Journal of Plant Pathology* **98**, 277-289.

Erwin DC, Ribeiro OK (1996) *Phytophthora diseases worldwide* American Phytopathological Society, St Paul.

Folmer O, Black M, Hoeh W, Lutz R, Vrijenhoek R (1994) DNA primers for amplification of mitochondrial cytochrome c oxidase subunit I from diverse metazoan invertebrates. *Mol Mar Biol Biotechnol* **3**, 294-299.

Gallegly ME, Hong C (2008) *Phytophthora: Identifying species by morphology and DNA fingerprints* American Phytopathological Society, St Paul.

Guharoy S, Bhattacharyya S, Mukherjee SK, Mandal N, Khatua DC (2006) *Phytophthora melonis* associated with fruit and vine rot disease of pointed gourd in India as revealed by RFLP and sequencing of ITS region. *J Phytopathology* **154**, 612-615.

Hansen EM, Maxwell DP (1991) Species of the *Phytophthora megasperma* complex. *Mycologia* **83**, 376-381.

Martin FN, Tooley PW (2003) Phylogenetic relationships among *Phytophthora* species inferred from sequence analysis of mitochondrially encoded cytochrome oxidase I and II genes. *Mycologia* **95**, 269-284.

Mchau GRA, Coffey MD (1995) Evidence for the existence of two subpopulations in *Phytophthora capsici* and a redescription of the species. *Mycological Research* **99**, 89-102.
Mirabolfathy M, Cooke DEL, Duncan JM*, et al.* (2001) *Phytophthora pistaciae* sp. nov. and *P. melonis*: the principal causes of pistachio gummosis in Iran. *Mycological Research* **105**, 1166-1175.
Moncalvo JM, Wang HH, Hseu RS (1995) Phylogenetic relationships in *Ganoderma* inferred from the internal transcribed spacers and 25S ribosomal DNA sequences. *Mycologia* **87**, 223-238.
Riethmüller A, Voglmayr H, Goker M, Weiß M, Oberwinkler F (2002) Phylogenetic relationships of the downy mildews (Peronosporales) and related groups based on nuclear large subunit ribosomal DNA sequences. *Mycologia* **94**, 834-849.
Sambrook J, Russell DW (2001) Preparation of plasmid DNA by alkaline lysis with SDS: Minipreparation. In: *Molecular Cloning: a laboratory manual, third edition* (eds. Sambrook J, Russell DW), pp. 1.32-31.34. Cold Spring Harbor Laboratory Press, Cold Spring Harbor, NY.
Saunders GW (2005) Applying DNA barcoding to red macroalgae: a preliminary appraisal holds promise for future applications. *Philosophical Transactions of the Royal Society of London B Biological Sciences* **360**, 1879-1888.
Trout CL, Ristaino JB, Madritch M, Wangsomboondee T (1997) Rapid detection of *Phytophthora infestans* in late blight infected tissue of potato and tomato using PCR. *Plant Disease* **81**, 1042-1048.
Voglmayr H, Constantinescu O (2008) Revision and reclassification of three *Plasmopara* species based on morphological and molecular phylogenetic data. *Mycological Research* **112**, 487-501.
White TJ, Bruns T, Lee S, Taylor J (1990) Amplification and direct sequencing of fungal ribosomal RNA genes for phylogenetics. In: *PCR Protocols: a guide to methods and applications* (eds. Innis MA, Gelfand DH, Sninsky JJ, White TJ), pp. 315-322. Academic Press, San Diego.

Fig. S1. Full UPGMA trees for each marker showing all isolates with scaled branch lengths and bootstrap values. The COI tree spans pages 1 – 10, the ITS tree spans pages 11 – 20, and the LSU tree spans pages 21 – 23.

Legend. The supporting information consists of one Table (Table S1), two text sections (Text S1 and Text S2) referring to taxonomy and supplementary methods respectively, and one figure (Figure S1).

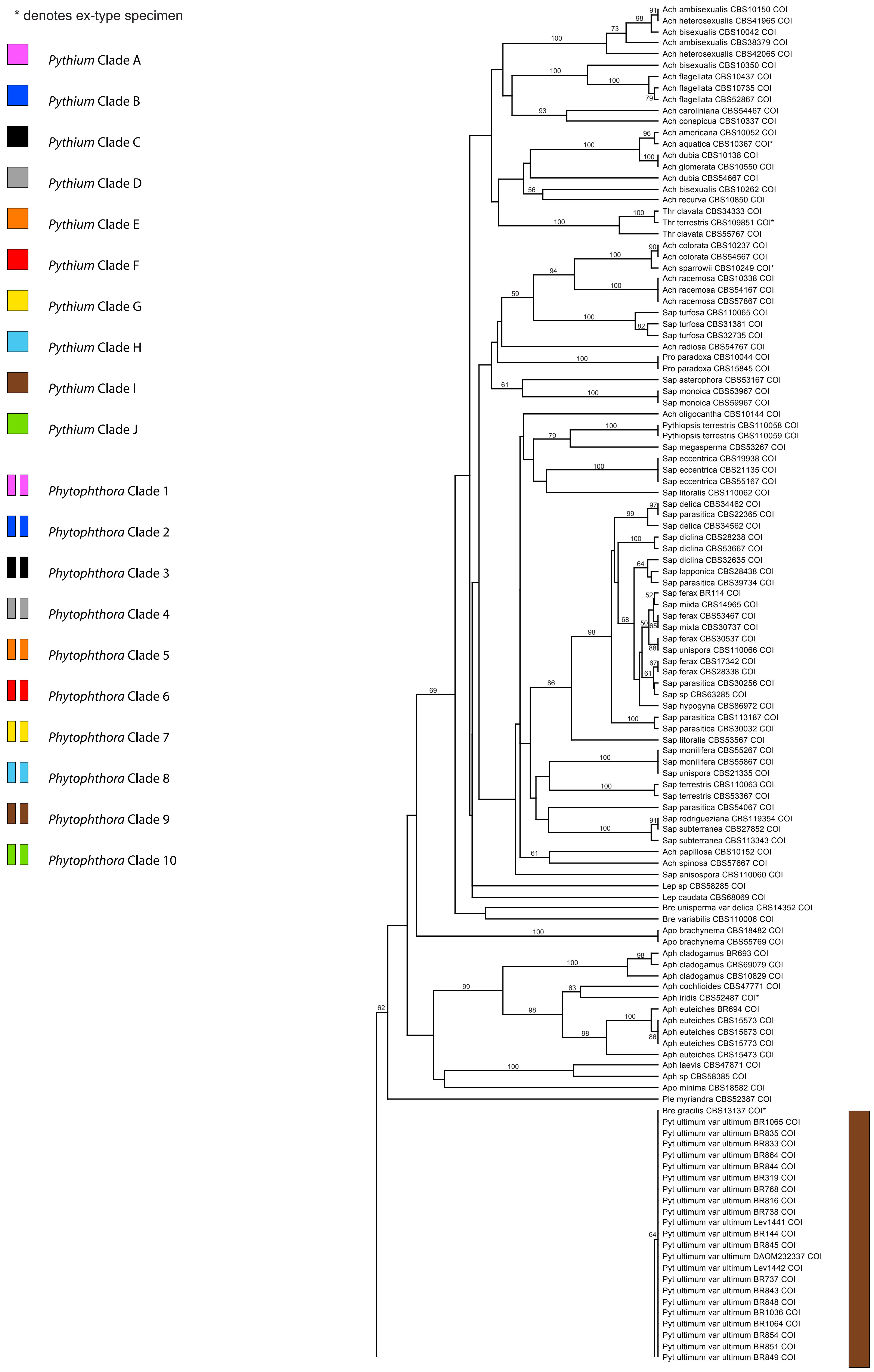

* denotes ex-type specimen
Pythium Clade A
Pythium Clade B
Pythium Clade C
Pythium Clade D
Pythium Clade E
Pythium Clade F
Pythium Clade G
Pythium Clade H
Pythium Clade I
Pythium Clade J
Phytophthora Clade 1
Phytophthora Clade 2
Phytophthora Clade 3
Phytophthora Clade 4
Phytophthora Clade 5
Phytophthora Clade 6
Phytophthora Clade 7
Phytophthora Clade 8
Phytophthora Clade 9
Phytophthora Clade 10
Ach ambisexualis CBS10150 COI
Ach heterosexualis CBS41965 COI
Ach bisexualis CBS10042 COI
Ach ambisexualis CBS38379 COI
Ach heterosexualis CBS42065 COI
Ach bisexualis CBS10350 COI
Ach flagellata CBS10437 COI
Ach flagellata CBS10735 COI
Ach flagellata CBS52867 COI
Ach caroliniana CBS54467 COI
Ach conspicua CBS10337 COI
Ach americana CBS10052 COI
Ach aquatica CBS10367 COI*
Ach dubia CBS10138 COI
Ach glomerata CBS10550 COI
Ach dubia CBS54667 COI
Ach bisexualis CBS10262 COI
Ach recurva CBS10850 COI
Thr clavata CBS34333 COI
Thr terrestris CBS109851 COI*
Thr clavata CBS55767 COI
Ach colorata CBS10237 COI
Ach colorata CBS54567 COI
Ach sparrowii CBS10249 COI*
Ach racemosa CBS10338 COI
Ach racemosa CBS54167 COI
Ach racemosa CBS57867 COI
Sap turfosa CBS110065 COI
Sap turfosa CBS31381 COI
Sap turfosa CBS32735 COI
Ach radiosa CBS54767 COI
Pro paradoxa CBS10044 COI
Pro paradoxa CBS15845 COI
Sap asterophora CBS53167 COI
Sap monoica CBS53967 COI
Sap monoica CBS59967 COI
Ach oligocantha CBS10144 COI
Pythiopsis terrestris CBS110058 COI
Pythiopsis terrestris CBS110059 COI
Sap megasperma CBS53267 COI
Sap eccentrica CBS19938 COI
Sap eccentrica CBS21135 COI
Sap eccentrica CBS55167 COI
Sap litoralis CBS110062 COI
Sap delica CBS34462 COI
Sap parasitica CBS22365 COI
Sap delica CBS34562 COI
Sap diclina CBS28238 COI
Sap diclina CBS53667 COI
Sap diclina CBS32635 COI
Sap lapponica CBS28438 COI
Sap parasitica CBS39734 COI
Sap ferax BR114 COI
Sap mixta CBS14965 COI
Sap ferax CBS53467 COI
Sap mixta CBS30737 COI
Sap ferax CBS30537 COI
Sap unispora CBS110066 COI
Sap ferax CBS17342 COI
Sap ferax CBS28338 COI
Sap parasitica CBS30256 COI
Sap sp CBS63285 COI
Sap hypogyna CBS86972 COI
Sap parasitica CBS113187 COI
Sap parasitica CBS30032 COI
Sap litoralis CBS53567 COI
Sap monilifera CBS55267 COI
Sap monilifera CBS55867 COI
Sap unispora CBS21335 COI
Sap terrestris CBS110063 COI
Sap terrestris CBS53367 COI
Sap parasitica CBS54067 COI
Sap rodrigueziana CBS119354 COI
Sap subterranea CBS27852 COI
Sap subterranea CBS113343 COI
Ach papillosa CBS10152 COI
Ach spinosa CBS57667 COI
Sap anisospora CBS110060 COI
Lep sp CBS58285 COI
Lep caudata CBS68069 COI
Bre unisperma var delica CBS14352 COI
Bre variabilis CBS110006 COI
Apo brachynema CBS18482 COI
Apo brachynema CBS55769 COI
Aph cladogamus BR693 COI
Aph cladogamus CBS69079 COI
Aph cladogamus CBS10829 COI
Aph cochlioides CBS47771 COI
Aph iridis CBS52487 COI*
Aph euteiches BR694 COI
Aph euteiches CBS15573 COI
Aph euteiches CBS15673 COI
Aph euteiches CBS15773 COI
Aph euteiches CBS15473 COI
Aph laevis CBS47871 COI
Aph sp CBS58385 COI
Apo minima CBS18582 COI
Ple myriandra CBS52387 COI
Bre gracilis CBS13137 COI*
Pyt ultimum var ultimum BR1065 COI
Pyt ultimum var ultimum BR835 COI
Pyt ultimum var ultimum BR833 COI
Pyt ultimum var ultimum BR864 COI
Pyt ultimum var ultimum BR844 COI
Pyt ultimum var ultimum BR319 COI
Pyt ultimum var ultimum BR768 COI
Pyt ultimum var ultimum BR816 COI
Pyt ultimum var ultimum BR738 COI
Pyt ultimum var ultimum Lev1441 COI
Pyt ultimum var ultimum BR144 COI
Pyt ultimum var ultimum BR845 COI
Pyt ultimum var ultimum DAOM232337 COI
Pyt ultimum var ultimum Lev1442 COI
Pyt ultimum var ultimum BR737 COI
Pyt ultimum var ultimum BR843 COI
Pyt ultimum var ultimum BR848 COI
Pyt ultimum var ultimum BR1036 COI
Pyt ultimum var ultimum BR1064 COI
Pyt ultimum var ultimum BR854 COI
Pyt ultimum var ultimum BR851 COI
Pyt ultimum var ultimum BR849 COI

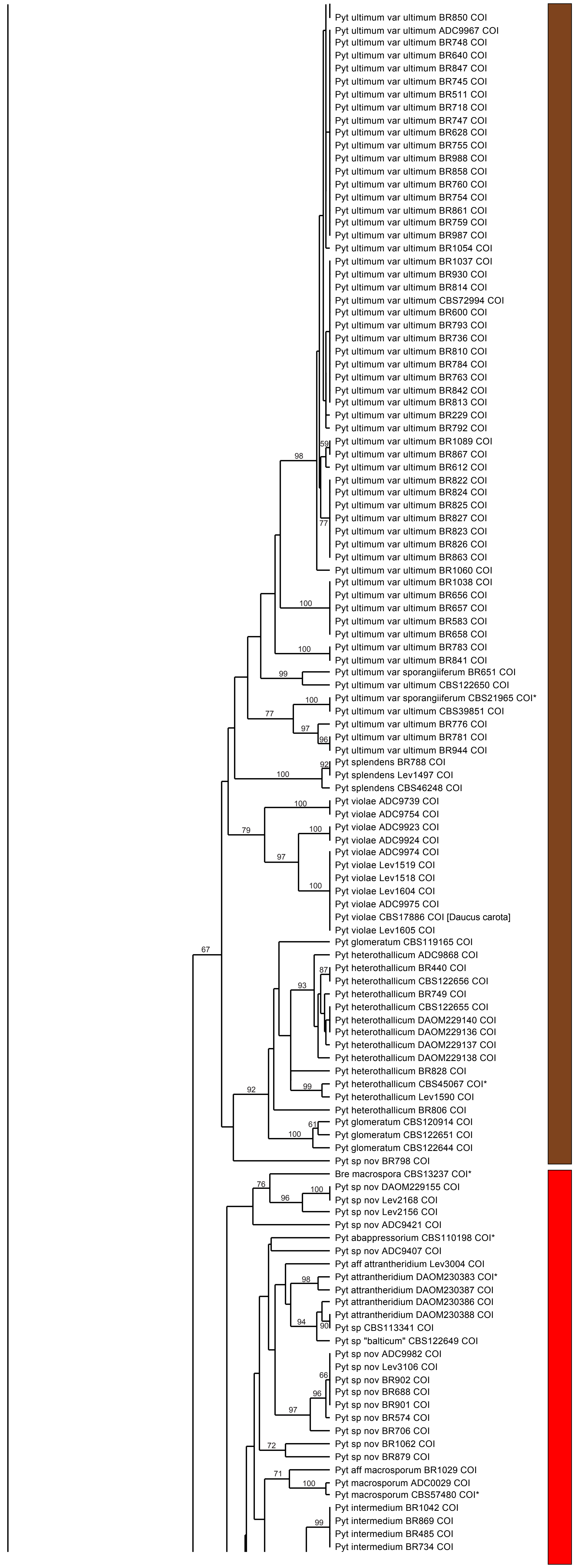
Pyt ultimum var ultimum BR850 COI
Pyt ultimum var ultimum ADC9967 COI
Pyt ultimum var ultimum BR748 COI
Pyt ultimum var ultimum BR640 COI
Pyt ultimum var ultimum BR847 COI
Pyt ultimum var ultimum BR745 COI
Pyt ultimum var ultimum BR511 COI
Pyt ultimum var ultimum BR718 COI
Pyt ultimum var ultimum BR747 COI
Pyt ultimum var ultimum BR628 COI
Pyt ultimum var ultimum BR755 COI
Pyt ultimum var ultimum BR988 COI
Pyt ultimum var ultimum BR858 COI
Pyt ultimum var ultimum BR760 COI
Pyt ultimum var ultimum BR754 COI
Pyt ultimum var ultimum BR861 COI
Pyt ultimum var ultimum BR759 COI
Pyt ultimum var ultimum BR987 COI
Pyt ultimum var ultimum BR1054 COI
Pyt ultimum var ultimum BR1037 COI
Pyt ultimum var ultimum BR930 COI
Pyt ultimum var ultimum BR814 COI
Pyt ultimum var ultimum CBS72994 COI
Pyt ultimum var ultimum BR600 COI
Pyt ultimum var ultimum BR793 COI
Pyt ultimum var ultimum BR736 COI
Pyt ultimum var ultimum BR810 COI
Pyt ultimum var ultimum BR784 COI
Pyt ultimum var ultimum BR763 COI
Pyt ultimum var ultimum BR842 COI
Pyt ultimum var ultimum BR813 COI
Pyt ultimum var ultimum BR229 COI
Pyt ultimum var ultimum BR792 COI
Pyt ultimum var ultimum BR1089 COI
Pyt ultimum var ultimum BR867 COI
Pyt ultimum var ultimum BR612 COI
Pyt ultimum var ultimum BR822 COI
Pyt ultimum var ultimum BR824 COI
Pyt ultimum var ultimum BR825 COI
Pyt ultimum var ultimum BR827 COI
Pyt ultimum var ultimum BR823 COI
Pyt ultimum var ultimum BR826 COI
Pyt ultimum var ultimum BR863 COI
Pyt ultimum var ultimum BR1060 COI
Pyt ultimum var ultimum BR1038 COI
Pyt ultimum var ultimum BR656 COI
Pyt ultimum var ultimum BR657 COI
Pyt ultimum var ultimum BR583 COI
Pyt ultimum var ultimum BR658 COI
Pyt ultimum var ultimum BR783 COI
Pyt ultimum var ultimum BR841 COI
Pyt ultimum var sporangiiferum BR651 COI
Pyt ultimum var ultimum CBS122650 COI
Pyt ultimum var sporangiiferum CBS21965 COI*
Pyt ultimum var ultimum CBS39851 COI
Pyt ultimum var ultimum BR776 COI
Pyt ultimum var ultimum BR781 COI
Pyt ultimum var ultimum BR944 COI
Pyt splendens BR788 COI
Pyt splendens Lev1497 COI
Pyt splendens CBS46248 COI
Pyt violae ADC9739 COI
Pyt violae ADC9754 COI
Pyt violae ADC9923 COI
Pyt violae ADC9924 COI
Pyt violae ADC9974 COI
Pyt violae Lev1519 COI
Pyt violae Lev1518 COI
Pyt violae Lev1604 COI
Pyt violae ADC9975 COI
Pyt violae CBS17886 COI [Daucus carota]
Pyt violae Lev1605 COI
Pyt glomeratum CBS119165 COI
Pyt heterothallicum ADC9868 COI
Pyt heterothallicum BR440 COI
Pyt heterothallicum CBS122656 COI
Pyt heterothallicum BR749 COI
Pyt heterothallicum CBS122655 COI
Pyt heterothallicum DAOM229140 COI
Pyt heterothallicum DAOM229136 COI
Pyt heterothallicum DAOM229137 COI
Pyt heterothallicum DAOM229138 COI
Pyt heterothallicum BR828 COI
Pyt heterothallicum CBS45067 COI*
Pyt heterothallicum Lev1590 COI
Pyt heterothallicum BR806 COI
Pyt glomeratum CBS120914 COI
Pyt glomeratum CBS122651 COI
Pyt glomeratum CBS122644 COI
Pyt sp nov BR798 COI
Bre macrospora CBS13237 COI*
Pyt sp nov DAOM229155 COI
Pyt sp nov Lev2168 COI
Pyt sp nov Lev2156 COI
Pyt sp nov ADC9421 COI
Pyt abappressorium CBS110198 COI*
Pyt sp nov ADC9407 COI
Pyt aff attrantheridium Lev3004 COI
Pyt attrantheridium DAOM230383 COI*
Pyt attrantheridium DAOM230387 COI
Pyt attrantheridium DAOM230386 COI
Pyt attrantheridium DAOM230388 COI
Pyt sp CBS113341 COI
Pyt sp "balticum" CBS122649 COI
Pyt sp nov ADC9982 COI
Pyt sp nov Lev3106 COI
Pyt sp nov BR902 COI
Pyt sp nov BR688 COI
Pyt sp nov BR901 COI
Pyt sp nov BR574 COI
Pyt sp nov BR706 COI
Pyt sp nov BR1062 COI
Pyt sp nov BR879 COI
Pyt aff macrosporum BR1029 COI
Pyt macrosporum ADC0029 COI
Pyt macrosporum CBS57480 COI*
Pyt intermedium BR1042 COI
Pyt intermedium BR869 COI
Pyt intermedium BR485 COI
Pyt intermedium BR734 COI

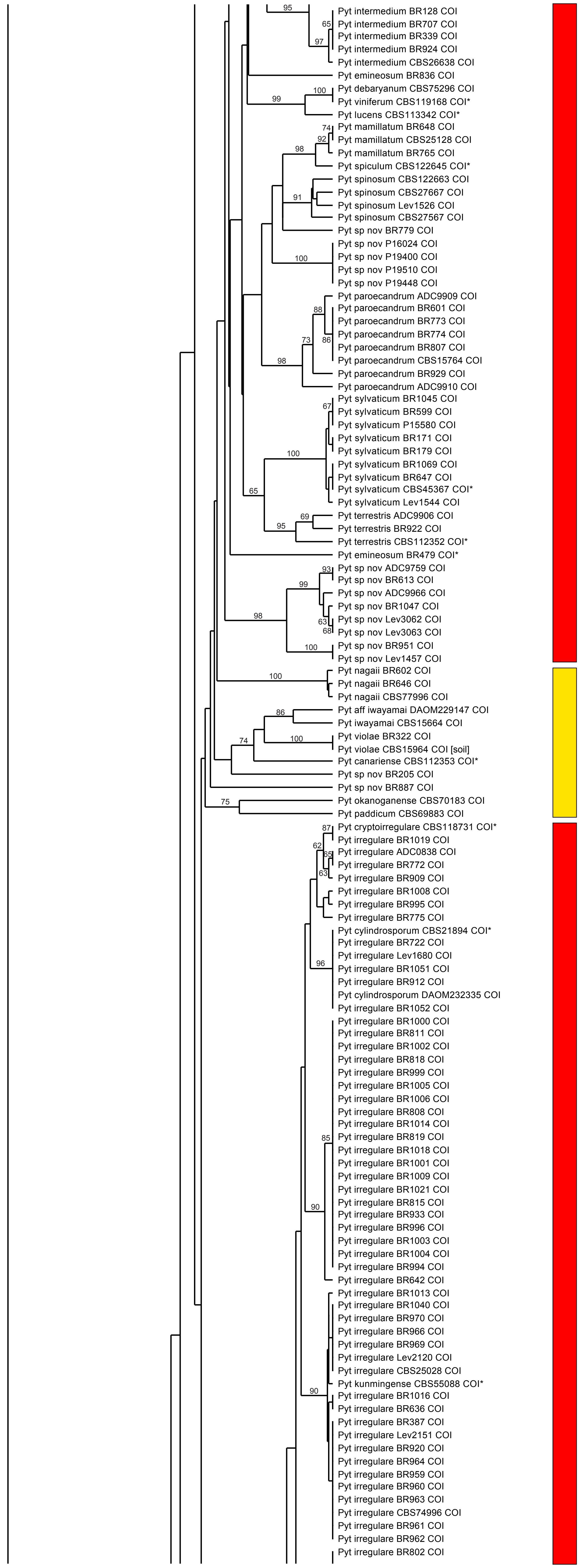

Pyt intermedium BR128 COI
Pyt intermedium BR707 COI
Pyt intermedium BR339 COI
Pyt intermedium BR924 COI
Pyt intermedium CBS26638 COI
Pyt emineosum BR836 COI
Pyt debaryanum CBS75296 COI
Pyt viniferum CBS119168 COI*
Pyt lucens CBS113342 COI*
Pyt mamillatum BR648 COI
Pyt mamillatum CBS25128 COI
Pyt mamillatum BR765 COI
Pyt spiculum CBS122645 COI*
Pyt spinosum CBS122663 COI
Pyt spinosum CBS27667 COI
Pyt spinosum Lev1526 COI
Pyt spinosum CBS27567 COI
Pyt sp nov BR779 COI
Pyt sp nov P16024 COI
Pyt sp nov P19400 COI
Pyt sp nov P19510 COI
Pyt sp nov P19448 COI
Pyt paroecandrum ADC9909 COI
Pyt paroecandrum BR601 COI
Pyt paroecandrum BR773 COI
Pyt paroecandrum BR774 COI
Pyt paroecandrum BR807 COI
Pyt paroecandrum CBS15764 COI
Pyt paroecandrum BR929 COI
Pyt paroecandrum ADC9910 COI
Pyt sylvaticum BR1045 COI
Pyt sylvaticum BR599 COI
Pyt sylvaticum P15580 COI
Pyt sylvaticum BR171 COI
Pyt sylvaticum BR179 COI
Pyt sylvaticum BR1069 COI
Pyt sylvaticum BR647 COI
Pyt sylvaticum CBS45367 COI*
Pyt sylvaticum Lev1544 COI
Pyt terrestris ADC9906 COI
Pyt terrestris BR922 COI
Pyt terrestris CBS112352 COI*
Pyt emineosum BR479 COI*
Pyt sp nov ADC9759 COI
Pyt sp nov BR613 COI
Pyt sp nov ADC9966 COI
Pyt sp nov BR1047 COI
Pyt sp nov Lev3062 COI
Pyt sp nov Lev3063 COI
Pyt sp nov BR951 COI
Pyt sp nov Lev1457 COI
Pyt nagaii BR602 COI
Pyt nagaii BR646 COI
Pyt nagaii CBS77996 COI
Pyt aff iwayamai DAOM229147 COI
Pyt iwayamai CBS15664 COI
Pyt violae BR322 COI
Pyt violae CBS15964 COI [soil]
Pyt canariense CBS112353 COI*
Pyt sp nov BR205 COI
Pyt sp nov BR887 COI
Pyt okanoganense CBS70183 COI
Pyt paddicum CBS69883 COI
Pyt cryptoirregulare CBS118731 COI*
Pyt irregulare BR1019 COI
Pyt irregulare ADC0838 COI
Pyt irregulare BR772 COI
Pyt irregulare BR909 COI
Pyt irregulare BR1008 COI
Pyt irregulare BR995 COI
Pyt irregulare BR775 COI
Pyt cylindrosporum CBS21894 COI*
Pyt irregulare BR722 COI
Pyt irregulare Lev1680 COI
Pyt irregulare BR1051 COI
Pyt irregulare BR912 COI
Pyt cylindrosporum DAOM232335 COI
Pyt irregulare BR1052 COI
Pyt irregulare BR1000 COI
Pyt irregulare BR811 COI
Pyt irregulare BR1002 COI
Pyt irregulare BR818 COI
Pyt irregulare BR999 COI
Pyt irregulare BR1005 COI
Pyt irregulare BR1006 COI
Pyt irregulare BR808 COI
Pyt irregulare BR1014 COI
Pyt irregulare BR819 COI
Pyt irregulare BR1018 COI
Pyt irregulare BR1001 COI
Pyt irregulare BR1009 COI
Pyt irregulare BR1021 COI
Pyt irregulare BR815 COI
Pyt irregulare BR933 COI
Pyt irregulare BR996 COI
Pyt irregulare BR1003 COI
Pyt irregulare BR1004 COI
Pyt irregulare BR994 COI
Pyt irregulare BR642 COI
Pyt irregulare BR1013 COI
Pyt irregulare BR1040 COI
Pyt irregulare BR970 COI
Pyt irregulare BR966 COI
Pyt irregulare BR969 COI
Pyt irregulare Lev2120 COI
Pyt irregulare CBS25028 COI
Pyt kunmingense CBS55088 COI*
Pyt irregulare BR1016 COI
Pyt irregulare BR636 COI
Pyt irregulare BR387 COI
Pyt irregulare Lev2151 COI
Pyt irregulare BR920 COI
Pyt irregulare BR964 COI
Pyt irregulare BR959 COI
Pyt irregulare BR960 COI
Pyt irregulare BR963 COI
Pyt irregulare CBS74996 COI
Pyt irregulare BR961 COI
Pyt irregulare BR962 COI
Pyt irregulare BR802 COI

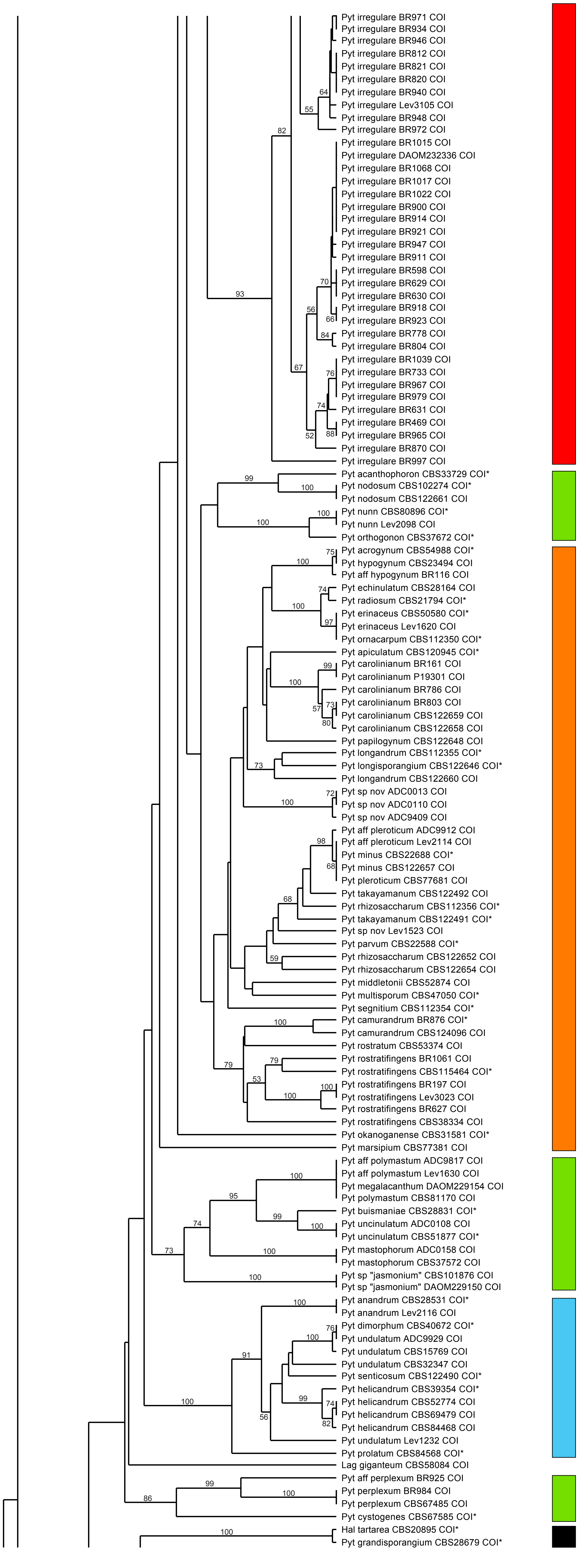

Pyt irregulare BR971 COI
Pyt irregulare BR934 COI
Pyt irregulare BR946 COI
Pyt irregulare BR812 COI
Pyt irregulare BR821 COI
Pyt irregulare BR820 COI
Pyt irregulare BR940 COI
Pyt irregulare Lev3105 COI
Pyt irregulare BR948 COI
Pyt irregulare BR972 COI
Pyt irregulare BR1015 COI
Pyt irregulare DAOM232336 COI
Pyt irregulare BR1068 COI
Pyt irregulare BR1017 COI
Pyt irregulare BR1022 COI
Pyt irregulare BR900 COI
Pyt irregulare BR914 COI
Pyt irregulare BR921 COI
Pyt irregulare BR947 COI
Pyt irregulare BR911 COI
Pyt irregulare BR598 COI
Pyt irregulare BR629 COI
Pyt irregulare BR630 COI
Pyt irregulare BR918 COI
Pyt irregulare BR923 COI
Pyt irregulare BR778 COI
Pyt irregulare BR804 COI
Pyt irregulare BR1039 COI
Pyt irregulare BR733 COI
Pyt irregulare BR967 COI
Pyt irregulare BR979 COI
Pyt irregulare BR631 COI
Pyt irregulare BR469 COI
Pyt irregulare BR965 COI
Pyt irregulare BR870 COI
Pyt irregulare BR997 COI
Pyt acanthophoron CBS33729 COI*
Pyt nodosum CBS102274 COI*
Pyt nodosum CBS122661 COI
Pyt nunn CBS80896 COI*
Pyt nunn Lev2098 COI
Pyt orthogonon CBS37672 COI*
Pyt acrogynum CBS54988 COI*
Pyt hypogynum CBS23494 COI
Pyt aff hypogynum BR116 COI
Pyt echinulatum CBS28164 COI
Pyt radiosum CBS21794 COI*
Pyt erinaceus CBS50580 COI*
Pyt erinaceus Lev1620 COI
Pyt ornacarpum CBS112350 COI*
Pyt apiculatum CBS120945 COI*
Pyt carolinianum BR161 COI
Pyt carolinianum P19301 COI
Pyt carolinianum BR786 COI
Pyt carolinianum BR803 COI
Pyt carolinianum CBS122659 COI
Pyt carolinianum CBS122658 COI
Pyt papilogynum CBS122648 COI
Pyt longandrum CBS112355 COI*
Pyt longisporangium CBS122646 COI*
Pyt longandrum CBS122660 COI
Pyt sp nov ADC0013 COI
Pyt sp nov ADC0110 COI
Pyt sp nov ADC9409 COI
Pyt aff pleroticum ADC9912 COI
Pyt aff pleroticum Lev2114 COI
Pyt minus CBS22688 COI*
Pyt minus CBS122657 COI
Pyt pleroticum CBS77681 COI
Pyt takayamanum CBS122492 COI
Pyt rhizosaccharum CBS112356 COI*
Pyt takayamanum CBS122491 COI*
Pyt sp nov Lev1523 COI
Pyt parvum CBS22588 COI*
Pyt rhizosaccharum CBS122652 COI
Pyt rhizosaccharum CBS122654 COI
Pyt middletonii CBS52874 COI
Pyt multisporum CBS47050 COI*
Pyt segnitium CBS112354 COI*
Pyt camurandrum BR876 COI*
Pyt camurandrum CBS124096 COI
Pyt rostratum CBS53374 COI
Pyt rostratifingens BR1061 COI
Pyt rostratifingens CBS115464 COI*
Pyt rostratifingens BR197 COI
Pyt rostratifingens Lev3023 COI
Pyt rostratifingens BR627 COI
Pyt rostratifingens CBS38334 COI
Pyt okanoganense CBS31581 COI*
Pyt marsipium CBS77381 COI
Pyt aff polymastum ADC9817 COI
Pyt aff polymastum Lev1630 COI
Pyt megalacanthum DAOM229154 COI
Pyt polymastum CBS81170 COI
Pyt buismaniae CBS28831 COI*
Pyt uncinulatum ADC0108 COI
Pyt uncinulatum CBS51877 COI*
Pyt mastophorum ADC0158 COI
Pyt mastophorum CBS37572 COI
Pyt sp "jasmonium" CBS101876 COI
Pyt sp "jasmonium" DAOM229150 COI
Pyt anandrum CBS28531 COI*
Pyt anandrum Lev2116 COI
Pyt dimorphum CBS40672 COI*
Pyt undulatum ADC9929 COI
Pyt undulatum CBS15769 COI
Pyt undulatum CBS32347 COI
Pyt senticosum CBS122490 COI*
Pyt helicandrum CBS39354 COI*
Pyt helicandrum CBS52774 COI
Pyt helicandrum CBS69479 COI
Pyt helicandrum CBS84468 COI
Pyt undulatum Lev1232 COI
Pyt prolatum CBS84568 COI*
Lag giganteum CBS58084 COI
Pyt aff perplexum BR925 COI
Pyt perplexum BR984 COI
Pyt perplexum CBS67485 COI
Pyt cystogenes CBS67585 COI*
Hal tartarea CBS20895 COI*
Pyt grandisporangium CBS28679 COI*

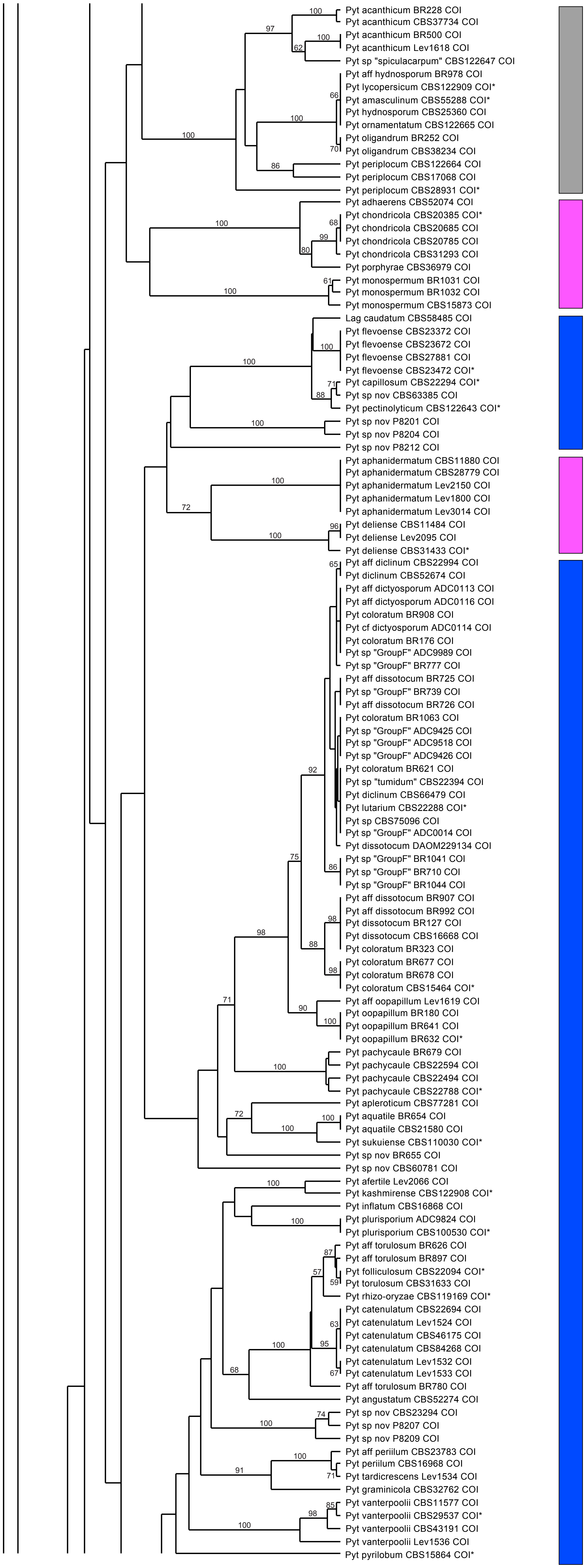

Pyt acanthicum BR228 COI
Pyt acanthicum CBS37734 COI
Pyt acanthicum BR500 COI
Pyt acanthicum Lev1618 COI
Pyt sp "spiculacarpum" CBS122647 COI
Pyt aff hydnosporum BR978 COI
Pyt lycopersicum CBS122909 COI*
Pyt amasculinum CBS55288 COI*
Pyt hydnosporum CBS25360 COI
Pyt ornamentatum CBS122665 COI
Pyt oligandrum BR252 COI
Pyt oligandrum CBS38234 COI
Pyt periplocum CBS122664 COI
Pyt periplocum CBS17068 COI
Pyt periplocum CBS28931 COI*
Pyt adhaerens CBS52074 COI
Pyt chondricola CBS20385 COI*
Pyt chondricola CBS20685 COI
Pyt chondricola CBS20785 COI
Pyt chondricola CBS31293 COI
Pyt porphyrae CBS36979 COI
Pyt monospermum BR1031 COI
Pyt monospermum BR1032 COI
Pyt monospermum CBS15873 COI
Lag caudatum CBS58485 COI
Pyt flevoense CBS23372 COI
Pyt flevoense CBS23672 COI
Pyt flevoense CBS27881 COI
Pyt flevoense CBS23472 COI*
Pyt capillosum CBS22294 COI*
Pyt sp nov CBS63385 COI
Pyt pectinolyticum CBS122643 COI*
Pyt sp nov P8201 COI
Pyt sp nov P8204 COI
Pyt sp nov P8212 COI
Pyt aphanidermatum CBS11880 COI
Pyt aphanidermatum CBS28779 COI
Pyt aphanidermatum Lev2150 COI
Pyt aphanidermatum Lev1800 COI
Pyt aphanidermatum Lev3014 COI
Pyt deliense CBS11484 COI
Pyt deliense Lev2095 COI
Pyt deliense CBS31433 COI*
Pyt aff diclinum CBS22994 COI
Pyt diclinum CBS52674 COI
Pyt aff dictyosporum ADC0113 COI
Pyt aff dictyosporum ADC0116 COI
Pyt coloratum BR908 COI
Pyt cf dictyosporum ADC0114 COI
Pyt coloratum BR176 COI
Pyt sp "GroupF" ADC9989 COI
Pyt sp "GroupF" BR777 COI
Pyt aff dissotocum BR725 COI
Pyt sp "GroupF" BR739 COI
Pyt aff dissotocum BR726 COI
Pyt coloratum BR1063 COI
Pyt sp "GroupF" ADC9425 COI
Pyt sp "GroupF" ADC9518 COI
Pyt sp "GroupF" ADC9426 COI
Pyt coloratum BR621 COI
Pyt sp "tumidum" CBS22394 COI
Pyt diclinum CBS66479 COI
Pyt lutarium CBS22288 COI*
Pyt sp CBS75096 COI
Pyt sp "GroupF" ADC0014 COI
Pyt dissotocum DAOM229134 COI
Pyt sp "GroupF" BR1041 COI
Pyt sp "GroupF" BR710 COI
Pyt sp "GroupF" BR1044 COI
Pyt aff dissotocum BR907 COI
Pyt aff dissotocum BR992 COI
Pyt dissotocum BR127 COI
Pyt dissotocum CBS16668 COI
Pyt coloratum BR323 COI
Pyt coloratum BR677 COI
Pyt coloratum BR678 COI
Pyt coloratum CBS15464 COI*
Pyt aff oopapillum Lev1619 COI
Pyt oopapillum BR180 COI
Pyt oopapillum BR641 COI
Pyt oopapillum BR632 COI*
Pyt pachycaule BR679 COI
Pyt pachycaule CBS22594 COI
Pyt pachycaule CBS22494 COI
Pyt pachycaule CBS22788 COI*
Pyt apleroticum CBS77281 COI
Pyt aquatile BR654 COI
Pyt aquatile CBS21580 COI
Pyt sukuiense CBS110030 COI*
Pyt sp nov BR655 COI
Pyt sp nov CBS60781 COI
Pyt afertile Lev2066 COI
Pyt kashmirense CBS122908 COI*
Pyt inflatum CBS16868 COI
Pyt plurisporium ADC9824 COI
Pyt plurisporium CBS100530 COI*
Pyt aff torulosum BR626 COI
Pyt aff torulosum BR897 COI
Pyt folliculosum CBS22094 COI*
Pyt torulosum CBS31633 COI
Pyt rhizo-oryzae CBS119169 COI*
Pyt catenulatum CBS22694 COI
Pyt catenulatum Lev1524 COI
Pyt catenulatum CBS46175 COI
Pyt catenulatum CBS84268 COI
Pyt catenulatum Lev1532 COI
Pyt catenulatum Lev1533 COI
Pyt aff torulosum BR780 COI
Pyt angustatum CBS52274 COI
Pyt sp nov CBS23294 COI
Pyt sp nov P8207 COI
Pyt sp nov P8209 COI
Pyt aff periilum CBS23783 COI
Pyt periilum CBS16968 COI
Pyt tardicrescens Lev1534 COI
Pyt graminicola CBS32762 COI
Pyt vanterpoolii CBS11577 COI
Pyt vanterpoolii CBS29537 COI*
Pyt vanterpoolii CBS43191 COI
Pyt vanterpoolii Lev1536 COI
Pyt pyrilobum CBS15864 COI*

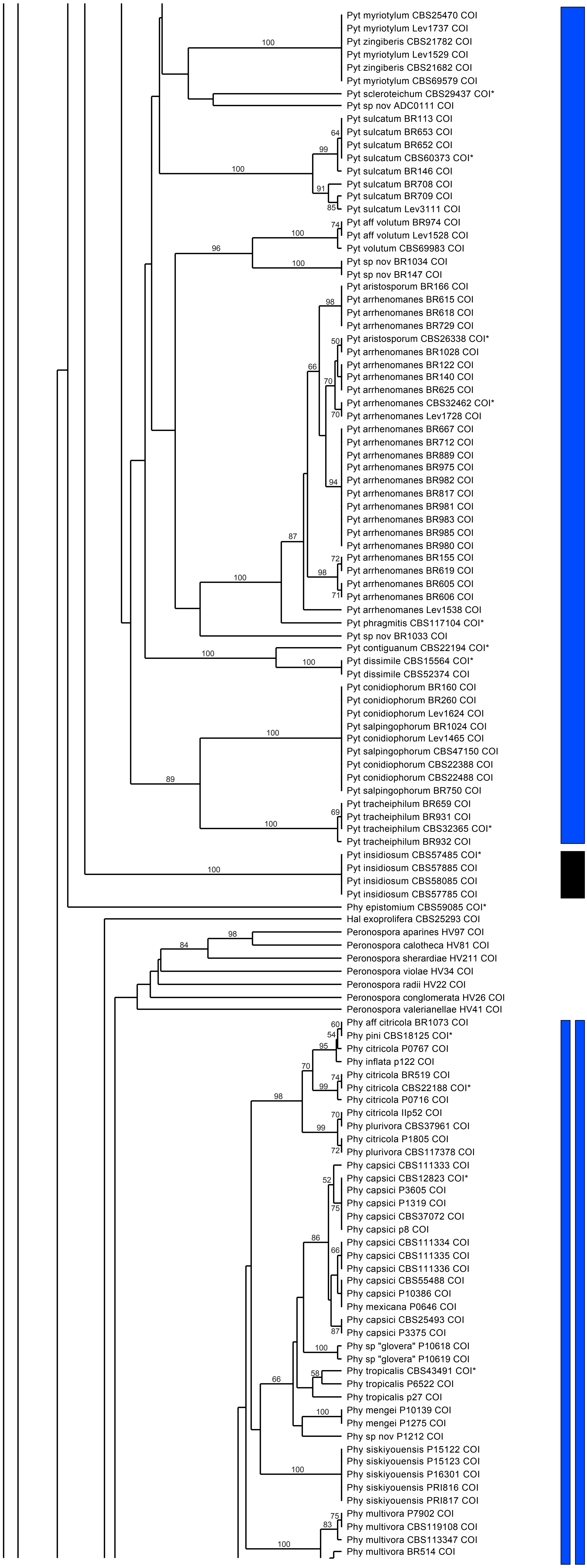
Pyt myriotylum CBS25470 COI
Pyt myriotylum Lev1737 COI
Pyt zingiberis CBS21782 COI
Pyt myriotylum Lev1529 COI
Pyt zingiberis CBS21682 COI
Pyt myriotylum CBS69579 COI
Pyt scleroteichum CBS29437 COI*
Pyt sp nov ADC0111 COI
Pyt sulcatum BR113 COI
Pyt sulcatum BR653 COI
Pyt sulcatum BR652 COI
Pyt sulcatum CBS60373 COI*
Pyt sulcatum BR146 COI
Pyt sulcatum BR708 COI
Pyt sulcatum BR709 COI
Pyt sulcatum Lev3111 COI
Pyt aff volutum BR974 COI
Pyt aff volutum Lev1528 COI
Pyt volutum CBS69983 COI
Pyt sp nov BR1034 COI
Pyt sp nov BR147 COI
Pyt aristosporum BR166 COI
Pyt arrhenomanes BR615 COI
Pyt arrhenomanes BR618 COI
Pyt arrhenomanes BR729 COI
Pyt aristosporum CBS26338 COI*
Pyt arrhenomanes BR1028 COI
Pyt arrhenomanes BR122 COI
Pyt arrhenomanes BR140 COI
Pyt arrhenomanes BR625 COI
Pyt arrhenomanes CBS32462 COI*
Pyt arrhenomanes Lev1728 COI
Pyt arrhenomanes BR667 COI
Pyt arrhenomanes BR712 COI
Pyt arrhenomanes BR889 COI
Pyt arrhenomanes BR975 COI
Pyt arrhenomanes BR982 COI
Pyt arrhenomanes BR817 COI
Pyt arrhenomanes BR981 COI
Pyt arrhenomanes BR983 COI
Pyt arrhenomanes BR985 COI
Pyt arrhenomanes BR980 COI
Pyt arrhenomanes BR155 COI
Pyt arrhenomanes BR619 COI
Pyt arrhenomanes BR605 COI
Pyt arrhenomanes BR606 COI
Pyt arrhenomanes Lev1538 COI
Pyt phragmitis CBS117104 COI*
Pyt sp nov BR1033 COI
Pyt contiguanum CBS22194 COI*
Pyt dissimile CBS15564 COI*
Pyt dissimile CBS52374 COI
Pyt conidiophorum BR160 COI
Pyt conidiophorum BR260 COI
Pyt conidiophorum Lev1624 COI
Pyt salpingophorum BR1024 COI
Pyt conidiophorum Lev1465 COI
Pyt salpingophorum CBS47150 COI
Pyt conidiophorum CBS22388 COI
Pyt conidiophorum CBS22488 COI
Pyt salpingophorum BR750 COI
Pyt tracheiphilum BR659 COI
Pyt tracheiphilum BR931 COI
Pyt tracheiphilum CBS32365 COI*
Pyt tracheiphilum BR932 COI
Pyt insidiosum CBS57485 COI*
Pyt insidiosum CBS57885 COI
Pyt insidiosum CBS58085 COI
Pyt insidiosum CBS57785 COI
Phy epistomium CBS59085 COI*
Hal exoprolifera CBS25293 COI
Peronospora aparines HV97 COI
Peronospora calotheca HV81 COI
Peronospora sherardiae HV211 COI
Peronospora violae HV34 COI
Peronospora radii HV22 COI
Peronospora conglomerata HV26 COI
Peronospora valerianellae HV41 COI
Phy aff citricola BR1073 COI
Phy pini CBS18125 COI*
Phy citricola P0767 COI
Phy inflata p122 COI
Phy citricola BR519 COI
Phy citricola CBS22188 COI*
Phy citricola P0716 COI
Phy citricola IIp52 COI
Phy plurivora CBS37961 COI
Phy citricola P1805 COI
Phy plurivora CBS117378 COI
Phy capsici CBS111333 COI
Phy capsici CBS12823 COI*
Phy capsici P3605 COI
Phy capsici P1319 COI
Phy capsici CBS37072 COI
Phy capsici p8 COI
Phy capsici CBS111334 COI
Phy capsici CBS111335 COI
Phy capsici CBS111336 COI
Phy capsici CBS55488 COI
Phy capsici P10386 COI
Phy mexicana P0646 COI
Phy capsici CBS25493 COI
Phy capsici P3375 COI
Phy sp "glovera" P10618 COI
Phy sp "glovera" P10619 COI
Phy tropicalis CBS43491 COI*
Phy tropicalis P6522 COI
Phy tropicalis p27 COI
Phy mengei P10139 COI
Phy mengei P1275 COI
Phy sp nov P1212 COI
Phy siskiyouensis P15122 COI
Phy siskiyouensis P15123 COI
Phy siskiyouensis P16301 COI
Phy siskiyouensis PRI816 COI
Phy siskiyouensis PRI817 COI
Phy multivora P7902 COI
Phy multivora CBS119108 COI
Phy multivora CBS113347 COI
Phy multivora BR514 COI

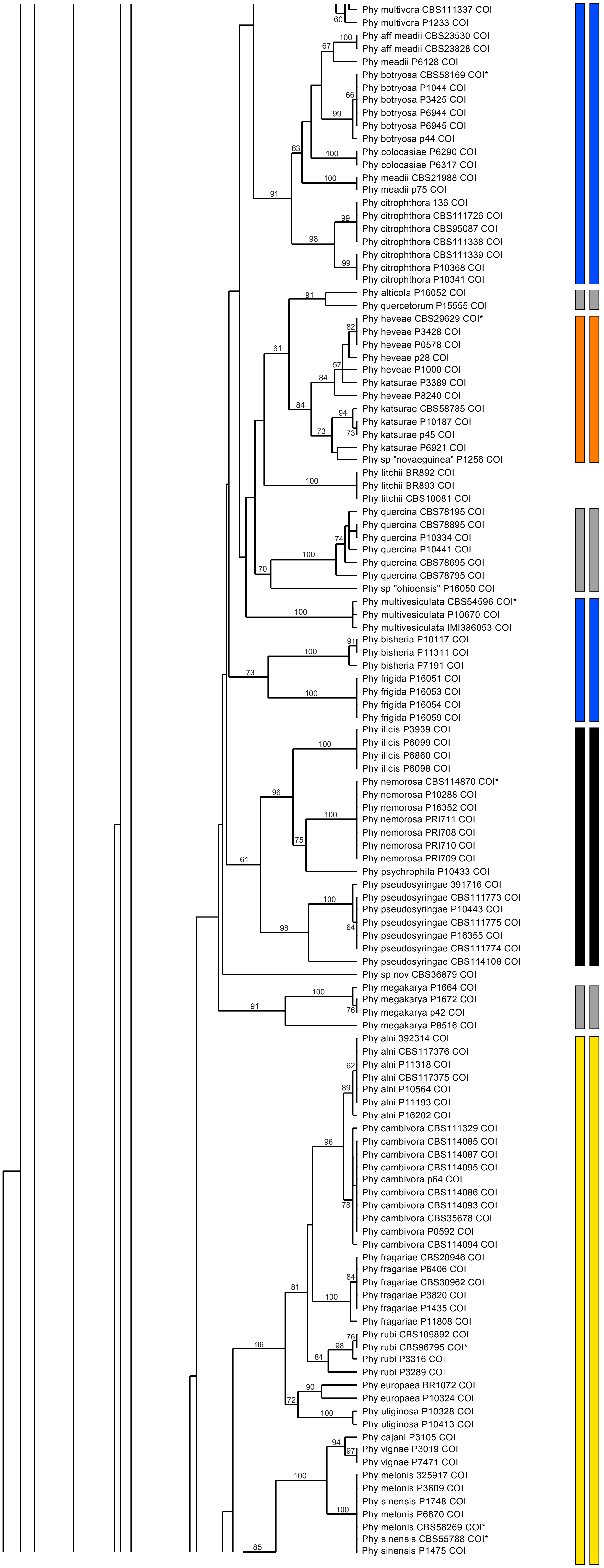

Phy multivora CBS111337 COI
Phy multivora P1233 COI
Phy aff meadii CBS23530 COI
Phy aff meadii CBS23828 COI
Phy meadii P6128 COI
Phy botryosa CBS58169 COI*
Phy botryosa P1044 COI
Phy botryosa P3425 COI
Phy botryosa P6944 COI
Phy botryosa P6945 COI
Phy botryosa p44 COI
Phy colocasiae P6290 COI
Phy colocasiae P6317 COI
Phy meadii CBS21988 COI
Phy meadii p75 COI
Phy citrophthora 136 COI
Phy citrophthora CBS111726 COI
Phy citrophthora CBS95087 COI
Phy citrophthora CBS111338 COI
Phy citrophthora CBS111339 COI
Phy citrophthora P10368 COI
Phy citrophthora P10341 COI
Phy alticola P16052 COI
Phy quercetorum P15555 COI
Phy heveae CBS29629 COI*
Phy heveae P3428 COI
Phy heveae P0578 COI
Phy heveae p28 COI
Phy heveae P1000 COI
Phy katsurae P3389 COI
Phy heveae P8240 COI
Phy katsurae CBS58785 COI
Phy katsurae P10187 COI
Phy katsurae p45 COI
Phy katsurae P6921 COI
Phy sp "novaeguinea" P1256 COI
Phy litchii BR892 COI
Phy litchii BR893 COI
Phy litchii CBS10081 COI
Phy quercina CBS78195 COI
Phy quercina CBS78895 COI
Phy quercina P10334 COI
Phy quercina P10441 COI
Phy quercina CBS78695 COI
Phy quercina CBS78795 COI
Phy sp "ohioensis" P16050 COI
Phy multivesiculata CBS54596 COI*
Phy multivesiculata P10670 COI
Phy multivesiculata IMI386053 COI
Phy bisheria P10117 COI
Phy bisheria P11311 COI
Phy bisheria P7191 COI
Phy frigida P16051 COI
Phy frigida P16053 COI
Phy frigida P16054 COI
Phy frigida P16059 COI
Phy ilicis P3939 COI
Phy ilicis P6099 COI
Phy ilicis P6860 COI
Phy ilicis P6098 COI
Phy nemorosa CBS114870 COI*
Phy nemorosa P10288 COI
Phy nemorosa P16352 COI
Phy nemorosa PRI711 COI
Phy nemorosa PRI708 COI
Phy nemorosa PRI710 COI
Phy nemorosa PRI709 COI
Phy psychrophila P10433 COI
Phy pseudosyringae 391716 COI
Phy pseudosyringae CBS111773 COI
Phy pseudosyringae P10443 COI
Phy pseudosyringae CBS111775 COI
Phy pseudosyringae P16355 COI
Phy pseudosyringae CBS111774 COI
Phy pseudosyringae CBS114108 COI
Phy sp nov CBS36879 COI
Phy megakarya P1664 COI
Phy megakarya P1672 COI
Phy megakarya p42 COI
Phy megakarya P8516 COI
Phy alni 392314 COI
Phy alni CBS117376 COI
Phy alni P11318 COI
Phy alni CBS117375 COI
Phy alni P10564 COI
Phy alni P11193 COI
Phy alni P16202 COI
Phy cambivora CBS111329 COI
Phy cambivora CBS114085 COI
Phy cambivora CBS114087 COI
Phy cambivora CBS114095 COI
Phy cambivora p64 COI
Phy cambivora CBS114086 COI
Phy cambivora CBS114093 COI
Phy cambivora CBS35678 COI
Phy cambivora P0592 COI
Phy cambivora CBS114094 COI
Phy fragariae CBS20946 COI
Phy fragariae P6406 COI
Phy fragariae CBS30962 COI
Phy fragariae P3820 COI
Phy fragariae P1435 COI
Phy fragariae P11808 COI
Phy rubi CBS109892 COI
Phy rubi CBS96795 COI*
Phy rubi P3316 COI
Phy rubi P3289 COI
Phy europaea BR1072 COI
Phy europaea P10324 COI
Phy uliginosa P10328 COI
Phy uliginosa P10413 COI
Phy cajani P3105 COI
Phy vignae P3019 COI
Phy vignae P7471 COI
Phy melonis 325917 COI
Phy melonis P3609 COI
Phy sinensis P1748 COI
Phy melonis P6870 COI
Phy melonis CBS58269 COI*
Phy sinensis CBS55788 COI*
Phy sinensis P1475 COI

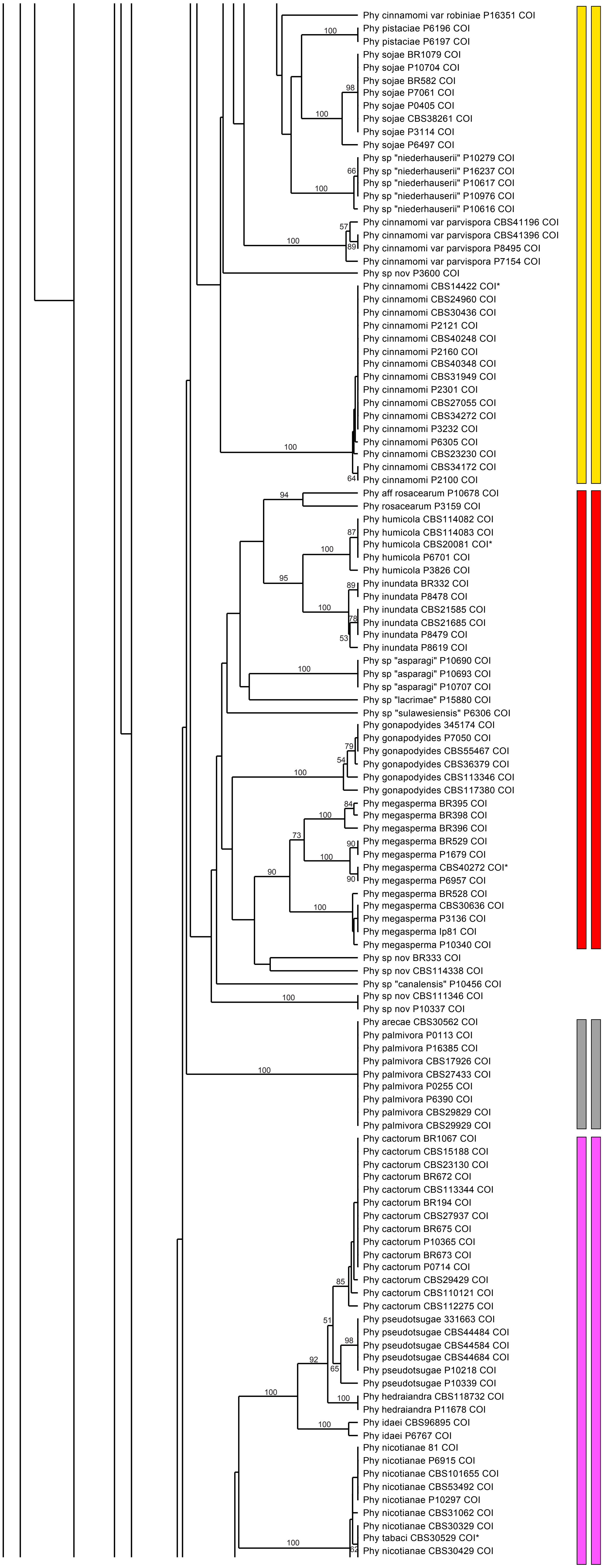
Phy cinnamomi var robiniae P16351 COI
Phy pistaciae P6196 COI
Phy pistaciae P6197 COI
Phy sojae BR1079 COI
Phy sojae P10704 COI
Phy sojae BR582 COI
Phy sojae P7061 COI
Phy sojae P0405 COI
Phy sojae CBS38261 COI
Phy sojae P3114 COI
Phy sojae P6497 COI
Phy sp "niederhauserii" P10279 COI
Phy sp "niederhauserii" P16237 COI
Phy sp "niederhauserii" P10617 COI
Phy sp "niederhauserii" P10976 COI
Phy sp "niederhauserii" P10616 COI
Phy cinnamomi var parvispora CBS41196 COI
Phy cinnamomi var parvispora CBS41396 COI
Phy cinnamomi var parvispora P8495 COI
Phy cinnamomi var parvispora P7154 COI
Phy sp nov P3600 COI
Phy cinnamomi CBS14422 COI*
Phy cinnamomi CBS24960 COI
Phy cinnamomi CBS30436 COI
Phy cinnamomi P2121 COI
Phy cinnamomi CBS40248 COI
Phy cinnamomi P2160 COI
Phy cinnamomi CBS40348 COI
Phy cinnamomi CBS31949 COI
Phy cinnamomi P2301 COI
Phy cinnamomi CBS27055 COI
Phy cinnamomi CBS34272 COI
Phy cinnamomi P3232 COI
Phy cinnamomi P6305 COI
Phy cinnamomi CBS23230 COI
Phy cinnamomi CBS34172 COI
Phy cinnamomi P2100 COI
Phy aff rosacearum P10678 COI
Phy rosacearum P3159 COI
Phy humicola CBS114082 COI
Phy humicola CBS114083 COI
Phy humicola CBS20081 COI*
Phy humicola P6701 COI
Phy humicola P3826 COI
Phy inundata BR332 COI
Phy inundata P8478 COI
Phy inundata CBS21585 COI
Phy inundata CBS21685 COI
Phy inundata P8479 COI
Phy inundata P8619 COI
Phy sp "asparagi" P10690 COI
Phy sp "asparagi" P10693 COI
Phy sp "asparagi" P10707 COI
Phy sp "lacrimae" P15880 COI
Phy sp "sulawesiensis" P6306 COI
Phy gonapodyides 345174 COI
Phy gonapodyides P7050 COI
Phy gonapodyides CBS55467 COI
Phy gonapodyides CBS36379 COI
Phy gonapodyides CBS113346 COI
Phy gonapodyides CBS117380 COI
Phy megasperma BR395 COI
Phy megasperma BR398 COI
Phy megasperma BR396 COI
Phy megasperma BR529 COI
Phy megasperma P1679 COI
Phy megasperma CBS40272 COI*
Phy megasperma P6957 COI
Phy megasperma BR528 COI
Phy megasperma CBS30636 COI
Phy megasperma P3136 COI
Phy megasperma Ip81 COI
Phy megasperma P10340 COI
Phy sp nov BR333 COI
Phy sp nov CBS114338 COI
Phy sp "canalensis" P10456 COI
Phy sp nov CBS111346 COI
Phy sp nov P10337 COI
Phy arecae CBS30562 COI
Phy palmivora P0113 COI
Phy palmivora P16385 COI
Phy palmivora CBS17926 COI
Phy palmivora CBS27433 COI
Phy palmivora P0255 COI
Phy palmivora P6390 COI
Phy palmivora CBS29829 COI
Phy palmivora CBS29929 COI
Phy cactorum BR1067 COI
Phy cactorum CBS15188 COI
Phy cactorum CBS23130 COI
Phy cactorum BR672 COI
Phy cactorum CBS113344 COI
Phy cactorum BR194 COI
Phy cactorum CBS27937 COI
Phy cactorum BR675 COI
Phy cactorum P10365 COI
Phy cactorum BR673 COI
Phy cactorum P0714 COI
Phy cactorum CBS29429 COI
Phy cactorum CBS110121 COI
Phy cactorum CBS112275 COI
Phy pseudotsugae 331663 COI
Phy pseudotsugae CBS44484 COI
Phy pseudotsugae CBS44584 COI
Phy pseudotsugae CBS44684 COI
Phy pseudotsugae P10218 COI
Phy pseudotsugae P10339 COI
Phy hedraiandra CBS118732 COI
Phy hedraiandra P11678 COI
Phy idaei CBS96895 COI
Phy idaei P6767 COI
Phy nicotianae 81 COI
Phy nicotianae P6915 COI
Phy nicotianae CBS101655 COI
Phy nicotianae CBS53492 COI
Phy nicotianae P10297 COI
Phy nicotianae CBS31062 COI
Phy nicotianae CBS30329 COI
Phy tabaci CBS30529 COI*
Phy nicotianae CBS30429 COI

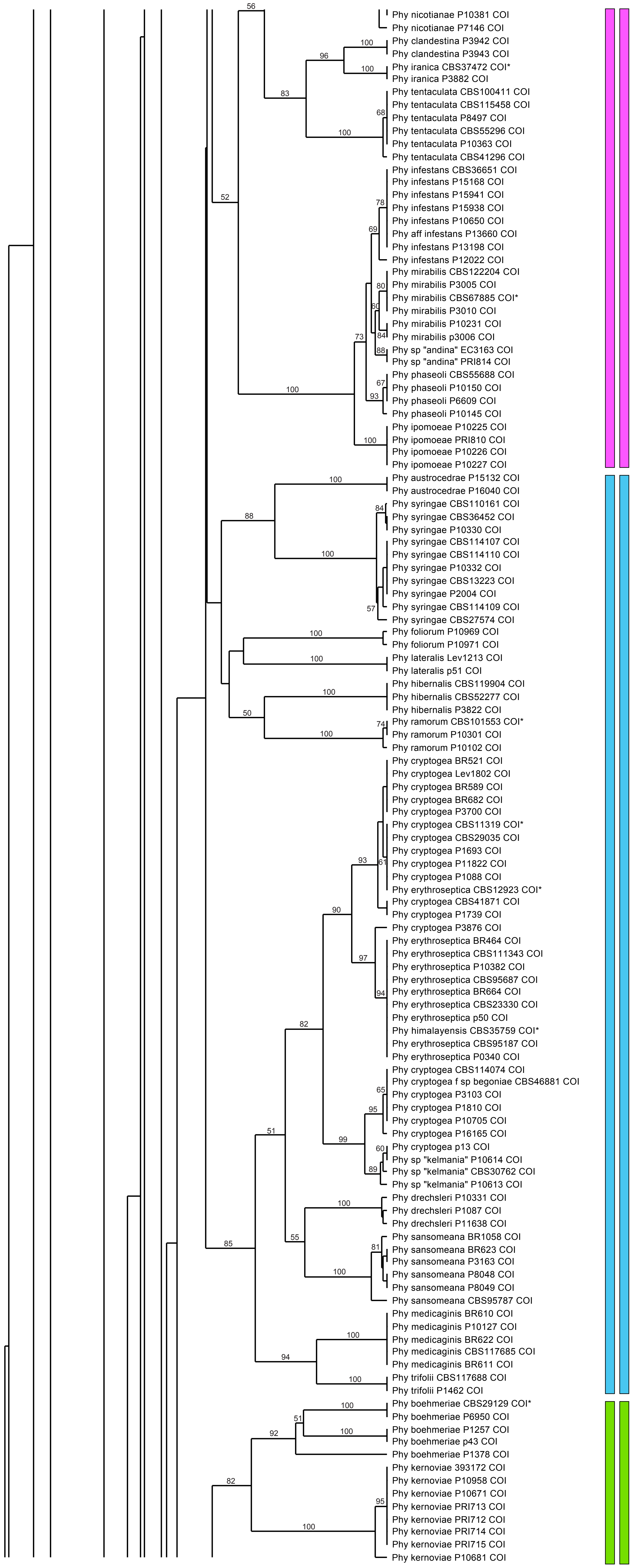
Phy nicotianae P10381 COI
Phy nicotianae P7146 COI
Phy clandestina P3942 COI
Phy clandestina P3943 COI
Phy iranica CBS37472 COI*
Phy iranica P3882 COI
Phy tentaculata CBS100411 COI
Phy tentaculata CBS115458 COI
Phy tentaculata P8497 COI
Phy tentaculata CBS55296 COI
Phy tentaculata P10363 COI
Phy tentaculata CBS41296 COI
Phy infestans CBS36651 COI
Phy infestans P15168 COI
Phy infestans P15941 COI
Phy infestans P15938 COI
Phy infestans P10650 COI
Phy aff infestans P13660 COI
Phy infestans P13198 COI
Phy infestans P12022 COI
Phy mirabilis CBS122204 COI
Phy mirabilis P3005 COI
Phy mirabilis CBS67885 COI*
Phy mirabilis P3010 COI
Phy mirabilis P10231 COI
Phy mirabilis p3006 COI
Phy sp "andina" EC3163 COI
Phy sp "andina" PRI814 COI
Phy phaseoli CBS55688 COI
Phy phaseoli P10150 COI
Phy phaseoli P6609 COI
Phy phaseoli P10145 COI
Phy ipomoeae P10225 COI
Phy ipomoeae PRI810 COI
Phy ipomoeae P10226 COI
Phy ipomoeae P10227 COI
Phy austrocedrae P15132 COI
Phy austrocedrae P16040 COI
Phy syringae CBS110161 COI
Phy syringae CBS36452 COI
Phy syringae P10330 COI
Phy syringae CBS114107 COI
Phy syringae CBS114110 COI
Phy syringae P10332 COI
Phy syringae CBS13223 COI
Phy syringae P2004 COI
Phy syringae CBS114109 COI
Phy syringae CBS27574 COI
Phy foliorum P10969 COI
Phy foliorum P10971 COI
Phy lateralis Lev1213 COI
Phy lateralis p51 COI
Phy hibernalis CBS119904 COI
Phy hibernalis CBS52277 COI
Phy hibernalis P3822 COI
Phy ramorum CBS101553 COI*
Phy ramorum P10301 COI
Phy ramorum P10102 COI
Phy cryptogea BR521 COI
Phy cryptogea Lev1802 COI
Phy cryptogea BR589 COI
Phy cryptogea BR682 COI
Phy cryptogea P3700 COI
Phy cryptogea CBS11319 COI*
Phy cryptogea CBS29035 COI
Phy cryptogea P1693 COI
Phy cryptogea P11822 COI
Phy cryptogea P1088 COI
Phy erythroseptica CBS12923 COI*
Phy cryptogea CBS41871 COI
Phy cryptogea P1739 COI
Phy cryptogea P3876 COI
Phy erythroseptica BR464 COI
Phy erythroseptica CBS111343 COI
Phy erythroseptica P10382 COI
Phy erythroseptica CBS95687 COI
Phy erythroseptica BR664 COI
Phy erythroseptica CBS23330 COI
Phy erythroseptica p50 COI
Phy himalayensis CBS35759 COI*
Phy erythroseptica CBS95187 COI
Phy erythroseptica P0340 COI
Phy cryptogea CBS114074 COI
Phy cryptogea f sp begoniae CBS46881 COI
Phy cryptogea P3103 COI
Phy cryptogea P1810 COI
Phy cryptogea P10705 COI
Phy cryptogea P16165 COI
Phy cryptogea p13 COI
Phy sp "kelmania" P10614 COI
Phy sp "kelmania" CBS30762 COI
Phy sp "kelmania" P10613 COI
Phy drechsleri P10331 COI
Phy drechsleri P1087 COI
Phy drechsleri P11638 COI
Phy sansomeana BR1058 COI
Phy sansomeana BR623 COI
Phy sansomeana P3163 COI
Phy sansomeana P8048 COI
Phy sansomeana P8049 COI
Phy sansomeana CBS95787 COI
Phy medicaginis BR610 COI
Phy medicaginis P10127 COI
Phy medicaginis BR622 COI
Phy medicaginis CBS117685 COI
Phy medicaginis BR611 COI
Phy trifolii CBS117688 COI
Phy trifolii P1462 COI
Phy boehmeriae CBS29129 COI*
Phy boehmeriae P6950 COI
Phy boehmeriae P1257 COI
Phy boehmeriae p43 COI
Phy boehmeriae P1378 COI
Phy kernoviae 393172 COI
Phy kernoviae P10958 COI
Phy kernoviae P10671 COI
Phy kernoviae PRI713 COI
Phy kernoviae PRI712 COI
Phy kernoviae PRI714 COI
Phy kernoviae PRI715 COI
Phy kernoviae P10681 COI

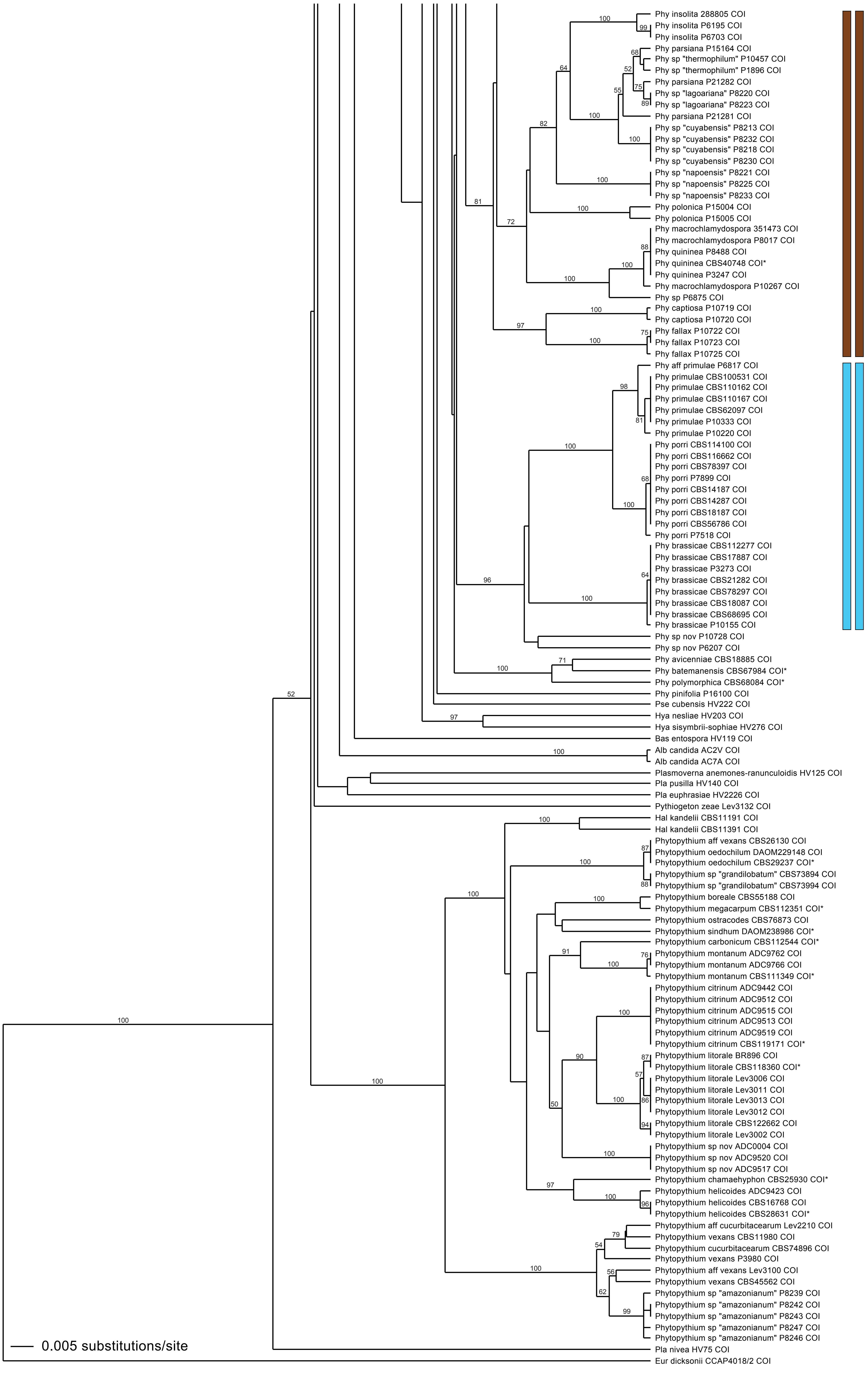

Phy insolita 288805 COI
Phy insolita P6195 COI
Phy insolita P6703 COI
Phy parsiana P15164 COI
Phy sp "thermophilum" P10457 COI
Phy sp "thermophilum" P1896 COI
Phy parsiana P21282 COI
Phy sp "lagoariana" P8220 COI
Phy sp "lagoariana" P8223 COI
Phy parsiana P21281 COI
Phy sp "cuyabensis" P8213 COI
Phy sp "cuyabensis" P8232 COI
Phy sp "cuyabensis" P8218 COI
Phy sp "cuyabensis" P8230 COI
Phy sp "napoensis" P8221 COI
Phy sp "napoensis" P8225 COI
Phy sp "napoensis" P8233 COI
Phy polonica P15004 COI
Phy polonica P15005 COI
Phy macrochlamydospora 351473 COI
Phy macrochlamydospora P8017 COI
Phy quininea P8488 COI
Phy quininea CBS40748 COI*
Phy quininea P3247 COI
Phy macrochlamydospora P10267 COI
Phy sp P6875 COI
Phy captiosa P10719 COI
Phy captiosa P10720 COI
Phy fallax P10722 COI
Phy fallax P10723 COI
Phy fallax P10725 COI
Phy aff primulae P6817 COI
Phy primulae CBS100531 COI
Phy primulae CBS110162 COI
Phy primulae CBS110167 COI
Phy primulae CBS62097 COI
Phy primulae P10333 COI
Phy primulae P10220 COI
Phy porri CBS114100 COI
Phy porri CBS116662 COI
Phy porri CBS78397 COI
Phy porri P7899 COI
Phy porri CBS14187 COI
Phy porri CBS14287 COI
Phy porri CBS18187 COI
Phy porri CBS56786 COI
Phy porri P7518 COI
Phy brassicae CBS112277 COI
Phy brassicae CBS17887 COI
Phy brassicae P3273 COI
Phy brassicae CBS21282 COI
Phy brassicae CBS78297 COI
Phy brassicae CBS18087 COI
Phy brassicae CBS68695 COI
Phy brassicae P10155 COI
Phy sp nov P10728 COI
Phy sp nov P6207 COI
Phy avicenniae CBS18885 COI
Phy batemanensis CBS67984 COI*
Phy polymorphica CBS68084 COI*
Phy pinifolia P16100 COI
Pse cubensis HV222 COI
Hya nesliae HV203 COI
Hya sisymbrii-sophiae HV276 COI
Bas entospora HV119 COI
Alb candida AC2V COI
Alb candida AC7A COI
Plasmoverna anemones-ranunculoidis HV125 COI
Pla pusilla HV140 COI
Pla euphrasiae HV2226 COI
Pythiogeton zeae Lev3132 COI
Hal kandelii CBS11191 COI
Hal kandelii CBS11391 COI
Phytopythium aff vexans CBS26130 COI
Phytopythium oedochilum DAOM229148 COI
Phytopythium oedochilum CBS29237 COI*
Phytopythium sp "grandilobatum" CBS73894 COI
Phytopythium sp "grandilobatum" CBS73994 COI
Phytopythium boreale CBS55188 COI
Phytopythium megacarpum CBS112351 COI*
Phytopythium ostracodes CBS76873 COI
Phytopythium sindhum DAOM238986 COI*
Phytopythium carbonicum CBS112544 COI*
Phytopythium montanum ADC9762 COI
Phytopythium montanum ADC9766 COI
Phytopythium montanum CBS111349 COI*
Phytopythium citrinum ADC9442 COI
Phytopythium citrinum ADC9512 COI
Phytopythium citrinum ADC9515 COI
Phytopythium citrinum ADC9513 COI
Phytopythium citrinum ADC9519 COI
Phytopythium citrinum CBS119171 COI*
Phytopythium litorale BR896 COI
Phytopythium litorale CBS118360 COI*
Phytopythium litorale Lev3006 COI
Phytopythium litorale Lev3011 COI
Phytopythium litorale Lev3013 COI
Phytopythium litorale Lev3012 COI
Phytopythium litorale CBS122662 COI
Phytopythium litorale Lev3002 COI
Phytopythium sp nov ADC0004 COI
Phytopythium sp nov ADC9520 COI
Phytopythium sp nov ADC9517 COI
Phytopythium chamaehyphon CBS25930 COI*
Phytopythium helicoides ADC9423 COI
Phytopythium helicoides CBS16768 COI
Phytopythium helicoides CBS28631 COI*
Phytopythium aff cucurbitacearum Lev2210 COI
Phytopythium vexans CBS11980 COI
Phytopythium cucurbitacearum CBS74896 COI
Phytopythium vexans P3980 COI
Phytopythium aff vexans Lev3100 COI
Phytopythium vexans CBS45562 COI
Phytopythium sp "amazonianum" P8239 COI
Phytopythium sp "amazonianum" P8242 COI
Phytopythium sp "amazonianum" P8243 COI
Phytopythium sp "amazonianum" P8247 COI
Phytopythium sp "amazonianum" P8246 COI
Pla nivea HV75 COI
Eur dicksonii CCAP4018/2 COI
0.005 substitutions/site

* denotes ex-type specimen

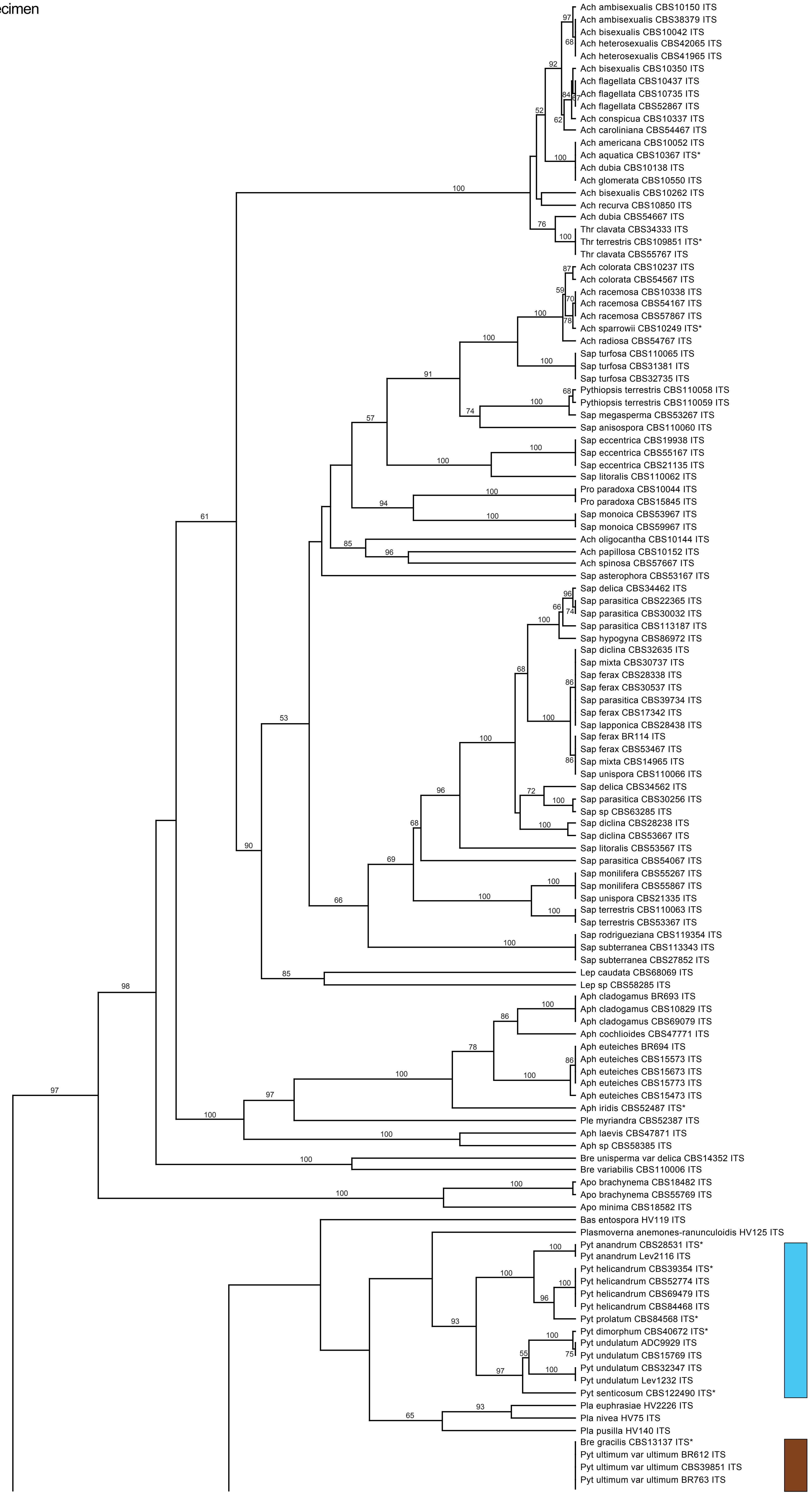

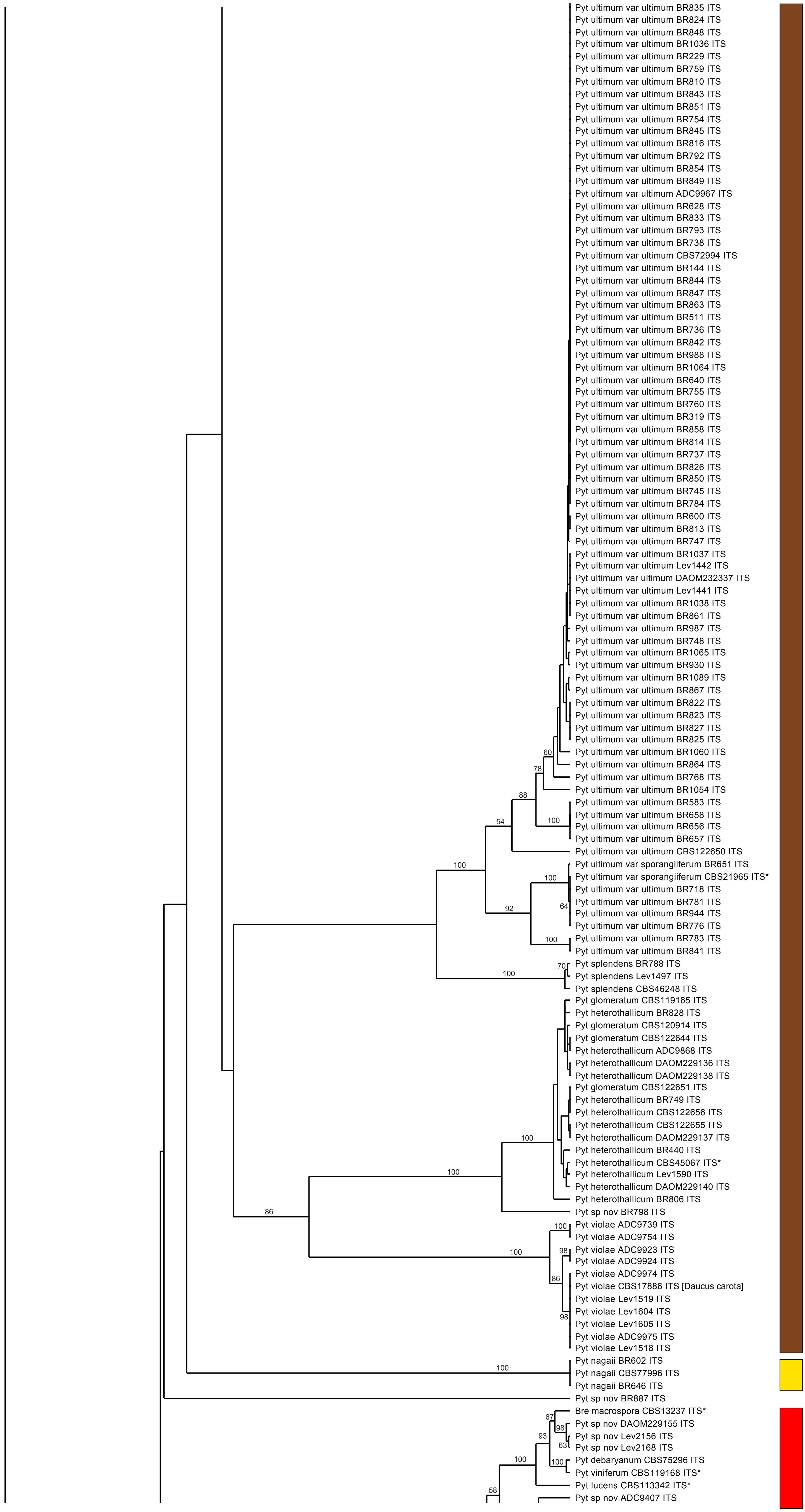

Pyt ultimum var ultimum BR835 ITS
Pyt ultimum var ultimum BR824 ITS
Pyt ultimum var ultimum BR848 ITS
Pyt ultimum var ultimum BR1036 ITS
Pyt ultimum var ultimum BR229 ITS
Pyt ultimum var ultimum BR759 ITS
Pyt ultimum var ultimum BR810 ITS
Pyt ultimum var ultimum BR843 ITS
Pyt ultimum var ultimum BR851 ITS
Pyt ultimum var ultimum BR754 ITS
Pyt ultimum var ultimum BR845 ITS
Pyt ultimum var ultimum BR816 ITS
Pyt ultimum var ultimum BR792 ITS
Pyt ultimum var ultimum BR854 ITS
Pyt ultimum var ultimum BR849 ITS
Pyt ultimum var ultimum ADC9967 ITS
Pyt ultimum var ultimum BR628 ITS
Pyt ultimum var ultimum BR833 ITS
Pyt ultimum var ultimum BR793 ITS
Pyt ultimum var ultimum BR738 ITS
Pyt ultimum var ultimum CBS72994 ITS
Pyt ultimum var ultimum BR144 ITS
Pyt ultimum var ultimum BR844 ITS
Pyt ultimum var ultimum BR847 ITS
Pyt ultimum var ultimum BR863 ITS
Pyt ultimum var ultimum BR511 ITS
Pyt ultimum var ultimum BR736 ITS
Pyt ultimum var ultimum BR842 ITS
Pyt ultimum var ultimum BR988 ITS
Pyt ultimum var ultimum BR1064 ITS
Pyt ultimum var ultimum BR640 ITS
Pyt ultimum var ultimum BR755 ITS
Pyt ultimum var ultimum BR760 ITS
Pyt ultimum var ultimum BR319 ITS
Pyt ultimum var ultimum BR858 ITS
Pyt ultimum var ultimum BR814 ITS
Pyt ultimum var ultimum BR737 ITS
Pyt ultimum var ultimum BR826 ITS
Pyt ultimum var ultimum BR850 ITS
Pyt ultimum var ultimum BR745 ITS
Pyt ultimum var ultimum BR784 ITS
Pyt ultimum var ultimum BR600 ITS
Pyt ultimum var ultimum BR813 ITS
Pyt ultimum var ultimum BR747 ITS
Pyt ultimum var ultimum BR1037 ITS
Pyt ultimum var ultimum Lev1442 ITS
Pyt ultimum var ultimum DAOM232337 ITS
Pyt ultimum var ultimum Lev1441 ITS
Pyt ultimum var ultimum BR1038 ITS
Pyt ultimum var ultimum BR861 ITS
Pyt ultimum var ultimum BR987 ITS
Pyt ultimum var ultimum BR748 ITS
Pyt ultimum var ultimum BR1065 ITS
Pyt ultimum var ultimum BR930 ITS
Pyt ultimum var ultimum BR1089 ITS
Pyt ultimum var ultimum BR867 ITS
Pyt ultimum var ultimum BR822 ITS
Pyt ultimum var ultimum BR823 ITS
Pyt ultimum var ultimum BR827 ITS
Pyt ultimum var ultimum BR825 ITS
Pyt ultimum var ultimum BR1060 ITS
Pyt ultimum var ultimum BR864 ITS
Pyt ultimum var ultimum BR768 ITS
Pyt ultimum var ultimum BR1054 ITS
Pyt ultimum var ultimum BR583 ITS
Pyt ultimum var ultimum BR658 ITS
Pyt ultimum var ultimum BR656 ITS
Pyt ultimum var ultimum BR657 ITS
Pyt ultimum var ultimum CBS122650 ITS
Pyt ultimum var sporangiiferum BR651 ITS
Pyt ultimum var sporangiiferum CBS21965 ITS*
Pyt ultimum var ultimum BR718 ITS
Pyt ultimum var ultimum BR781 ITS
Pyt ultimum var ultimum BR944 ITS
Pyt ultimum var ultimum BR776 ITS
Pyt ultimum var ultimum BR783 ITS
Pyt ultimum var ultimum BR841 ITS
Pyt splendens BR788 ITS
Pyt splendens Lev1497 ITS
Pyt splendens CBS46248 ITS
Pyt glomeratum CBS119165 ITS
Pyt heterothallicum BR828 ITS
Pyt glomeratum CBS120914 ITS
Pyt glomeratum CBS122644 ITS
Pyt heterothallicum ADC9868 ITS
Pyt heterothallicum DAOM229136 ITS
Pyt heterothallicum DAOM229138 ITS
Pyt glomeratum CBS122651 ITS
Pyt heterothallicum BR749 ITS
Pyt heterothallicum CBS122656 ITS
Pyt heterothallicum CBS122655 ITS
Pyt heterothallicum DAOM229137 ITS
Pyt heterothallicum BR440 ITS
Pyt heterothallicum CBS45067 ITS*
Pyt heterothallicum Lev1590 ITS
Pyt heterothallicum DAOM229140 ITS
Pyt heterothallicum BR806 ITS
Pyt sp nov BR798 ITS
Pyt violae ADC9739 ITS
Pyt violae ADC9754 ITS
Pyt violae ADC9923 ITS
Pyt violae ADC9924 ITS
Pyt violae ADC9974 ITS
Pyt violae CBS17886 ITS [Daucus carota]
Pyt violae Lev1519 ITS
Pyt violae Lev1604 ITS
Pyt violae Lev1605 ITS
Pyt violae ADC9975 ITS
Pyt violae Lev1518 ITS
Pyt nagaii BR602 ITS
Pyt nagaii CBS77996 ITS
Pyt nagaii BR646 ITS
Pyt sp nov BR887 ITS
Bre macrospora CBS13237 ITS*
Pyt sp nov DAOM229155 ITS
Pyt sp nov Lev2156 ITS
Pyt sp nov Lev2168 ITS
Pyt debaryanum CBS75296 ITS
Pyt viniferum CBS119168 ITS*
Pyt lucens CBS113342 ITS*
Pyt sp nov ADC9407 ITS

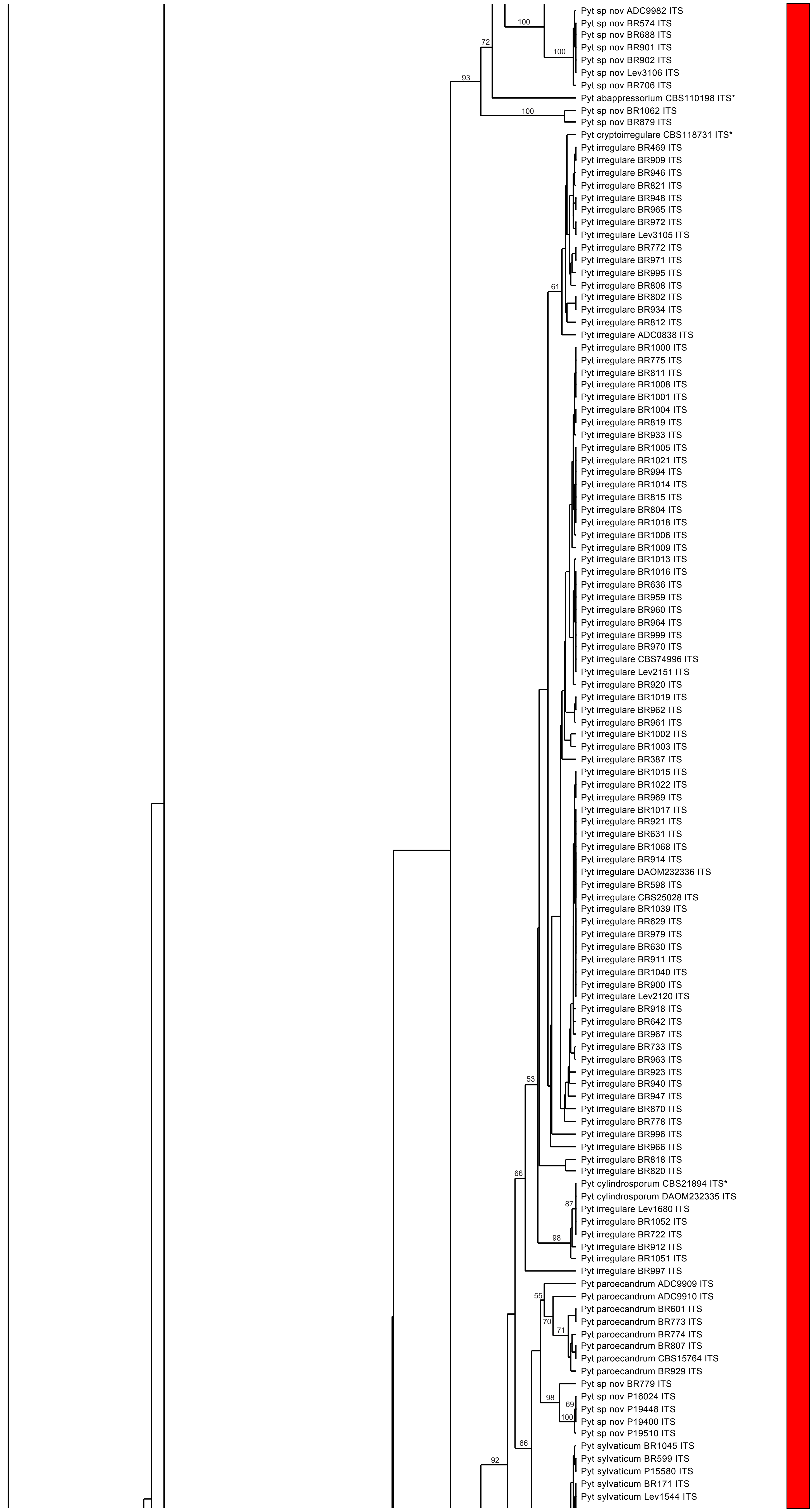

Pyt sp nov ADC9982 ITS
Pyt sp nov BR574 ITS
Pyt sp nov BR688 ITS
Pyt sp nov BR901 ITS
Pyt sp nov BR902 ITS
Pyt sp nov Lev3106 ITS
Pyt sp nov BR706 ITS
Pyt abappressorium CBS110198 ITS*
Pyt sp nov BR1062 ITS
Pyt sp nov BR879 ITS
Pyt cryptoirregulare CBS118731 ITS*
Pyt irregulare BR469 ITS
Pyt irregulare BR909 ITS
Pyt irregulare BR946 ITS
Pyt irregulare BR821 ITS
Pyt irregulare BR948 ITS
Pyt irregulare BR965 ITS
Pyt irregulare BR972 ITS
Pyt irregulare Lev3105 ITS
Pyt irregulare BR772 ITS
Pyt irregulare BR971 ITS
Pyt irregulare BR995 ITS
Pyt irregulare BR808 ITS
Pyt irregulare BR802 ITS
Pyt irregulare BR934 ITS
Pyt irregulare BR812 ITS
Pyt irregulare ADC0838 ITS
Pyt irregulare BR1000 ITS
Pyt irregulare BR775 ITS
Pyt irregulare BR811 ITS
Pyt irregulare BR1008 ITS
Pyt irregulare BR1001 ITS
Pyt irregulare BR1004 ITS
Pyt irregulare BR819 ITS
Pyt irregulare BR933 ITS
Pyt irregulare BR1005 ITS
Pyt irregulare BR1021 ITS
Pyt irregulare BR994 ITS
Pyt irregulare BR1014 ITS
Pyt irregulare BR815 ITS
Pyt irregulare BR804 ITS
Pyt irregulare BR1018 ITS
Pyt irregulare BR1006 ITS
Pyt irregulare BR1009 ITS
Pyt irregulare BR1013 ITS
Pyt irregulare BR1016 ITS
Pyt irregulare BR636 ITS
Pyt irregulare BR959 ITS
Pyt irregulare BR960 ITS
Pyt irregulare BR964 ITS
Pyt irregulare BR999 ITS
Pyt irregulare BR970 ITS
Pyt irregulare CBS74996 ITS
Pyt irregulare Lev2151 ITS
Pyt irregulare BR920 ITS
Pyt irregulare BR1019 ITS
Pyt irregulare BR962 ITS
Pyt irregulare BR961 ITS
Pyt irregulare BR1002 ITS
Pyt irregulare BR1003 ITS
Pyt irregulare BR387 ITS
Pyt irregulare BR1015 ITS
Pyt irregulare BR1022 ITS
Pyt irregulare BR969 ITS
Pyt irregulare BR1017 ITS
Pyt irregulare BR921 ITS
Pyt irregulare BR631 ITS
Pyt irregulare BR1068 ITS
Pyt irregulare BR914 ITS
Pyt irregulare DAOM232336 ITS
Pyt irregulare BR598 ITS
Pyt irregulare CBS25028 ITS
Pyt irregulare BR1039 ITS
Pyt irregulare BR629 ITS
Pyt irregulare BR979 ITS
Pyt irregulare BR630 ITS
Pyt irregulare BR911 ITS
Pyt irregulare BR1040 ITS
Pyt irregulare BR900 ITS
Pyt irregulare Lev2120 ITS
Pyt irregulare BR918 ITS
Pyt irregulare BR642 ITS
Pyt irregulare BR967 ITS
Pyt irregulare BR733 ITS
Pyt irregulare BR963 ITS
Pyt irregulare BR923 ITS
Pyt irregulare BR940 ITS
Pyt irregulare BR947 ITS
Pyt irregulare BR870 ITS
Pyt irregulare BR778 ITS
Pyt irregulare BR996 ITS
Pyt irregulare BR966 ITS
Pyt irregulare BR818 ITS
Pyt irregulare BR820 ITS
Pyt cylindrosporum CBS21894 ITS*
Pyt cylindrosporum DAOM232335 ITS
Pyt irregulare Lev1680 ITS
Pyt irregulare BR1052 ITS
Pyt irregulare BR722 ITS
Pyt irregulare BR912 ITS
Pyt irregulare BR1051 ITS
Pyt irregulare BR997 ITS
Pyt paroecandrum ADC9909 ITS
Pyt paroecandrum ADC9910 ITS
Pyt paroecandrum BR601 ITS
Pyt paroecandrum BR773 ITS
Pyt paroecandrum BR774 ITS
Pyt paroecandrum BR807 ITS
Pyt paroecandrum CBS15764 ITS
Pyt paroecandrum BR929 ITS
Pyt sp nov BR779 ITS
Pyt sp nov P16024 ITS
Pyt sp nov P19448 ITS
Pyt sp nov P19400 ITS
Pyt sp nov P19510 ITS
Pyt sylvaticum BR1045 ITS
Pyt sylvaticum BR599 ITS
Pyt sylvaticum P15580 ITS
Pyt sylvaticum BR171 ITS
Pyt sylvaticum Lev1544 ITS

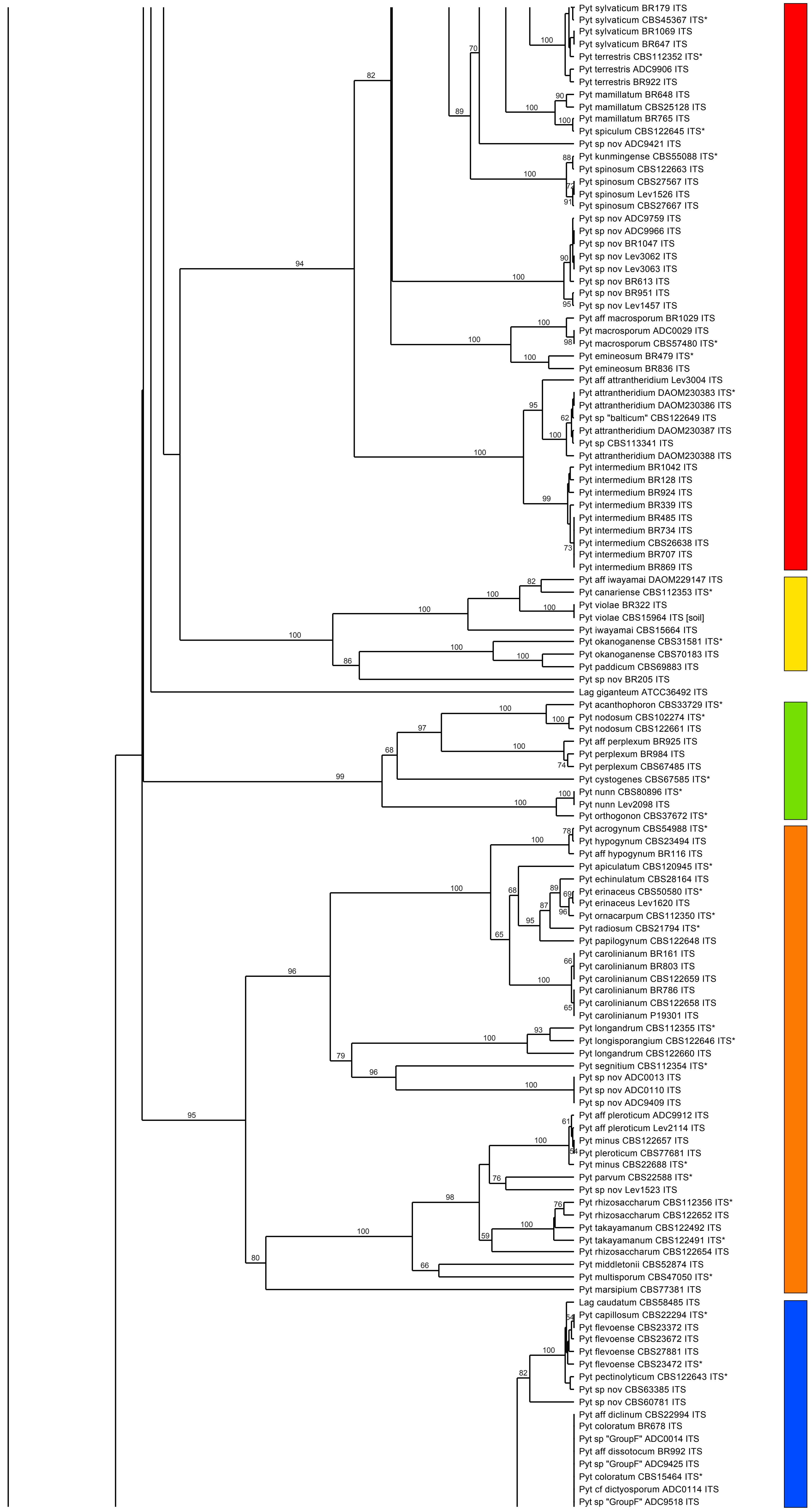
Pyt sylvaticum BR179 ITS
Pyt sylvaticum CBS45367 ITS*
Pyt sylvaticum BR1069 ITS
Pyt sylvaticum BR647 ITS
Pyt terrestris CBS112352 ITS*
Pyt terrestris ADC9906 ITS
Pyt terrestris BR922 ITS
Pyt mamillatum BR648 ITS
Pyt mamillatum CBS25128 ITS
Pyt mamillatum BR765 ITS
Pyt spiculum CBS122645 ITS*
Pyt sp nov ADC9421 ITS
Pyt kunmingense CBS55088 ITS*
Pyt spinosum CBS122663 ITS
Pyt spinosum CBS27567 ITS
Pyt spinosum Lev1526 ITS
Pyt spinosum CBS27667 ITS
Pyt sp nov ADC9759 ITS
Pyt sp nov ADC9966 ITS
Pyt sp nov BR1047 ITS
Pyt sp nov Lev3062 ITS
Pyt sp nov Lev3063 ITS
Pyt sp nov BR613 ITS
Pyt sp nov BR951 ITS
Pyt sp nov Lev1457 ITS
Pyt aff macrosporum BR1029 ITS
Pyt macrosporum ADC0029 ITS
Pyt macrosporum CBS57480 ITS*
Pyt emineosum BR479 ITS*
Pyt emineosum BR836 ITS
Pyt aff attrantheridium Lev3004 ITS
Pyt attrantheridium DAOM230383 ITS*
Pyt attrantheridium DAOM230386 ITS
Pyt sp "balticum" CBS122649 ITS
Pyt attrantheridium DAOM230387 ITS
Pyt sp CBS113341 ITS
Pyt attrantheridium DAOM230388 ITS
Pyt intermedium BR1042 ITS
Pyt intermedium BR128 ITS
Pyt intermedium BR924 ITS
Pyt intermedium BR339 ITS
Pyt intermedium BR485 ITS
Pyt intermedium BR734 ITS
Pyt intermedium CBS26638 ITS
Pyt intermedium BR707 ITS
Pyt intermedium BR869 ITS
Pyt aff iwayamai DAOM229147 ITS
Pyt canariense CBS112353 ITS*
Pyt violae BR322 ITS
Pyt violae CBS15964 ITS [soil]
Pyt iwayamai CBS15664 ITS
Pyt okanoganense CBS31581 ITS*
Pyt okanoganense CBS70183 ITS
Pyt paddicum CBS69883 ITS
Pyt sp nov BR205 ITS
Lag giganteum ATCC36492 ITS
Pyt acanthophoron CBS33729 ITS*
Pyt nodosum CBS102274 ITS*
Pyt nodosum CBS122661 ITS
Pyt aff perplexum BR925 ITS
Pyt perplexum BR984 ITS
Pyt perplexum CBS67485 ITS
Pyt cystogenes CBS67585 ITS*
Pyt nunn CBS80896 ITS*
Pyt nunn Lev2098 ITS
Pyt orthogonon CBS37672 ITS*
Pyt acrogynum CBS54988 ITS*
Pyt hypogynum CBS23494 ITS
Pyt aff hypogynum BR116 ITS
Pyt apiculatum CBS120945 ITS*
Pyt echinulatum CBS28164 ITS
Pyt erinaceus CBS50580 ITS*
Pyt erinaceus Lev1620 ITS
Pyt ornacarpum CBS112350 ITS*
Pyt radiosum CBS21794 ITS*
Pyt papilogynum CBS122648 ITS
Pyt carolinianum BR161 ITS
Pyt carolinianum BR803 ITS
Pyt carolinianum CBS122659 ITS
Pyt carolinianum BR786 ITS
Pyt carolinianum CBS122658 ITS
Pyt carolinianum P19301 ITS
Pyt longandrum CBS112355 ITS*
Pyt longisporangium CBS122646 ITS*
Pyt longandrum CBS122660 ITS
Pyt segnitium CBS112354 ITS*
Pyt sp nov ADC0013 ITS
Pyt sp nov ADC0110 ITS
Pyt sp nov ADC9409 ITS
Pyt aff pleroticum ADC9912 ITS
Pyt aff pleroticum Lev2114 ITS
Pyt minus CBS122657 ITS
Pyt pleroticum CBS77681 ITS
Pyt minus CBS22688 ITS*
Pyt parvum CBS22588 ITS*
Pyt sp nov Lev1523 ITS
Pyt rhizosaccharum CBS112356 ITS*
Pyt rhizosaccharum CBS122652 ITS
Pyt takayamanum CBS122492 ITS
Pyt takayamanum CBS122491 ITS*
Pyt rhizosaccharum CBS122654 ITS
Pyt middletonii CBS52874 ITS
Pyt multisporum CBS47050 ITS*
Pyt marsipium CBS77381 ITS
Lag caudatum CBS58485 ITS
Pyt capillosum CBS22294 ITS*
Pyt flevoense CBS23372 ITS
Pyt flevoense CBS23672 ITS
Pyt flevoense CBS27881 ITS
Pyt flevoense CBS23472 ITS*
Pyt pectinolyticum CBS122643 ITS*
Pyt sp nov CBS63385 ITS
Pyt sp nov CBS60781 ITS
Pyt aff diclinum CBS22994 ITS
Pyt coloratum BR678 ITS
Pyt sp "GroupF" ADC0014 ITS
Pyt aff dissotocum BR992 ITS
Pyt sp "GroupF" ADC9425 ITS
Pyt coloratum CBS15464 ITS*
Pyt cf dictyosporum ADC0114 ITS
Pyt sp "GroupF" ADC9518 ITS

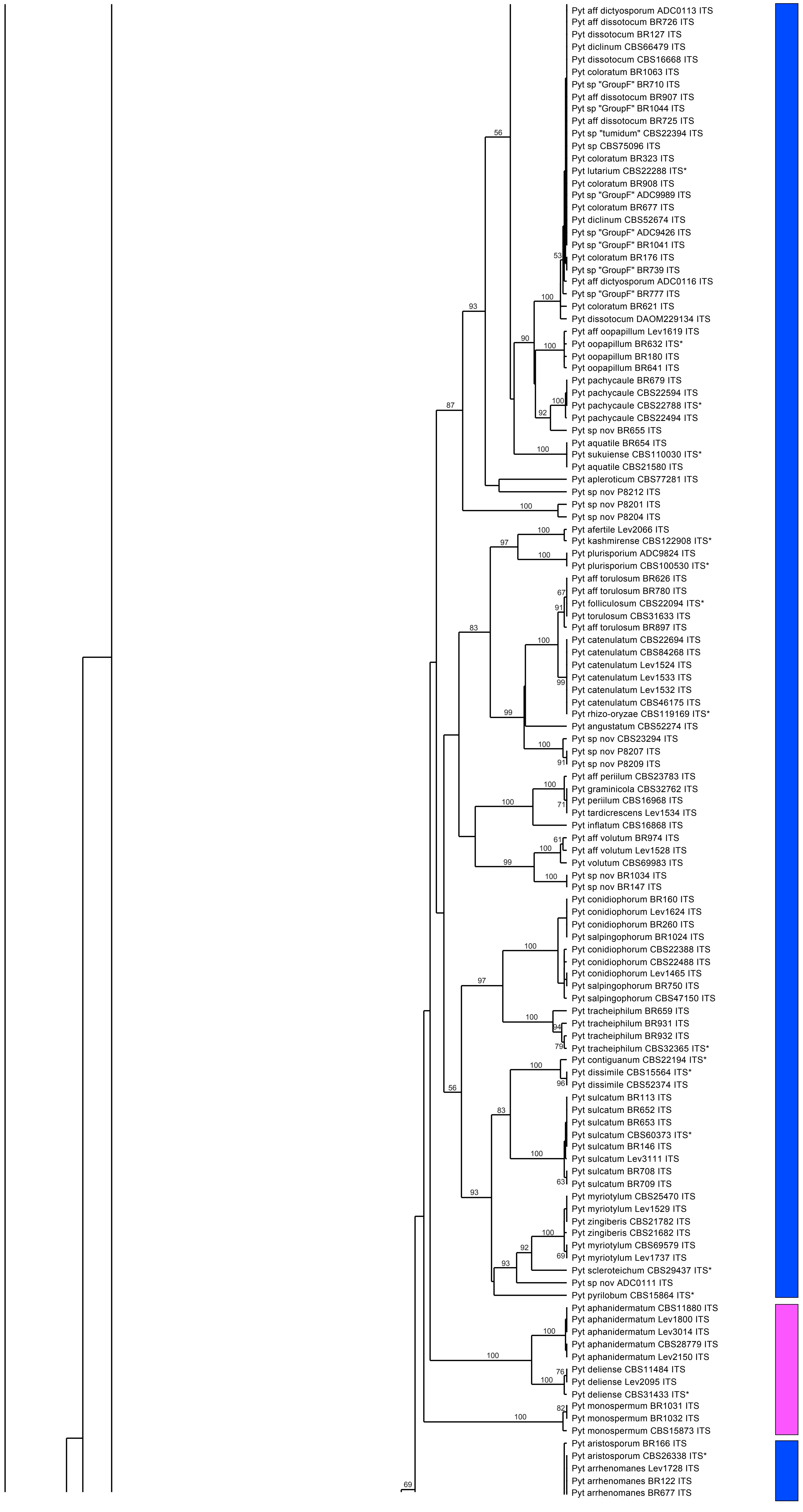
Pyt aff dictyosporum ADC0113 ITS
Pyt aff dissotocum BR726 ITS
Pyt dissotocum BR127 ITS
Pyt diclinum CBS66479 ITS
Pyt dissotocum CBS16668 ITS
Pyt coloratum BR1063 ITS
Pyt sp "GroupF" BR710 ITS
Pyt aff dissotocum BR907 ITS
Pyt sp "GroupF" BR1044 ITS
Pyt aff dissotocum BR725 ITS
Pyt sp "tumidum" CBS22394 ITS
Pyt sp CBS75096 ITS
Pyt coloratum BR323 ITS
Pyt lutarium CBS22288 ITS*
Pyt coloratum BR908 ITS
Pyt sp "GroupF" ADC9989 ITS
Pyt coloratum BR677 ITS
Pyt diclinum CBS52674 ITS
Pyt sp "GroupF" ADC9426 ITS
Pyt sp "GroupF" BR1041 ITS
Pyt coloratum BR176 ITS
Pyt sp "GroupF" BR739 ITS
Pyt aff dictyosporum ADC0116 ITS
Pyt sp "GroupF" BR777 ITS
Pyt coloratum BR621 ITS
Pyt dissotocum DAOM229134 ITS
Pyt aff oopapillum Lev1619 ITS
Pyt oopapillum BR632 ITS*
Pyt oopapillum BR180 ITS
Pyt oopapillum BR641 ITS
Pyt pachycaule BR679 ITS
Pyt pachycaule CBS22594 ITS
Pyt pachycaule CBS22788 ITS*
Pyt pachycaule CBS22494 ITS
Pyt sp nov BR655 ITS
Pyt aquatile BR654 ITS
Pyt sukuiense CBS110030 ITS*
Pyt aquatile CBS21580 ITS
Pyt apleroticum CBS77281 ITS
Pyt sp nov P8212 ITS
Pyt sp nov P8201 ITS
Pyt sp nov P8204 ITS
Pyt afertile Lev2066 ITS
Pyt kashmirense CBS122908 ITS*
Pyt plurisporium ADC9824 ITS
Pyt plurisporium CBS100530 ITS*
Pyt aff torulosum BR626 ITS
Pyt aff torulosum BR780 ITS
Pyt folliculosum CBS22094 ITS*
Pyt torulosum CBS31633 ITS
Pyt aff torulosum BR897 ITS
Pyt catenulatum CBS22694 ITS
Pyt catenulatum CBS84268 ITS
Pyt catenulatum Lev1524 ITS
Pyt catenulatum Lev1533 ITS
Pyt catenulatum Lev1532 ITS
Pyt catenulatum CBS46175 ITS
Pyt rhizo-oryzae CBS119169 ITS*
Pyt angustatum CBS52274 ITS
Pyt sp nov CBS23294 ITS
Pyt sp nov P8207 ITS
Pyt sp nov P8209 ITS
Pyt aff periilum CBS23783 ITS
Pyt graminicola CBS32762 ITS
Pyt periilum CBS16968 ITS
Pyt tardicrescens Lev1534 ITS
Pyt inflatum CBS16868 ITS
Pyt aff volutum BR974 ITS
Pyt aff volutum Lev1528 ITS
Pyt volutum CBS69983 ITS
Pyt sp nov BR1034 ITS
Pyt sp nov BR147 ITS
Pyt conidiophorum BR160 ITS
Pyt conidiophorum Lev1624 ITS
Pyt conidiophorum BR260 ITS
Pyt salpingophorum BR1024 ITS
Pyt conidiophorum CBS22388 ITS
Pyt conidiophorum CBS22488 ITS
Pyt conidiophorum Lev1465 ITS
Pyt salpingophorum BR750 ITS
Pyt salpingophorum CBS47150 ITS
Pyt tracheiphilum BR659 ITS
Pyt tracheiphilum BR931 ITS
Pyt tracheiphilum BR932 ITS
Pyt tracheiphilum CBS32365 ITS*
Pyt contiguanum CBS22194 ITS*
Pyt dissimile CBS15564 ITS*
Pyt dissimile CBS52374 ITS
Pyt sulcatum BR113 ITS
Pyt sulcatum BR652 ITS
Pyt sulcatum BR653 ITS
Pyt sulcatum CBS60373 ITS*
Pyt sulcatum BR146 ITS
Pyt sulcatum Lev3111 ITS
Pyt sulcatum BR708 ITS
Pyt sulcatum BR709 ITS
Pyt myriotylum CBS25470 ITS
Pyt myriotylum Lev1529 ITS
Pyt zingiberis CBS21782 ITS
Pyt zingiberis CBS21682 ITS
Pyt myriotylum CBS69579 ITS
Pyt myriotylum Lev1737 ITS
Pyt scleroteichum CBS29437 ITS*
Pyt sp nov ADC0111 ITS
Pyt pyrilobum CBS15864 ITS*
Pyt aphanidermatum CBS11880 ITS
Pyt aphanidermatum Lev1800 ITS
Pyt aphanidermatum Lev3014 ITS
Pyt aphanidermatum CBS28779 ITS
Pyt aphanidermatum Lev2150 ITS
Pyt deliense CBS11484 ITS
Pyt deliense Lev2095 ITS
Pyt deliense CBS31433 ITS*
Pyt monospermum BR1031 ITS
Pyt monospermum BR1032 ITS
Pyt monospermum CBS15873 ITS
Pyt aristosporum BR166 ITS
Pyt aristosporum CBS26338 ITS*
Pyt arrhenomanes Lev1728 ITS
Pyt arrhenomanes BR122 ITS
Pyt arrhenomanes BR677 ITS

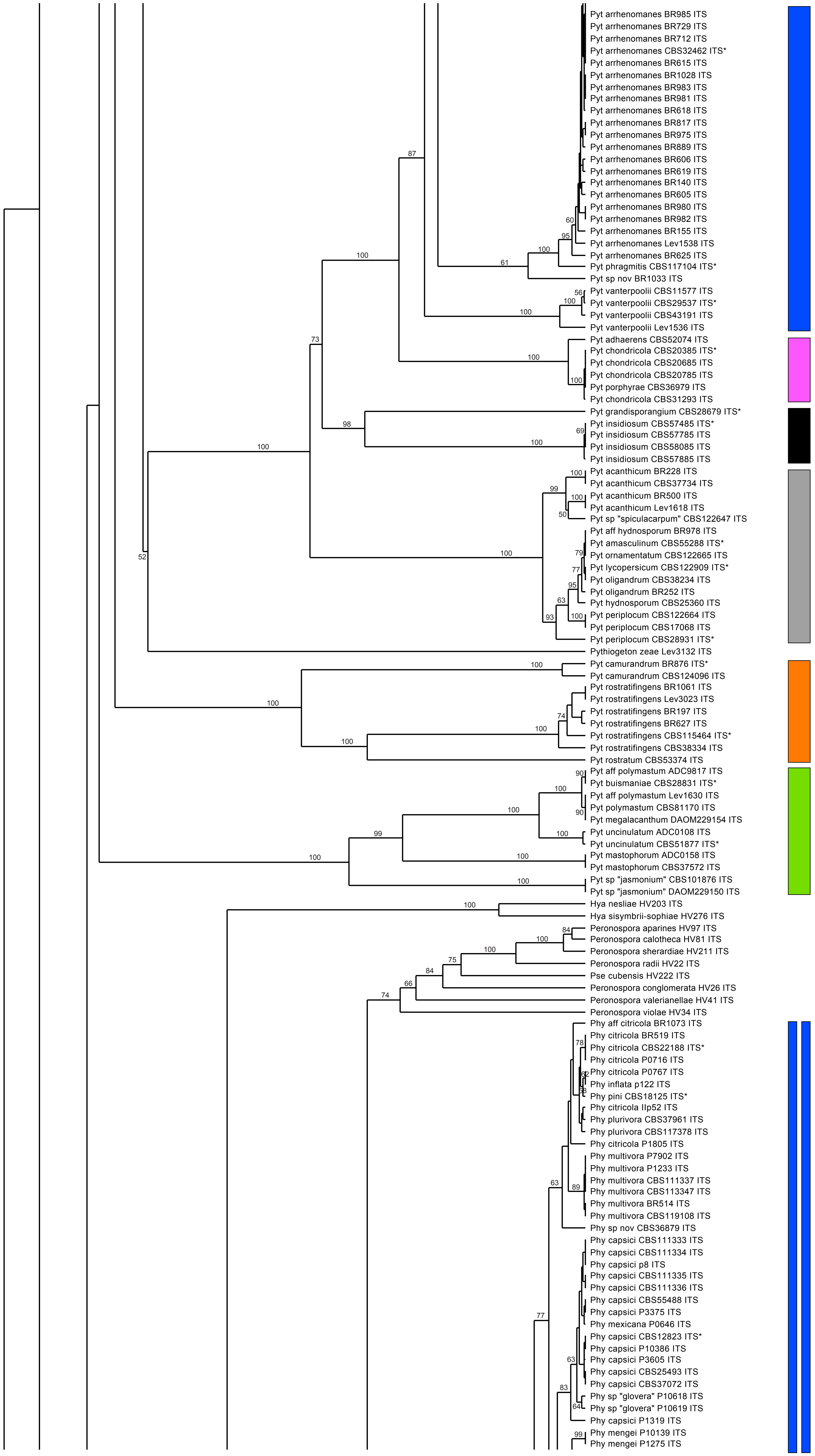
Pyt arrhenomanes BR985 ITS
Pyt arrhenomanes BR729 ITS
Pyt arrhenomanes BR712 ITS
Pyt arrhenomanes CBS32462 ITS*
Pyt arrhenomanes BR615 ITS
Pyt arrhenomanes BR1028 ITS
Pyt arrhenomanes BR983 ITS
Pyt arrhenomanes BR981 ITS
Pyt arrhenomanes BR618 ITS
Pyt arrhenomanes BR817 ITS
Pyt arrhenomanes BR975 ITS
Pyt arrhenomanes BR889 ITS
Pyt arrhenomanes BR606 ITS
Pyt arrhenomanes BR619 ITS
Pyt arrhenomanes BR140 ITS
Pyt arrhenomanes BR605 ITS
Pyt arrhenomanes BR980 ITS
Pyt arrhenomanes BR982 ITS
Pyt arrhenomanes BR155 ITS
Pyt arrhenomanes Lev1538 ITS
Pyt arrhenomanes BR625 ITS
Pyt phragmitis CBS117104 ITS*
Pyt sp nov BR1033 ITS
Pyt vanterpoolii CBS11577 ITS
Pyt vanterpoolii CBS29537 ITS*
Pyt vanterpoolii CBS43191 ITS
Pyt vanterpoolii Lev1536 ITS
Pyt adhaerens CBS52074 ITS
Pyt chondricola CBS20385 ITS*
Pyt chondricola CBS20685 ITS
Pyt chondricola CBS20785 ITS
Pyt porphyrae CBS36979 ITS
Pyt chondricola CBS31293 ITS
Pyt grandisporangium CBS28679 ITS*
Pyt insidiosum CBS57485 ITS*
Pyt insidiosum CBS57785 ITS
Pyt insidiosum CBS58085 ITS
Pyt insidiosum CBS57885 ITS
Pyt acanthicum BR228 ITS
Pyt acanthicum CBS37734 ITS
Pyt acanthicum BR500 ITS
Pyt acanthicum Lev1618 ITS
Pyt sp "spiculacarpum" CBS122647 ITS
Pyt aff hydnosporum BR978 ITS
Pyt amasculinum CBS55288 ITS*
Pyt ornamentatum CBS122665 ITS
Pyt lycopersicum CBS122909 ITS*
Pyt oligandrum CBS38234 ITS
Pyt oligandrum BR252 ITS
Pyt hydnosporum CBS25360 ITS
Pyt periplocum CBS122664 ITS
Pyt periplocum CBS17068 ITS
Pyt periplocum CBS28931 ITS*
Pythiogeton zeae Lev3132 ITS
Pyt camurandrum BR876 ITS*
Pyt camurandrum CBS124096 ITS
Pyt rostratifingens BR1061 ITS
Pyt rostratifingens Lev3023 ITS
Pyt rostratifingens BR197 ITS
Pyt rostratifingens BR627 ITS
Pyt rostratifingens CBS115464 ITS*
Pyt rostratifingens CBS38334 ITS
Pyt rostratum CBS53374 ITS
Pyt aff polymastum ADC9817 ITS
Pyt buismaniae CBS28831 ITS*
Pyt aff polymastum Lev1630 ITS
Pyt polymastum CBS81170 ITS
Pyt megalacanthum DAOM229154 ITS
Pyt uncinulatum ADC0108 ITS
Pyt uncinulatum CBS51877 ITS*
Pyt mastophorum ADC0158 ITS
Pyt mastophorum CBS37572 ITS
Pyt sp "jasmonium" CBS101876 ITS
Pyt sp "jasmonium" DAOM229150 ITS
Hya nesliae HV203 ITS
Hya sisymbrii-sophiae HV276 ITS
Peronospora aparines HV97 ITS
Peronospora calotheca HV81 ITS
Peronospora sherardiae HV211 ITS
Peronospora radii HV22 ITS
Pse cubensis HV222 ITS
Peronospora conglomerata HV26 ITS
Peronospora valerianellae HV41 ITS
Peronospora violae HV34 ITS
Phy aff citricola BR1073 ITS
Phy citricola BR519 ITS
Phy citricola CBS22188 ITS*
Phy citricola P0716 ITS
Phy citricola P0767 ITS
Phy inflata p122 ITS
Phy pini CBS18125 ITS*
Phy citricola IIp52 ITS
Phy plurivora CBS37961 ITS
Phy plurivora CBS117378 ITS
Phy citricola P1805 ITS
Phy multivora P7902 ITS
Phy multivora P1233 ITS
Phy multivora CBS111337 ITS
Phy multivora CBS113347 ITS
Phy multivora BR514 ITS
Phy multivora CBS119108 ITS
Phy sp nov CBS36879 ITS
Phy capsici CBS111333 ITS
Phy capsici CBS111334 ITS
Phy capsici p8 ITS
Phy capsici CBS111335 ITS
Phy capsici CBS111336 ITS
Phy capsici CBS55488 ITS
Phy capsici P3375 ITS
Phy mexicana P0646 ITS
Phy capsici CBS12823 ITS*
Phy capsici P10386 ITS
Phy capsici P3605 ITS
Phy capsici CBS25493 ITS
Phy capsici CBS37072 ITS
Phy sp "glovera" P10618 ITS
Phy sp "glovera" P10619 ITS
Phy capsici P1319 ITS
Phy mengei P10139 ITS
Phy mengei P1275 ITS

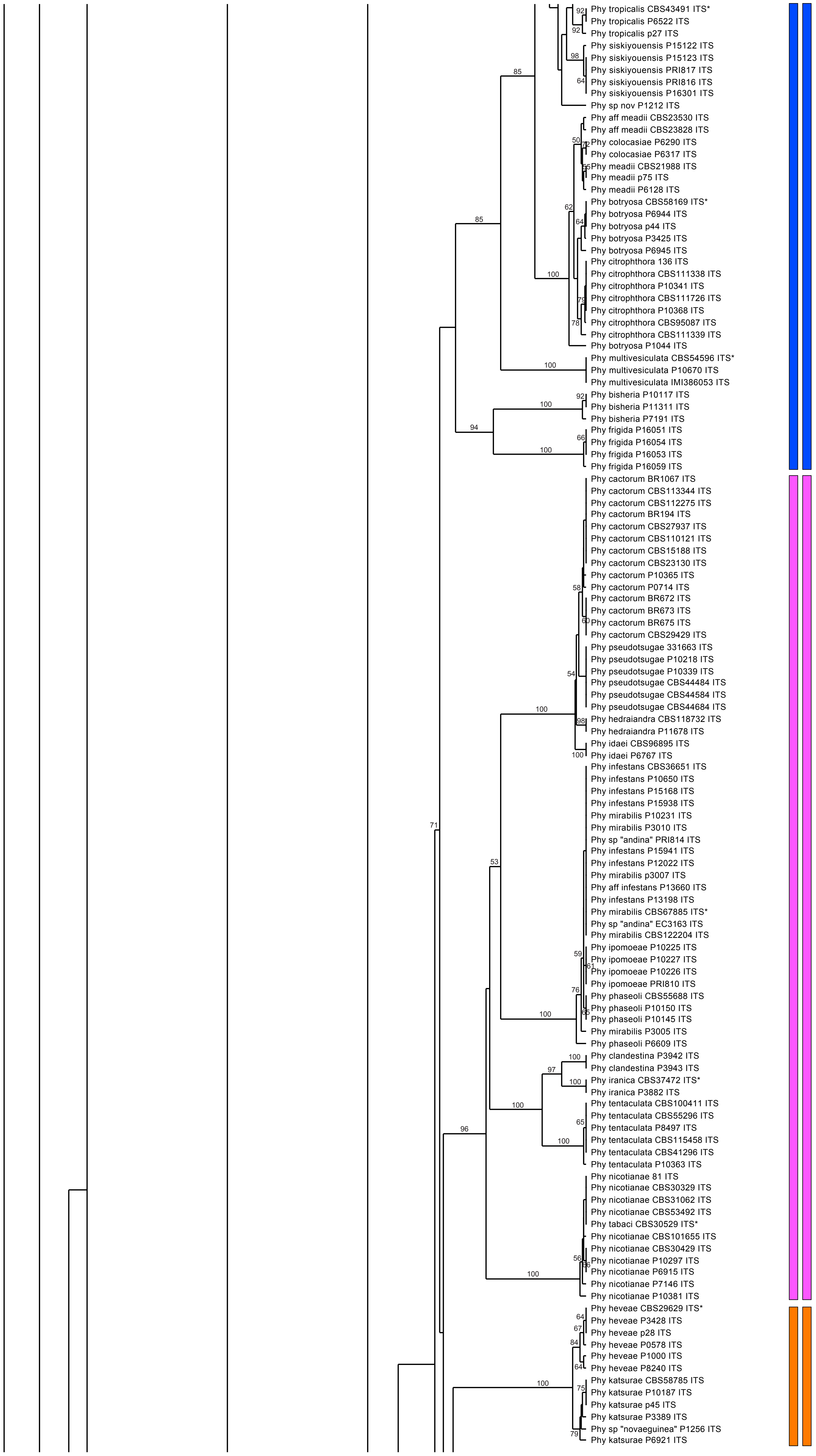
Phy tropicalis CBS43491 ITS*
Phy tropicalis P6522 ITS
Phy tropicalis p27 ITS
Phy siskiyouensis P15122 ITS
Phy siskiyouensis P15123 ITS
Phy siskiyouensis PRI817 ITS
Phy siskiyouensis PRI816 ITS
Phy siskiyouensis P16301 ITS
Phy sp nov P1212 ITS
Phy aff meadii CBS23530 ITS
Phy aff meadii CBS23828 ITS
Phy colocasiae P6290 ITS
Phy colocasiae P6317 ITS
Phy meadii CBS21988 ITS
Phy meadii p75 ITS
Phy meadii P6128 ITS
Phy botryosa CBS58169 ITS*
Phy botryosa P6944 ITS
Phy botryosa p44 ITS
Phy botryosa P3425 ITS
Phy botryosa P6945 ITS
Phy citrophthora 136 ITS
Phy citrophthora CBS111338 ITS
Phy citrophthora P10341 ITS
Phy citrophthora CBS111726 ITS
Phy citrophthora P10368 ITS
Phy citrophthora CBS95087 ITS
Phy citrophthora CBS111339 ITS
Phy botryosa P1044 ITS
Phy multivesiculata CBS54596 ITS*
Phy multivesiculata P10670 ITS
Phy multivesiculata IMI386053 ITS
Phy bisheria P10117 ITS
Phy bisheria P11311 ITS
Phy bisheria P7191 ITS
Phy frigida P16051 ITS
Phy frigida P16054 ITS
Phy frigida P16053 ITS
Phy frigida P16059 ITS
Phy cactorum BR1067 ITS
Phy cactorum CBS113344 ITS
Phy cactorum CBS112275 ITS
Phy cactorum BR194 ITS
Phy cactorum CBS27937 ITS
Phy cactorum CBS110121 ITS
Phy cactorum CBS15188 ITS
Phy cactorum CBS23130 ITS
Phy cactorum P10365 ITS
Phy cactorum P0714 ITS
Phy cactorum BR672 ITS
Phy cactorum BR673 ITS
Phy cactorum BR675 ITS
Phy cactorum CBS29429 ITS
Phy pseudotsugae 331663 ITS
Phy pseudotsugae P10218 ITS
Phy pseudotsugae P10339 ITS
Phy pseudotsugae CBS44484 ITS
Phy pseudotsugae CBS44584 ITS
Phy pseudotsugae CBS44684 ITS
Phy hedraiandra CBS118732 ITS
Phy hedraiandra P11678 ITS
Phy idaei CBS96895 ITS
Phy idaei P6767 ITS
Phy infestans CBS36651 ITS
Phy infestans P10650 ITS
Phy infestans P15168 ITS
Phy infestans P15938 ITS
Phy mirabilis P10231 ITS
Phy mirabilis P3010 ITS
Phy sp "andina" PRI814 ITS
Phy infestans P15941 ITS
Phy infestans P12022 ITS
Phy mirabilis p3007 ITS
Phy aff infestans P13660 ITS
Phy infestans P13198 ITS
Phy mirabilis CBS67885 ITS*
Phy sp "andina" EC3163 ITS
Phy mirabilis CBS122204 ITS
Phy ipomoeae P10225 ITS
Phy ipomoeae P10227 ITS
Phy ipomoeae P10226 ITS
Phy ipomoeae PRI810 ITS
Phy phaseoli CBS55688 ITS
Phy phaseoli P10150 ITS
Phy phaseoli P10145 ITS
Phy mirabilis P3005 ITS
Phy phaseoli P6609 ITS
Phy clandestina P3942 ITS
Phy clandestina P3943 ITS
Phy iranica CBS37472 ITS*
Phy iranica P3882 ITS
Phy tentaculata CBS100411 ITS
Phy tentaculata CBS55296 ITS
Phy tentaculata P8497 ITS
Phy tentaculata CBS115458 ITS
Phy tentaculata CBS41296 ITS
Phy tentaculata P10363 ITS
Phy nicotianae 81 ITS
Phy nicotianae CBS30329 ITS
Phy nicotianae CBS31062 ITS
Phy nicotianae CBS53492 ITS
Phy tabaci CBS30529 ITS*
Phy nicotianae CBS101655 ITS
Phy nicotianae CBS30429 ITS
Phy nicotianae P10297 ITS
Phy nicotianae P6915 ITS
Phy nicotianae P7146 ITS
Phy nicotianae P10381 ITS
Phy heveae CBS29629 ITS*
Phy heveae P3428 ITS
Phy heveae p28 ITS
Phy heveae P0578 ITS
Phy heveae P1000 ITS
Phy heveae P8240 ITS
Phy katsurae CBS58785 ITS
Phy katsurae P10187 ITS
Phy katsurae p45 ITS
Phy katsurae P3389 ITS
Phy sp "novaeguinea" P1256 ITS
Phy katsurae P6921 ITS

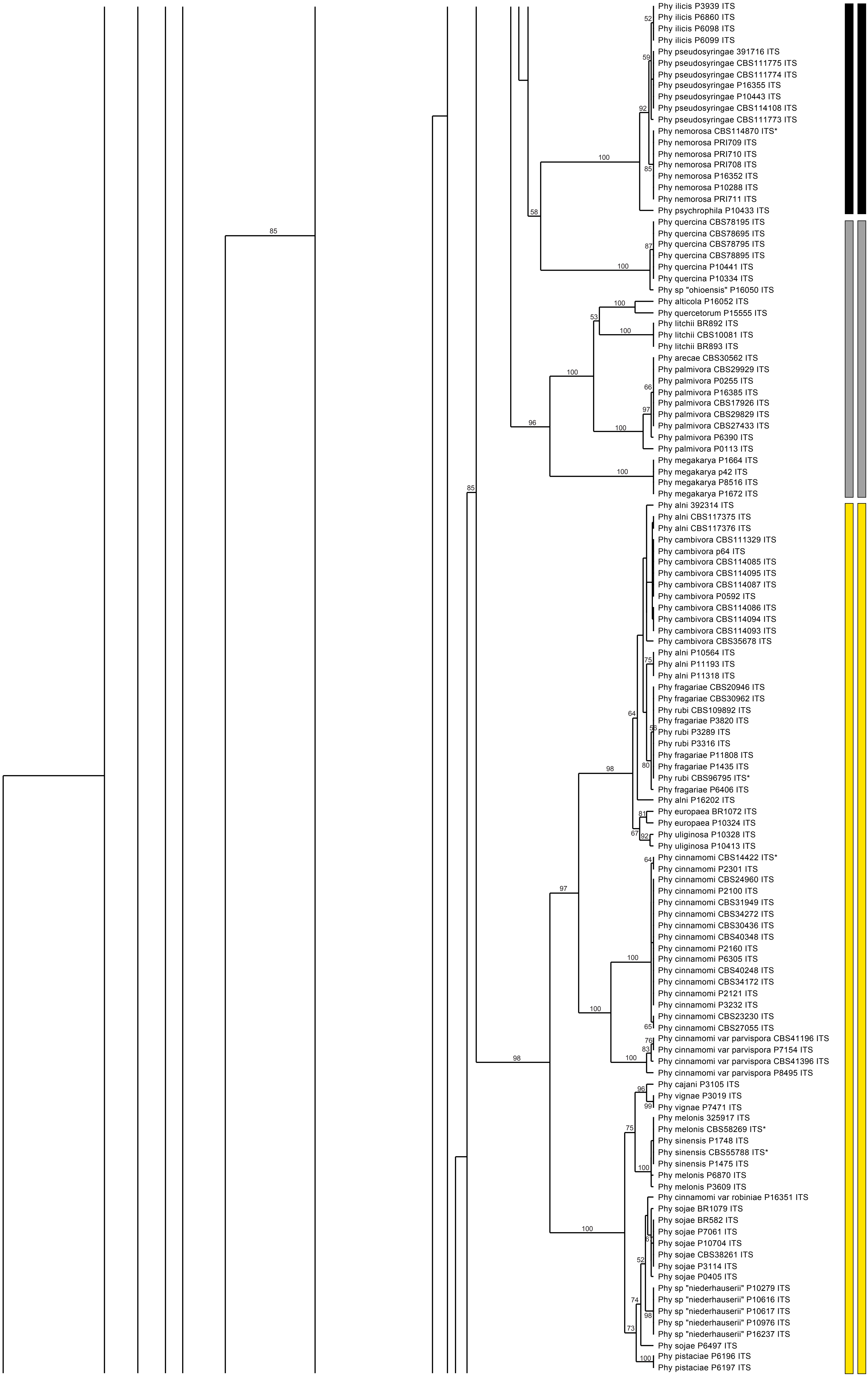

Phy ilicis P3939 ITS
Phy ilicis P6860 ITS
Phy ilicis P6098 ITS
Phy ilicis P6099 ITS
Phy pseudosyringae 391716 ITS
Phy pseudosyringae CBS111775 ITS
Phy pseudosyringae CBS111774 ITS
Phy pseudosyringae P16355 ITS
Phy pseudosyringae P10443 ITS
Phy pseudosyringae CBS114108 ITS
Phy pseudosyringae CBS111773 ITS
Phy nemorosa CBS114870 ITS*
Phy nemorosa PRI709 ITS
Phy nemorosa PRI710 ITS
Phy nemorosa PRI708 ITS
Phy nemorosa P16352 ITS
Phy nemorosa P10288 ITS
Phy nemorosa PRI711 ITS
Phy psychrophila P10433 ITS
Phy quercina CBS78195 ITS
Phy quercina CBS78695 ITS
Phy quercina CBS78795 ITS
Phy quercina CBS78895 ITS
Phy quercina P10441 ITS
Phy quercina P10334 ITS
Phy sp "ohioensis" P16050 ITS
Phy alticola P16052 ITS
Phy quercetorum P15555 ITS
Phy litchii BR892 ITS
Phy litchii CBS10081 ITS
Phy litchii BR893 ITS
Phy arecae CBS30562 ITS
Phy palmivora CBS29929 ITS
Phy palmivora P0255 ITS
Phy palmivora P16385 ITS
Phy palmivora CBS17926 ITS
Phy palmivora CBS29829 ITS
Phy palmivora CBS27433 ITS
Phy palmivora P6390 ITS
Phy palmivora P0113 ITS
Phy megakarya P1664 ITS
Phy megakarya p42 ITS
Phy megakarya P8516 ITS
Phy megakarya P1672 ITS
Phy alni 392314 ITS
Phy alni CBS117375 ITS
Phy alni CBS117376 ITS
Phy cambivora CBS111329 ITS
Phy cambivora p64 ITS
Phy cambivora CBS114085 ITS
Phy cambivora CBS114095 ITS
Phy cambivora CBS114087 ITS
Phy cambivora P0592 ITS
Phy cambivora CBS114086 ITS
Phy cambivora CBS114094 ITS
Phy cambivora CBS114093 ITS
Phy cambivora CBS35678 ITS
Phy alni P10564 ITS
Phy alni P11193 ITS
Phy alni P11318 ITS
Phy fragariae CBS20946 ITS
Phy fragariae CBS30962 ITS
Phy rubi CBS109892 ITS
Phy fragariae P3820 ITS
Phy rubi P3289 ITS
Phy rubi P3316 ITS
Phy fragariae P11808 ITS
Phy fragariae P1435 ITS
Phy rubi CBS96795 ITS*
Phy fragariae P6406 ITS
Phy alni P16202 ITS
Phy europaea BR1072 ITS
Phy europaea P10324 ITS
Phy uliginosa P10328 ITS
Phy uliginosa P10413 ITS
Phy cinnamomi CBS14422 ITS*
Phy cinnamomi P2301 ITS
Phy cinnamomi CBS24960 ITS
Phy cinnamomi P2100 ITS
Phy cinnamomi CBS31949 ITS
Phy cinnamomi CBS34272 ITS
Phy cinnamomi CBS30436 ITS
Phy cinnamomi CBS40348 ITS
Phy cinnamomi P2160 ITS
Phy cinnamomi P6305 ITS
Phy cinnamomi CBS40248 ITS
Phy cinnamomi CBS34172 ITS
Phy cinnamomi P2121 ITS
Phy cinnamomi P3232 ITS
Phy cinnamomi CBS23230 ITS
Phy cinnamomi CBS27055 ITS
Phy cinnamomi var parvispora CBS41196 ITS
Phy cinnamomi var parvispora P7154 ITS
Phy cinnamomi var parvispora CBS41396 ITS
Phy cinnamomi var parvispora P8495 ITS
Phy cajani P3105 ITS
Phy vignae P3019 ITS
Phy vignae P7471 ITS
Phy melonis 325917 ITS
Phy melonis CBS58269 ITS*
Phy sinensis P1748 ITS
Phy sinensis CBS55788 ITS*
Phy sinensis P1475 ITS
Phy melonis P6870 ITS
Phy melonis P3609 ITS
Phy cinnamomi var robiniae P16351 ITS
Phy sojae BR1079 ITS
Phy sojae BR582 ITS
Phy sojae P7061 ITS
Phy sojae P10704 ITS
Phy sojae CBS38261 ITS
Phy sojae P3114 ITS
Phy sojae P0405 ITS
Phy sp "niederhauserii" P10279 ITS
Phy sp "niederhauserii" P10616 ITS
Phy sp "niederhauserii" P10617 ITS
Phy sp "niederhauserii" P10976 ITS
Phy sp "niederhauserii" P16237 ITS
Phy sojae P6497 ITS
Phy pistaciae P6196 ITS
Phy pistaciae P6197 ITS

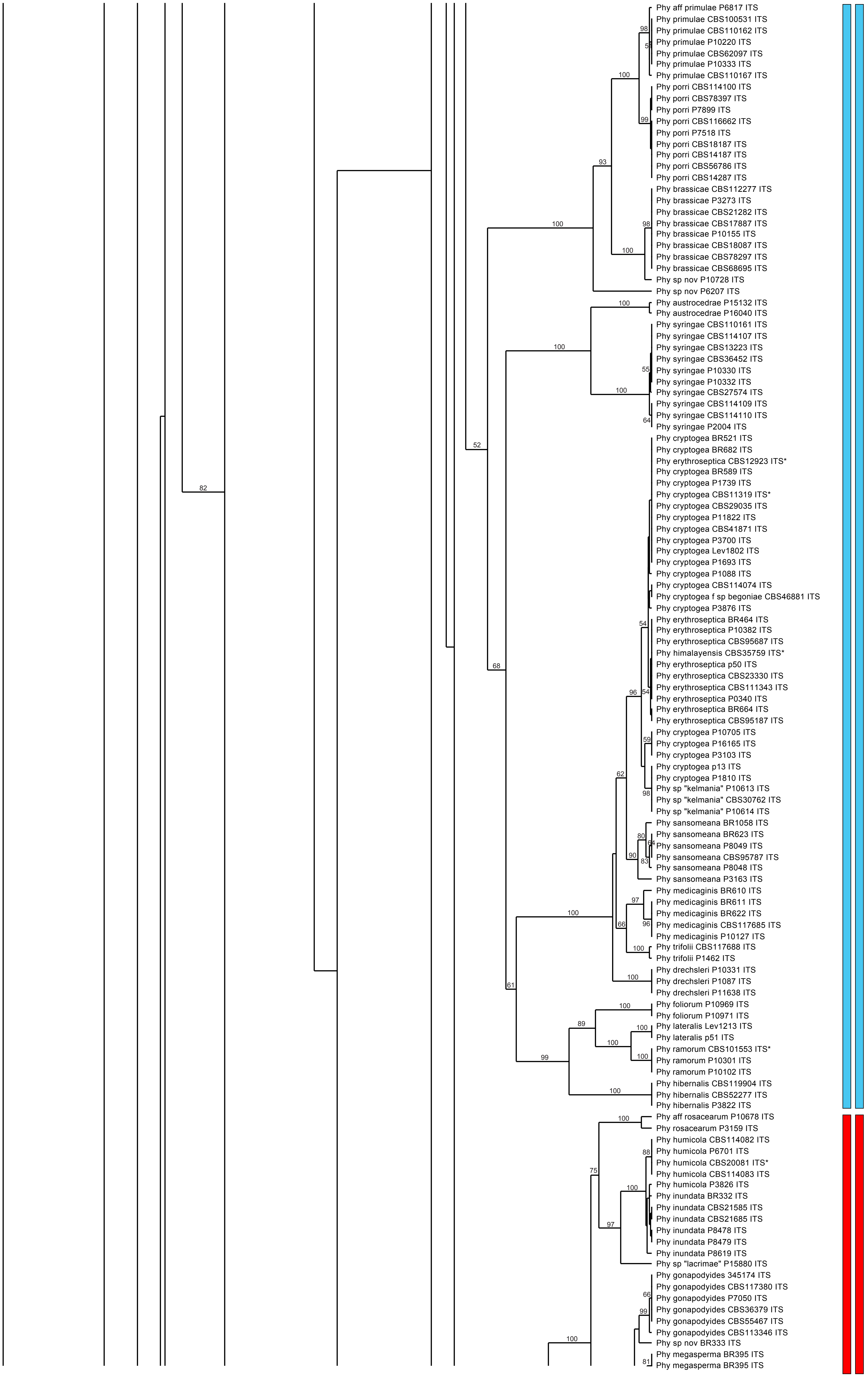
Phy aff primulae P6817 ITS
Phy primulae CBS100531 ITS
Phy primulae CBS110162 ITS
Phy primulae P10220 ITS
Phy primulae CBS62097 ITS
Phy primulae P10333 ITS
Phy primulae CBS110167 ITS
Phy porri CBS114100 ITS
Phy porri CBS78397 ITS
Phy porri P7899 ITS
Phy porri CBS116662 ITS
Phy porri P7518 ITS
Phy porri CBS18187 ITS
Phy porri CBS14187 ITS
Phy porri CBS56786 ITS
Phy porri CBS14287 ITS
Phy brassicae CBS112277 ITS
Phy brassicae P3273 ITS
Phy brassicae CBS21282 ITS
Phy brassicae CBS17887 ITS
Phy brassicae P10155 ITS
Phy brassicae CBS18087 ITS
Phy brassicae CBS78297 ITS
Phy brassicae CBS68695 ITS
Phy sp nov P10728 ITS
Phy sp nov P6207 ITS
Phy austrocedrae P15132 ITS
Phy austrocedrae P16040 ITS
Phy syringae CBS110161 ITS
Phy syringae CBS114107 ITS
Phy syringae CBS13223 ITS
Phy syringae CBS36452 ITS
Phy syringae P10330 ITS
Phy syringae P10332 ITS
Phy syringae CBS27574 ITS
Phy syringae CBS114109 ITS
Phy syringae CBS114110 ITS
Phy syringae P2004 ITS
Phy cryptogea BR521 ITS
Phy cryptogea BR682 ITS
Phy erythroseptica CBS12923 ITS*
Phy cryptogea BR589 ITS
Phy cryptogea P1739 ITS
Phy cryptogea CBS11319 ITS*
Phy cryptogea CBS29035 ITS
Phy cryptogea P11822 ITS
Phy cryptogea CBS41871 ITS
Phy cryptogea P3700 ITS
Phy cryptogea Lev1802 ITS
Phy cryptogea P1693 ITS
Phy cryptogea P1088 ITS
Phy cryptogea CBS114074 ITS
Phy cryptogea f sp begoniae CBS46881 ITS
Phy cryptogea P3876 ITS
Phy erythroseptica BR464 ITS
Phy erythroseptica P10382 ITS
Phy erythroseptica CBS95687 ITS
Phy himalayensis CBS35759 ITS*
Phy erythroseptica p50 ITS
Phy erythroseptica CBS23330 ITS
Phy erythroseptica CBS111343 ITS
Phy erythroseptica P0340 ITS
Phy erythroseptica BR664 ITS
Phy erythroseptica CBS95187 ITS
Phy cryptogea P10705 ITS
Phy cryptogea P16165 ITS
Phy cryptogea P3103 ITS
Phy cryptogea p13 ITS
Phy cryptogea P1810 ITS
Phy sp "kelmania" P10613 ITS
Phy sp "kelmania" CBS30762 ITS
Phy sp "kelmania" P10614 ITS
Phy sansomeana BR1058 ITS
Phy sansomeana BR623 ITS
Phy sansomeana P8049 ITS
Phy sansomeana CBS95787 ITS
Phy sansomeana P8048 ITS
Phy sansomeana P3163 ITS
Phy medicaginis BR610 ITS
Phy medicaginis BR611 ITS
Phy medicaginis BR622 ITS
Phy medicaginis CBS117685 ITS
Phy medicaginis P10127 ITS
Phy trifolii CBS117688 ITS
Phy trifolii P1462 ITS
Phy drechsleri P10331 ITS
Phy drechsleri P1087 ITS
Phy drechsleri P11638 ITS
Phy foliorum P10969 ITS
Phy foliorum P10971 ITS
Phy lateralis Lev1213 ITS
Phy lateralis p51 ITS
Phy ramorum CBS101553 ITS*
Phy ramorum P10301 ITS
Phy ramorum P10102 ITS
Phy hibernalis CBS119904 ITS
Phy hibernalis CBS52277 ITS
Phy hibernalis P3822 ITS
Phy aff rosacearum P10678 ITS
Phy rosacearum P3159 ITS
Phy humicola CBS114082 ITS
Phy humicola P6701 ITS
Phy humicola CBS20081 ITS*
Phy humicola CBS114083 ITS
Phy humicola P3826 ITS
Phy inundata BR332 ITS
Phy inundata CBS21585 ITS
Phy inundata CBS21685 ITS
Phy inundata P8478 ITS
Phy inundata P8479 ITS
Phy inundata P8619 ITS
Phy sp "lacrimae" P15880 ITS
Phy gonapodyides 345174 ITS
Phy gonapodyides CBS117380 ITS
Phy gonapodyides P7050 ITS
Phy gonapodyides CBS36379 ITS
Phy gonapodyides CBS55467 ITS
Phy gonapodyides CBS113346 ITS
Phy sp nov BR333 ITS
Phy megasperma BR395 ITS
Phy megasperma BR395 ITS

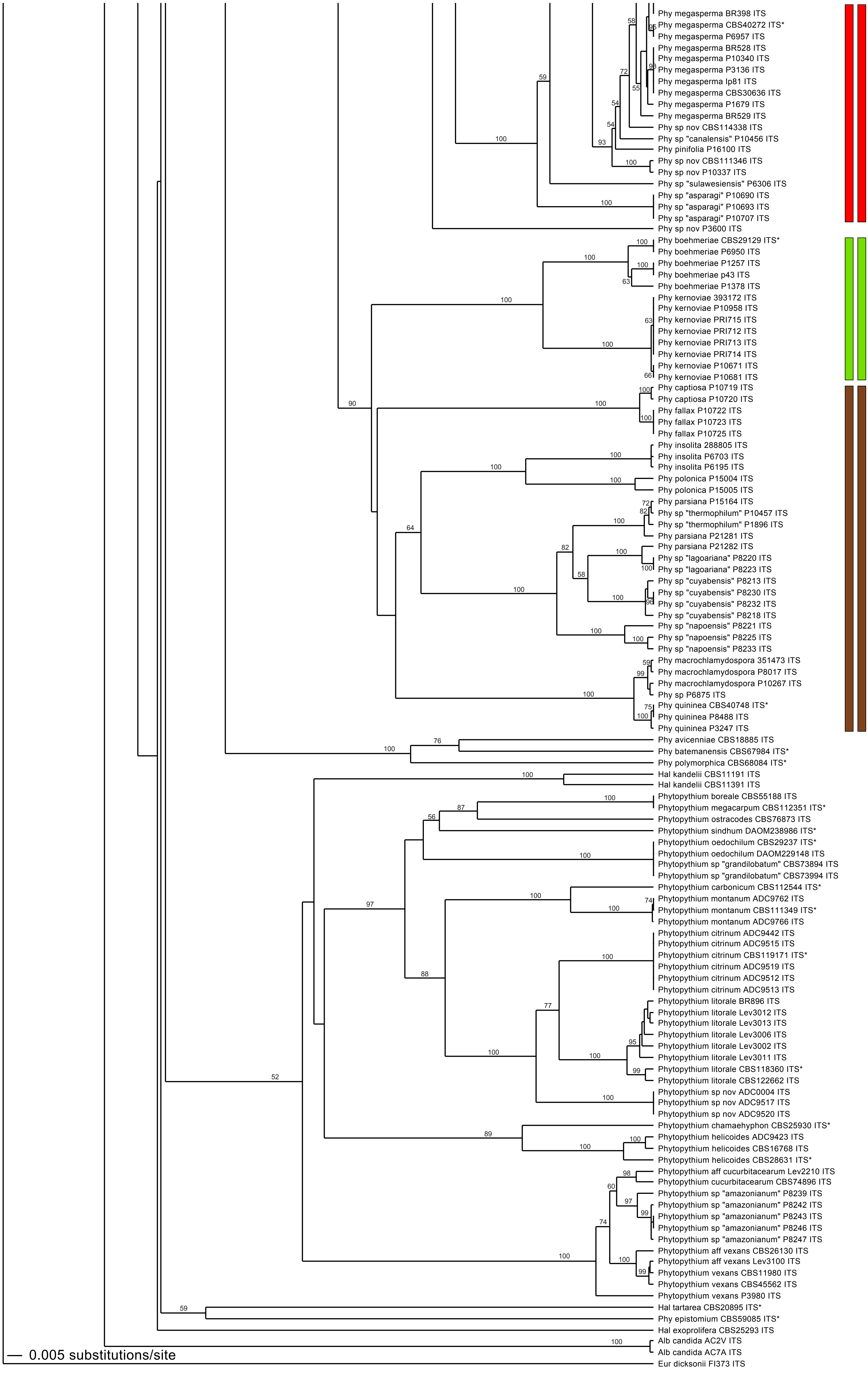
Phy megasperma BR398 ITS
Phy megasperma CBS40272 ITS*
Phy megasperma P6957 ITS
Phy megasperma BR528 ITS
Phy megasperma P10340 ITS
Phy megasperma P3136 ITS
Phy megasperma Ip81 ITS
Phy megasperma CBS30636 ITS
Phy megasperma P1679 ITS
Phy megasperma BR529 ITS
Phy sp nov CBS114338 ITS
Phy sp "canalensis" P10456 ITS
Phy pinifolia P16100 ITS
Phy sp nov CBS111346 ITS
Phy sp nov P10337 ITS
Phy sp "sulawesiensis" P6306 ITS
Phy sp "asparagi" P10690 ITS
Phy sp "asparagi" P10693 ITS
Phy sp "asparagi" P10707 ITS
Phy sp nov P3600 ITS
Phy boehmeriae CBS29129 ITS*
Phy boehmeriae P6950 ITS
Phy boehmeriae P1257 ITS
Phy boehmeriae p43 ITS
Phy boehmeriae P1378 ITS
Phy kernoviae 393172 ITS
Phy kernoviae P10958 ITS
Phy kernoviae PRI715 ITS
Phy kernoviae PRI712 ITS
Phy kernoviae PRI713 ITS
Phy kernoviae PRI714 ITS
Phy kernoviae P10671 ITS
Phy kernoviae P10681 ITS
Phy captiosa P10719 ITS
Phy captiosa P10720 ITS
Phy fallax P10722 ITS
Phy fallax P10723 ITS
Phy fallax P10725 ITS
Phy insolita 288805 ITS
Phy insolita P6703 ITS
Phy insolita P6195 ITS
Phy polonica P15004 ITS
Phy polonica P15005 ITS
Phy parsiana P15164 ITS
Phy sp "thermophilum" P10457 ITS
Phy sp "thermophilum" P1896 ITS
Phy parsiana P21281 ITS
Phy parsiana P21282 ITS
Phy sp "lagoariana" P8220 ITS
Phy sp "lagoariana" P8223 ITS
Phy sp "cuyabensis" P8213 ITS
Phy sp "cuyabensis" P8230 ITS
Phy sp "cuyabensis" P8232 ITS
Phy sp "cuyabensis" P8218 ITS
Phy sp "napoensis" P8221 ITS
Phy sp "napoensis" P8225 ITS
Phy sp "napoensis" P8233 ITS
Phy macrochlamydospora 351473 ITS
Phy macrochlamydospora P8017 ITS
Phy macrochlamydospora P10267 ITS
Phy sp P6875 ITS
Phy quininea CBS40748 ITS*
Phy quininea P8488 ITS
Phy quininea P3247 ITS
Phy avicenniae CBS18885 ITS
Phy batemanensis CBS67984 ITS*
Phy polymorphica CBS68084 ITS*
Hal kandelii CBS11191 ITS
Hal kandelii CBS11391 ITS
Phytopythium boreale CBS55188 ITS
Phytopythium megacarpum CBS112351 ITS*
Phytopythium ostracodes CBS76873 ITS
Phytopythium sindhum DAOM238986 ITS*
Phytopythium oedochilum CBS29237 ITS*
Phytopythium oedochilum DAOM229148 ITS
Phytopythium sp "grandilobatum" CBS73894 ITS
Phytopythium sp "grandilobatum" CBS73994 ITS
Phytopythium carbonicum CBS112544 ITS*
Phytopythium montanum ADC9762 ITS
Phytopythium montanum CBS111349 ITS*
Phytopythium montanum ADC9766 ITS
Phytopythium citrinum ADC9442 ITS
Phytopythium citrinum ADC9515 ITS
Phytopythium citrinum CBS119171 ITS*
Phytopythium citrinum ADC9519 ITS
Phytopythium citrinum ADC9512 ITS
Phytopythium citrinum ADC9513 ITS
Phytopythium litorale BR896 ITS
Phytopythium litorale Lev3012 ITS
Phytopythium litorale Lev3013 ITS
Phytopythium litorale Lev3006 ITS
Phytopythium litorale Lev3002 ITS
Phytopythium litorale Lev3011 ITS
Phytopythium litorale CBS118360 ITS*
Phytopythium litorale CBS122662 ITS
Phytopythium sp nov ADC0004 ITS
Phytopythium sp nov ADC9517 ITS
Phytopythium sp nov ADC9520 ITS
Phytopythium chamaehyphon CBS25930 ITS*
Phytopythium helicoides ADC9423 ITS
Phytopythium helicoides CBS16768 ITS
Phytopythium helicoides CBS28631 ITS*
Phytopythium aff cucurbitacearum Lev2210 ITS
Phytopythium cucurbitacearum CBS74896 ITS
Phytopythium sp "amazonianum" P8239 ITS
Phytopythium sp "amazonianum" P8242 ITS
Phytopythium sp "amazonianum" P8243 ITS
Phytopythium sp "amazonianum" P8246 ITS
Phytopythium sp "amazonianum" P8247 ITS
Phytopythium aff vexans CBS26130 ITS
Phytopythium aff vexans Lev3100 ITS
Phytopythium vexans CBS11980 ITS
Phytopythium vexans CBS45562 ITS
Phytopythium vexans P3980 ITS
Hal tartarea CBS20895 ITS*
Phy epistomium CBS59085 ITS*
Hal exoprolifera CBS25293 ITS
Alb candida AC2V ITS
Alb candida AC7A ITS
Eur dicksonii FI373 ITS
0.005 substitutions/site

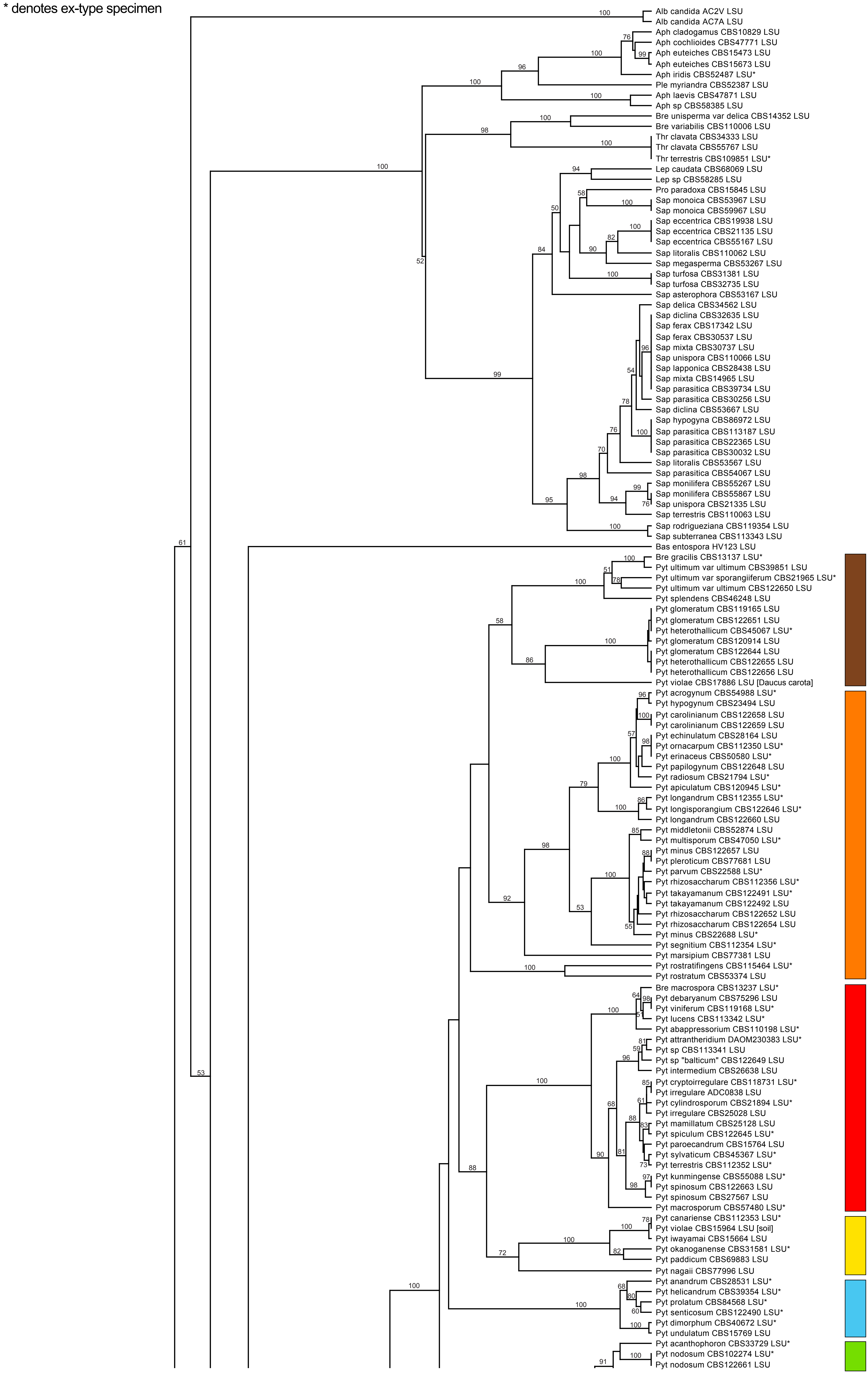

* denotes ex-type specimen
Alb candida AC2V LSU
Alb candida AC7A LSU
Aph cladogamus CBS10829 LSU
Aph cochlioides CBS47771 LSU
Aph euteiches CBS15473 LSU
Aph euteiches CBS15673 LSU
Aph iridis CBS52487 LSU*
Ple myriandra CBS52387 LSU
Aph laevis CBS47871 LSU
Aph sp CBS58385 LSU
Bre unisperma var delica CBS14352 LSU
Bre variabilis CBS110006 LSU
Thr clavata CBS34333 LSU
Thr clavata CBS55767 LSU
Thr terrestris CBS109851 LSU*
Lep caudata CBS68069 LSU
Lep sp CBS58285 LSU
Pro paradoxa CBS15845 LSU
Sap monoica CBS53967 LSU
Sap monoica CBS59967 LSU
Sap eccentrica CBS19938 LSU
Sap eccentrica CBS21135 LSU
Sap eccentrica CBS55167 LSU
Sap litoralis CBS110062 LSU
Sap megasperma CBS53267 LSU
Sap turfosa CBS31381 LSU
Sap turfosa CBS32735 LSU
Sap asterophora CBS53167 LSU
Sap delica CBS34562 LSU
Sap diclina CBS32635 LSU
Sap ferax CBS17342 LSU
Sap ferax CBS30537 LSU
Sap mixta CBS30737 LSU
Sap unispora CBS110066 LSU
Sap lapponica CBS28438 LSU
Sap mixta CBS14965 LSU
Sap parasitica CBS39734 LSU
Sap parasitica CBS30256 LSU
Sap diclina CBS53667 LSU
Sap hypogyna CBS86972 LSU
Sap parasitica CBS113187 LSU
Sap parasitica CBS22365 LSU
Sap parasitica CBS30032 LSU
Sap litoralis CBS53567 LSU
Sap parasitica CBS54067 LSU
Sap monilifera CBS55267 LSU
Sap monilifera CBS55867 LSU
Sap unispora CBS21335 LSU
Sap terrestris CBS110063 LSU
Sap rodrigueziana CBS119354 LSU
Sap subterranea CBS113343 LSU
Bas entospora HV123 LSU
Bre gracilis CBS13137 LSU*
Pyt ultimum var ultimum CBS39851 LSU
Pyt ultimum var sporangiiferum CBS21965 LSU*
Pyt ultimum var ultimum CBS122650 LSU
Pyt splendens CBS46248 LSU
Pyt glomeratum CBS119165 LSU
Pyt glomeratum CBS122651 LSU
Pyt heterothallicum CBS45067 LSU*
Pyt glomeratum CBS120914 LSU
Pyt glomeratum CBS122644 LSU
Pyt heterothallicum CBS122655 LSU
Pyt heterothallicum CBS122656 LSU
Pyt violae CBS17886 LSU [Daucus carota]
Pyt acrogynum CBS54988 LSU*
Pyt hypogynum CBS23494 LSU
Pyt carolinianum CBS122658 LSU
Pyt carolinianum CBS122659 LSU
Pyt echinulatum CBS28164 LSU
Pyt ornacarpum CBS112350 LSU*
Pyt erinaceus CBS50580 LSU*
Pyt papilogynum CBS122648 LSU
Pyt radiosum CBS21794 LSU*
Pyt apiculatum CBS120945 LSU*
Pyt longandrum CBS112355 LSU*
Pyt longisporangium CBS122646 LSU*
Pyt longandrum CBS122660 LSU
Pyt middletonii CBS52874 LSU
Pyt multisporum CBS47050 LSU*
Pyt minus CBS122657 LSU
Pyt pleroticum CBS77681 LSU
Pyt parvum CBS22588 LSU*
Pyt rhizosaccharum CBS112356 LSU*
Pyt takayamanum CBS122491 LSU*
Pyt takayamanum CBS122492 LSU
Pyt rhizosaccharum CBS122652 LSU
Pyt rhizosaccharum CBS122654 LSU
Pyt minus CBS22688 LSU*
Pyt segnitium CBS112354 LSU*
Pyt marsipium CBS77381 LSU
Pyt rostratifingens CBS115464 LSU*
Pyt rostratum CBS53374 LSU
Bre macrospora CBS13237 LSU*
Pyt debaryanum CBS75296 LSU
Pyt viniferum CBS119168 LSU*
Pyt lucens CBS113342 LSU*
Pyt abappressorium CBS110198 LSU*
Pyt attrantheridium DAOM230383 LSU*
Pyt sp CBS113341 LSU
Pyt sp "balticum" CBS122649 LSU
Pyt intermedium CBS26638 LSU
Pyt cryptoirregulare CBS118731 LSU*
Pyt irregulare ADC0838 LSU
Pyt cylindrosporum CBS21894 LSU*
Pyt irregulare CBS25028 LSU
Pyt mamillatum CBS25128 LSU
Pyt spiculum CBS122645 LSU*
Pyt paroecandrum CBS15764 LSU
Pyt sylvaticum CBS45367 LSU*
Pyt terrestris CBS112352 LSU*
Pyt kunmingense CBS55088 LSU*
Pyt spinosum CBS122663 LSU
Pyt spinosum CBS27567 LSU
Pyt macrosporum CBS57480 LSU*
Pyt canariense CBS112353 LSU*
Pyt violae CBS15964 LSU [soil]
Pyt iwayamai CBS15664 LSU
Pyt okanoganense CBS31581 LSU*
Pyt paddicum CBS69883 LSU
Pyt nagaii CBS77996 LSU
Pyt anandrum CBS28531 LSU*
Pyt helicandrum CBS39354 LSU*
Pyt prolatum CBS84568 LSU*
Pyt senticosum CBS122490 LSU*
Pyt dimorphum CBS40672 LSU*
Pyt undulatum CBS15769 LSU
Pyt acanthophoron CBS33729 LSU*
Pyt nodosum CBS102274 LSU*
Pyt nodosum CBS122661 LSU

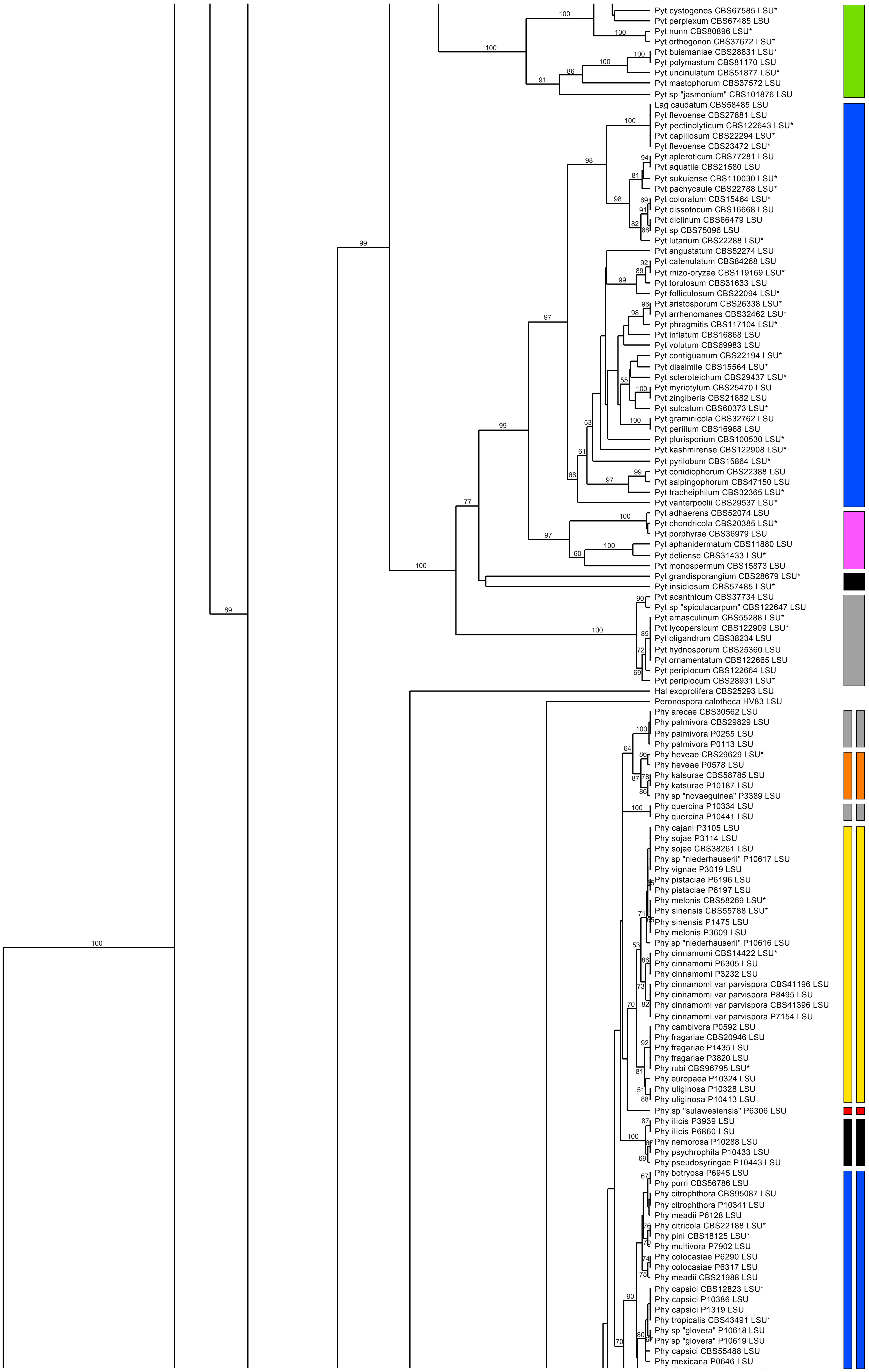

Pyt cystogenes CBS67585 LSU*
Pyt perplexum CBS67485 LSU
Pyt nunn CBS80896 LSU*
Pyt orthogonon CBS37672 LSU*
Pyt buismaniae CBS28831 LSU*
Pyt polymastum CBS81170 LSU
Pyt uncinulatum CBS51877 LSU*
Pyt mastophorum CBS37572 LSU
Pyt sp "jasmonium" CBS101876 LSU
Lag caudatum CBS58485 LSU
Pyt flevoense CBS27881 LSU
Pyt pectinolyticum CBS122643 LSU*
Pyt capillosum CBS22294 LSU*
Pyt flevoense CBS23472 LSU*
Pyt apleroticum CBS77281 LSU
Pyt aquatile CBS21580 LSU
Pyt sukuiense CBS110030 LSU*
Pyt pachycaule CBS22788 LSU*
Pyt coloratum CBS15464 LSU*
Pyt dissotocum CBS16668 LSU
Pyt diclinum CBS66479 LSU
Pyt sp CBS75096 LSU
Pyt lutarium CBS22288 LSU*
Pyt angustatum CBS52274 LSU
Pyt catenulatum CBS84268 LSU
Pyt rhizo-oryzae CBS119169 LSU*
Pyt torulosum CBS31633 LSU
Pyt folliculosum CBS22094 LSU*
Pyt aristosporum CBS26338 LSU*
Pyt arrhenomanes CBS32462 LSU*
Pyt phragmitis CBS117104 LSU*
Pyt inflatum CBS16868 LSU
Pyt volutum CBS69983 LSU
Pyt contiguanum CBS22194 LSU*
Pyt dissimile CBS15564 LSU*
Pyt scleroteichum CBS29437 LSU*
Pyt myriotylum CBS25470 LSU
Pyt zingiberis CBS21682 LSU
Pyt sulcatum CBS60373 LSU*
Pyt graminicola CBS32762 LSU
Pyt periilum CBS16968 LSU
Pyt plurisporium CBS100530 LSU*
Pyt kashmirense CBS122908 LSU*
Pyt pyrilobum CBS15864 LSU*
Pyt conidiophorum CBS22388 LSU
Pyt salpingophorum CBS47150 LSU
Pyt tracheiphilum CBS32365 LSU*
Pyt vanterpoolii CBS29537 LSU*
Pyt adhaerens CBS52074 LSU
Pyt chondricola CBS20385 LSU*
Pyt porphyrae CBS36979 LSU
Pyt aphanidermatum CBS11880 LSU
Pyt deliense CBS31433 LSU*
Pyt monospermum CBS15873 LSU
Pyt grandisporangium CBS28679 LSU*
Pyt insidiosum CBS57485 LSU*
Pyt acanthicum CBS37734 LSU
Pyt sp "spiculacarpum" CBS122647 LSU
Pyt amasculinum CBS55288 LSU*
Pyt lycopersicum CBS122909 LSU*
Pyt oligandrum CBS38234 LSU
Pyt hydnosporum CBS25360 LSU
Pyt ornamentatum CBS122665 LSU
Pyt periplocum CBS122664 LSU
Pyt periplocum CBS28931 LSU*
Hal exoprolifera CBS25293 LSU
Peronospora calotheca HV83 LSU
Phy arecae CBS30562 LSU
Phy palmivora CBS29829 LSU
Phy palmivora P0255 LSU
Phy palmivora P0113 LSU
Phy heveae CBS29629 LSU*
Phy heveae P0578 LSU
Phy katsurae CBS58785 LSU
Phy katsurae P10187 LSU
Phy sp "novaeguinea" P3389 LSU
Phy quercina P10334 LSU
Phy quercina P10441 LSU
Phy cajani P3105 LSU
Phy sojae P3114 LSU
Phy sojae CBS38261 LSU
Phy sp "niederhauserii" P10617 LSU
Phy vignae P3019 LSU
Phy pistaciae P6196 LSU
Phy pistaciae P6197 LSU
Phy melonis CBS58269 LSU*
Phy sinensis CBS55788 LSU*
Phy sinensis P1475 LSU
Phy melonis P3609 LSU
Phy sp "niederhauserii" P10616 LSU
Phy cinnamomi CBS14422 LSU*
Phy cinnamomi P6305 LSU
Phy cinnamomi P3232 LSU
Phy cinnamomi var parvispora CBS41196 LSU
Phy cinnamomi var parvispora P8495 LSU
Phy cinnamomi var parvispora CBS41396 LSU
Phy cinnamomi var parvispora P7154 LSU
Phy cambivora P0592 LSU
Phy fragariae CBS20946 LSU
Phy fragariae P1435 LSU
Phy fragariae P3820 LSU
Phy rubi CBS96795 LSU*
Phy europaea P10324 LSU
Phy uliginosa P10328 LSU
Phy uliginosa P10413 LSU
Phy sp "sulawesiensis" P6306 LSU
Phy ilicis P3939 LSU
Phy ilicis P6860 LSU
Phy nemorosa P10288 LSU
Phy psychrophila P10433 LSU
Phy pseudosyringae P10443 LSU
Phy botryosa P6945 LSU
Phy porri CBS56786 LSU
Phy citrophthora CBS95087 LSU
Phy citrophthora P10341 LSU
Phy meadii P6128 LSU
Phy citricola CBS22188 LSU*
Phy pini CBS18125 LSU*
Phy multivora P7902 LSU
Phy colocasiae P6290 LSU
Phy colocasiae P6317 LSU
Phy meadii CBS21988 LSU
Phy capsici CBS12823 LSU*
Phy capsici P10386 LSU
Phy capsici P1319 LSU
Phy tropicalis CBS43491 LSU*
Phy sp "glovera" P10618 LSU
Phy sp "glovera" P10619 LSU
Phy capsici CBS55488 LSU
Phy mexicana P0646 LSU

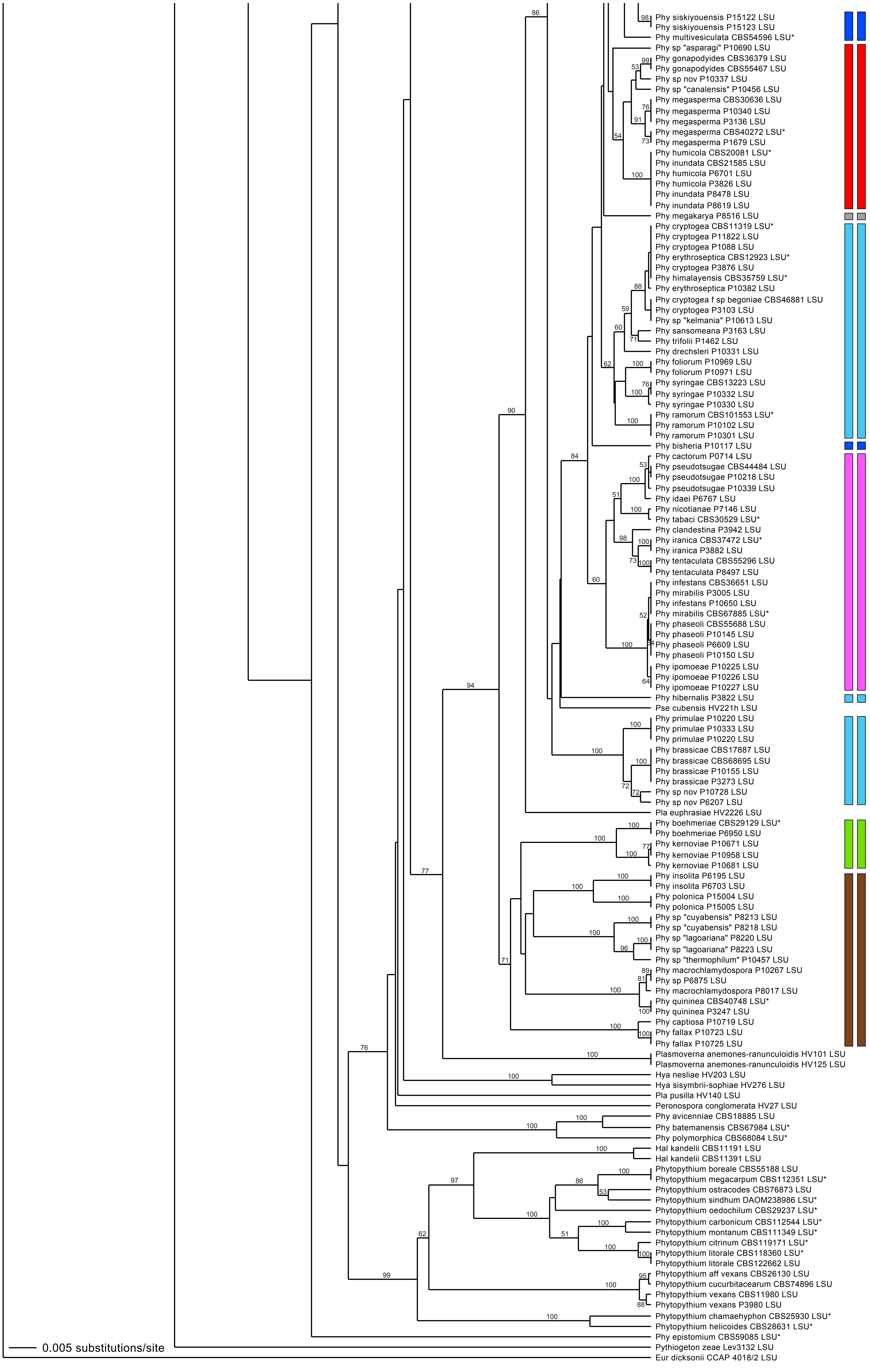
Phy siskiyouensis P15122 LSU
Phy siskiyouensis P15123 LSU
Phy multivesiculata CBS54596 LSU*
Phy sp "asparagi" P10690 LSU
Phy gonapodyides CBS36379 LSU
Phy gonapodyides CBS55467 LSU
Phy sp nov P10337 LSU
Phy sp "canalensis" P10456 LSU
Phy megasperma CBS30636 LSU
Phy megasperma P10340 LSU
Phy megasperma P3136 LSU
Phy megasperma CBS40272 LSU*
Phy megasperma P1679 LSU
Phy humicola CBS20081 LSU*
Phy inundata CBS21585 LSU
Phy humicola P6701 LSU
Phy humicola P3826 LSU
Phy inundata P8478 LSU
Phy inundata P8619 LSU
Phy megakarya P8516 LSU
Phy cryptogea CBS11319 LSU*
Phy cryptogea P11822 LSU
Phy cryptogea P1088 LSU
Phy erythroseptica CBS12923 LSU*
Phy cryptogea P3876 LSU
Phy himalayensis CBS35759 LSU*
Phy erythroseptica P10382 LSU
Phy cryptogea f sp begoniae CBS46881 LSU
Phy cryptogea P3103 LSU
Phy sp "kelmania" P10613 LSU
Phy sansomeana P3163 LSU
Phy trifolii P1462 LSU
Phy drechsleri P10331 LSU
Phy foliorum P10969 LSU
Phy foliorum P10971 LSU
Phy syringae CBS13223 LSU
Phy syringae P10332 LSU
Phy syringae P10330 LSU
Phy ramorum CBS101553 LSU*
Phy ramorum P10102 LSU
Phy ramorum P10301 LSU
Phy bisheria P10117 LSU
Phy cactorum P0714 LSU
Phy pseudotsugae CBS44484 LSU
Phy pseudotsugae P10218 LSU
Phy pseudotsugae P10339 LSU
Phy idaei P6767 LSU
Phy nicotianae P7146 LSU
Phy tabaci CBS30529 LSU*
Phy clandestina P3942 LSU
Phy iranica CBS37472 LSU*
Phy iranica P3882 LSU
Phy tentaculata CBS55296 LSU
Phy tentaculata P8497 LSU
Phy infestans CBS36651 LSU
Phy mirabilis P3005 LSU
Phy infestans P10650 LSU
Phy mirabilis CBS67885 LSU*
Phy phaseoli CBS55688 LSU
Phy phaseoli P10145 LSU
Phy phaseoli P6609 LSU
Phy phaseoli P10150 LSU
Phy ipomoeae P10225 LSU
Phy ipomoeae P10226 LSU
Phy ipomoeae P10227 LSU
Phy hibernalis P3822 LSU
Pse cubensis HV221h LSU
Phy primulae P10220 LSU
Phy primulae P10333 LSU
Phy primulae P10220 LSU
Phy brassicae CBS17887 LSU
Phy brassicae CBS68695 LSU
Phy brassicae P10155 LSU
Phy brassicae P3273 LSU
Phy sp nov P10728 LSU
Phy sp nov P6207 LSU
Pla euphrasiae HV2226 LSU
Phy boehmeriae CBS29129 LSU*
Phy boehmeriae P6950 LSU
Phy kernoviae P10671 LSU
Phy kernoviae P10958 LSU
Phy kernoviae P10681 LSU
Phy insolita P6195 LSU
Phy insolita P6703 LSU
Phy polonica P15004 LSU
Phy polonica P15005 LSU
Phy sp "cuyabensis" P8213 LSU
Phy sp "cuyabensis" P8218 LSU
Phy sp "lagoariana" P8220 LSU
Phy sp "lagoariana" P8223 LSU
Phy sp "thermophilum" P10457 LSU
Phy macrochlamydospora P10267 LSU
Phy sp P6875 LSU
Phy macrochlamydospora P8017 LSU
Phy quininea CBS40748 LSU*
Phy quininea P3247 LSU
Phy captiosa P10719 LSU
Phy fallax P10723 LSU
Phy fallax P10725 LSU
Plasmoverna anemones-ranunculoidis HV101 LSU
Plasmoverna anemones-ranunculoidis HV125 LSU
Hya nesliae HV203 LSU
Hya sisymbrii-sophiae HV276 LSU
Pla pusilla HV140 LSU
Peronospora conglomerata HV27 LSU
Phy avicenniae CBS18885 LSU
Phy batemanensis CBS67984 LSU*
Phy polymorphica CBS68084 LSU*
Hal kandelii CBS11191 LSU
Hal kandelii CBS11391 LSU
Phytopythium boreale CBS55188 LSU
Phytopythium megacarpum CBS112351 LSU*
Phytopythium ostracodes CBS76873 LSU
Phytopythium sindhum DAOM238986 LSU*
Phytopythium oedochilum CBS29237 LSU*
Phytopythium carbonicum CBS112544 LSU*
Phytopythium montanum CBS111349 LSU*
Phytopythium citrinum CBS119171 LSU*
Phytopythium litorale CBS118360 LSU*
Phytopythium litorale CBS122662 LSU
Phytopythium aff vexans CBS26130 LSU
Phytopythium cucurbitacearum CBS74896 LSU
Phytopythium vexans CBS11980 LSU
Phytopythium vexans P3980 LSU
Phytopythium chamaehyphon CBS25930 LSU*
Phytopythium helicoides CBS28631 LSU*
Phy epistomium CBS59085 LSU*
Pythiogeton zeae Lev3132 LSU
Eur dicksonii CCAP 4018/2 LSU
0.005 substitutions/site
